# Supplementary material for: Noncovarying storage effect: Balancing and positive directional selection on mutant alleles that amplify random fitness and demographic fluctuations
Source: PLoS One. 2025 Jul 22;20(7):e0328130. doi: 10.1371/journal.pone.0328130 (PMC12282919; doi:10.1371/journal.pone.0328130)
Supplement: S1 Appendix — (PDF) [file pone.0328130.s001.pdf]

Supporting Information

For "Noncovarying storage effect: balancing and positive directional selection on mutant alleles that amplify random fitness and demographic fluctuations"

by Yuseob Kim, Ewha Womans University

S1 Appendix. Derivation of conditions for balancing and directional selection on A_2 in the TP model

Here only deterministic changes in allele frequencies are tracked as a very large population is assumed. At the end of reproduction but before migration into subpopulations at generation t - 1, there are n_1 and n_2 copies (individuals) of A_1 and A_2 in the entire population. After migration, the numbers of A_1 and A_2 copies are $n_{1F} = (1-r)n_1$ and $n_{2F} = (1-r)n_2$ in the field and $n_{1R} = rn_1$ and $n_{2R} = rn_2$ in the refuge. Therefore, the relative frequencies of alleles are identical between the field and refuge before reproduction at the beginning of generation t.

Soft selection is first considered. Then, each A_2 allele produces $(1+S_t)K_F^{(t)}/\bar{N}_F^{(t)}$ copies of descendants, where $\bar{N}_F^{(t)} = n_{1F} + (1+S_t)n_{2F} = (1-r)(n_1 + (1+S_t)n_2)$. Since $n_1 \gg n_2$ after A_2 appears by mutation, $n_1 + (1+S_t)n_2 \approx n_1$ and $\bar{N}_F^{(t)} \approx \tilde{N}_F^{(t)} = (1-r)(n_1+n_2) = (1-r)\left(K_F^{(t-1)} + K_R^{(t-1)}\right)$. Similar approximations apply to the refuge. Let n'_2 be the expected number of A_2 in generation t. This leads to eq. (1). Namely,

$$\frac{n'_2}{n_2} = (1-r)\frac{(1+S_t)K_F^{(t)}}{\bar{N}_F^{(t)}} + r\frac{(1+S_t^*)K_R^{(t)}}{\bar{N}_R^{(t)}} \approx (1-r)\frac{(1+S_t)K_F^{(t)}}{\tilde{N}_F^{(t)}} + r\frac{(1+S_t^*)K_R^{(t)}}{\tilde{N}_R^{(t)}} = \frac{(1+S_t)K_F^{(t)} + (1+S_t^*)K_R^{(t)}}{K_F^{(t-1)} + K_R^{(t-1)}}$$

Then, with $K_F^{(t-1)} = K_{F0}\mathrm{e}^U$, $K_R^{(t-1)} = K_{R0}\mathrm{e}^V$, $K_F^{(t)} = K_{F0}\mathrm{e}^{U'}$, and $K_R^{(t)} = K_{R0}\mathrm{e}^{V'}$,

$$\Delta_2 \equiv \log\left[\frac{n'_2}{n_2}\right] = \log\left[\mathrm{e}^{U'}(1+S) + \rho\mathrm{e}^{V'}(1+S^*)\right] - \log[\mathrm{e}^U + \rho\mathrm{e}^V],$$

where $S = S_t$, $S^* = S_t^*$, and $\rho = K_{R0}/K_{F0}$. If $\mathrm{E}[\Delta_2] > 0$, where the expectation is over demographic and fitness fluctuations, the rare allele A_2 is expected to increase its copy number over time. As the expectations of U, V, U', V', S, and S^* are all zero and non-zero correlations exist only among U', V', S, and S^* and between U and V, from the Taylor expansion of Δ_2 up to the

second order, it can be shown that

$$\mathrm{E}[\Delta_2] \approx \frac{1}{2}\alpha_{U'U'}\mathrm{Var}[U'] + \frac{1}{2}\alpha_{V'V'}\mathrm{Var}[V'] + \frac{1}{2}\alpha_{UU}\mathrm{Var}[U] + \frac{1}{2}\alpha_{VV}\mathrm{Var}[V] + \frac{1}{2}\alpha_{SS}\mathrm{Var}[S] + \frac{1}{2}\alpha_{S^*S^*}\mathrm{Var}[S^*] + \alpha_{U'V'}\mathrm{Cov}[U',V'] + \alpha_{UV}\mathrm{Cov}[U,V] + \alpha_{SS^*}\mathrm{Cov}[S,S^*] + \alpha_{SU'}\mathrm{Cov}[S,U'] + \alpha_{SV'}\mathrm{Cov}[S,V'] + \alpha_{S^*U'}\mathrm{Cov}[S^*,U'] + \alpha_{S^*V'}\mathrm{Cov}[S^*,V']$$

where $\alpha_{XY} = \frac{\partial^2\Delta_2}{\partial X \partial Y}$ is the Taylor coefficient for variables X and Y evaluated at $(U,V,U',V',S,S^*) = (0,0,0,0,0,0)$. Since $\alpha_{U'U'} = \alpha_{V'V'} = \alpha_{UV} = \alpha_{SU'} = \alpha_{S^*V'} = \frac{\rho}{(1+\rho)^2}$, $\alpha_{UU} = \alpha_{VV} = \alpha_{U'V'} = \alpha_{SV'} = \alpha_{S^*U'} = \alpha_{SS^*} = -\frac{\rho}{(1+\rho)^2}$, $\alpha_{SS} = -\frac{1}{(1+\rho)^2}$, $\alpha_{S^*S^*} = -\frac{\rho^2}{(1+\rho)^2}$, $\mathrm{Var}[U] = \mathrm{Var}[U']$, $\mathrm{Var}[V] = \mathrm{Var}[V']$, and $\mathrm{Cov}[U,V] = \mathrm{Cov}[U',V']$, the above equation is simplified to

$$\mathrm{E}[\Delta_2] = \frac{2\rho\mathrm{Cov}[S-S^*,U'-V'] - \mathrm{Var}[S] - \rho^2\mathrm{Var}[S^*] - 2\rho\mathrm{Cov}[S,S^*]}{2(1+\rho)^2}.$$

Therefore, the condition for the A_2 allele being positively selected (i.e. invading the population initially fixed for A_1), is $\mathrm{E}[\Delta_2] > 0$ or

$$\frac{\mathrm{Cov}[S-S^*,U'-V']}{\mathrm{Var}[S] + \rho^2\mathrm{Var}[S^*] + 2\rho\mathrm{Cov}[S,S^*]} > \frac{1}{2\rho}$$

assuming $(\mathrm{Var}[S] + \rho^2\mathrm{Var}[S^*])/\rho > -2\mathrm{Cov}[S,S^*]$. If $S_t^* = \delta S_t$, the above condition becomes

$$\Phi = \frac{\mathrm{Cov}[S,U'-V']}{\mathrm{Var}[S]} = \frac{\mathrm{Cov}[S,\log[K_F/K_R]]}{\mathrm{Var}[S]} > \frac{(1+\delta\rho)^2}{2(1-\delta)\rho}.$$

A similar result can be obtained for the condition for the A_1 allele to invade a population initially fixed for A_2. The expected change in the number of A_1, n'_1/n_1, is obtained with a procedure identical to that leading to eq. (1) except that the relative fitness is flipped. Therefore

$$\frac{n_1'}{n_1} \approx \frac{K_F^{(t)}}{K_F^{(t-1)}+K_R^{(t-1)}}\frac{1}{1+S_t} + \frac{K_R^{(t)}}{K_F^{(t-1)}+K_R^{(t-1)}}\frac{1}{1+S_t^*} = \frac{(1+S_t^*)\mathrm{e}^{U'} + (1+S_t)\rho\mathrm{e}^{V'}}{(1+S_t)(1+S_t^*)(\mathrm{e}^U+\rho\mathrm{e}^V)}$$

for $n_1 \ll n_2$, the dynamics of the rare A_1 allele depends on the expectation of

$$\Delta_{1,\mathrm{soft}} \equiv \log\left[\frac{n_1'}{n_1}\right] = \log\left[(1+S_t^*)\mathrm{e}^{U'} + (1+S)\rho\mathrm{e}^{V'}\right] - \log[(1+S)(1+S_t^*)(\mathrm{e}^U + \rho\mathrm{e}^V)].$$

Applying the quadratic approximation used for obtain $\mathrm{E}[\Delta_2]$ above, one obtains

$$\mathrm{E}[\Delta_{1,\mathrm{soft}}] = \frac{(1+2\rho)\mathrm{Var}[S] + (2\rho+\rho^2)\mathrm{Var}[S^*] - 2\rho\mathrm{Cov}[S,S^*] - 2\rho\mathrm{Cov}[S-S^*,U'-V']}{2(1+\rho)^2}$$

The condition for the rare A_1 allele to increase in frequency is $\mathrm{E}[\Delta_{1,\mathrm{soft}}] > 0$ or

$$\frac{\mathrm{Cov}[S-S^*, U'-V']}{(1+2\rho)\mathrm{Var}[S]+(2\rho+\rho^2)\mathrm{Var}[S^*]-2\rho\mathrm{Cov}[S,S^*]} < \frac{1}{2\rho}.$$

If $S_t^* = \delta S_t$, the above condition becomes

$$\Phi < 1-\delta+\frac{(1+\delta\rho)^2}{2(1-\delta)\rho}.$$

With hard selection, the frequency change of A_2 when rare is again given by eq. (1). Therefore, the condition for A_2 invading the population fixed for A_1 ($\mathrm{E}[\Delta_2] > 0$ above) is no different from that for soft selection. However, because the carrying capacities of the field and refuge fixed for A_2 at generation t is given by $(1+S_t)K_F^{(t)}$ and $(1+S_t^*)K_R^{(t)}$ and $\tilde{N}_F^{(t)} = (1-r)((1+S_{t-1})K_F^{(t-1)} + (1+S_{t-1}^*)K_R^{(t-1)})$ and $\tilde{N}_R^{(t)} = r((1+S_{t-1})K_F^{(t-1)} + (1+S_{t-1}^*)K_R^{(t-1)})$ under hard selection, the opposite condition for A_1 invading A_2 is given by

$$\frac{n_1'}{n_1} = (1-r)\frac{(1+S_t)K_F^{(t)}}{\tilde{N}_F^{(t)}}\frac{1}{1+S_t} + r\frac{(1+S_t^*)K_R^{(t)}}{\tilde{N}_R^{(t)}}\frac{1}{1+S_t^*} = \frac{K_F^{(t)}+K_R^{(t)}}{(1+S_{t-1})K_F^{(t-1)}+(1+S_{t-1}^*)K_R^{(t-1)}} = \frac{e^{U'}+\rho e^{V'}}{(1+S_{t-1})e^{U}+(1+S_{t-1}^*)\rho e^{V}}$$

Then,

$$\Delta_{1,\mathrm{hard}} \equiv \log\left[\frac{n_1'}{n_1}\right] = \log[\mathrm{e}^{U'}+\rho\mathrm{e}^{V'}] - \log[(1+S_{t-1})\mathrm{e}^{U} + (1+S_{t-1}^*)\rho\mathrm{e}^{V}].$$

Now, given that (U', V', S_t, S_t^*) and $(U, V, S_{t-1}, S_{t-1}^*)$ are independent samples of a joint distribution, the expectation of $-\Delta_{1,\mathrm{hard}}$ is exactly that of Δ_2 above. Therefore, the condition for the fixation of A_2 ($\mathrm{E}[\Delta_{1,\mathrm{hard}}] < 0$) is exactly the condition for positive selection on a rare A_2 ($\mathrm{E}[\Delta_2] > 0$).
