# Supplementary material for: Noncovarying storage effect: Balancing and positive directional selection on mutant alleles that amplify random fitness and demographic fluctuations
Source: PLoS One. 2025 Jul 22;20(7):e0328130. doi: 10.1371/journal.pone.0328130 (PMC12282919; doi:10.1371/journal.pone.0328130)
Supplement: S2 Appendix — (PDF) [file pone.0328130.s002.pdf]

Supporting Information

For "Noncovarying storage effect: balancing and positive directional selection on mutant alleles that amplify random fitness and demographic fluctuations"

by Yuseob Kim, Ewha Womans University

S2 Appendix. Methods of stochastic simulation

Stochastic simulations of the single- and multi-locus eco-evolutionary models were implemented in Mathematica ver. 11.3. In both TP and LSA models, an individual has L loci that are arranged linearly with probability c for recombination between adjacent loci per generation. The simulation keeps track of two sets of lists: one containing the sets of haplotypes carried by non-zero individuals in the field and in the refuge ($\boldsymbol{H}_F$ and $\boldsymbol{H}_R$) and the other containing the corresponding numbers of individuals ($\boldsymbol{N}_F$ and $\boldsymbol{N}_R$).

In the TP model, generation t begins after the migration step (re-distribution of individuals produced in the previous generation into the field and refuge) is completed. Haplotype numbers are first updated by mutation and recombination. A Poisson variate with mean $NL\mu$, where N is the current size of a subpopulation at hand, is drawn as the total number of mutation events and each event is assigned to haplotypes and loci proportional to their current frequencies. Similarly, for $L > 1$, a Poisson variate with mean $N(L\text{-}1)c$ is drawn as the total number of recombination events in the subpopulation. For each recombination event, two haplotypes are chosen proportional to their frequencies and one of the L-1 intervals is chosen to be the position of crossing-over between the two haplotypes. Newly created or lost haplotypes during mutation and recombination steps are tracked by updating ($\boldsymbol{H}_F$, $\boldsymbol{H}_R$) and ($\boldsymbol{N}_F$, $\boldsymbol{N}_R$). Then, the updated lists are further updated in the step of reproduction according to their fitness. Under soft selection, the number of individuals with haplotype i born in the next generation is Poisson distributed with mean $W_i \tilde{N}_{F,i}^{(t)} K_F^{(t)} / \sum_j W_j \tilde{N}_{F,j}^{(t)}$ in the field and $\tilde{N}_{R,i}^{(t)} K_R^{(t)} / \sum_j \tilde{N}_{R,j}^{(t)}$ in the refuge, where W_i is the relative fitness of haplotype i and $\tilde{N}_{F,i}^{(t)}$ ($\tilde{N}_{R,i}^{(t)}$) is the number of haplotype i individuals in the field (refuge) after the haplotypes are updated by mutation and recombination. Under hard selection, the corresponding mean number in the field is $W_i \tilde{N}_{F,i}^{(t)} K_F^{(t)} / \sum_j \tilde{N}_{F,j}^{(t)}$. Finally, when there are N_F and N_R individuals in the field and refuge, Poisson variates with the mean $N_F m_{FR}$ ($N_R m_{RF}$) truncated

at the upper bound N_F (N_R) is drawn to determine the numbers of migrants from field to refuge (from refuge to field). Migrants are chosen proportional to the haplotype frequencies and then merged with the residents of the destination. This completes a single-generation iteration in updating haplotype lists.

Simulation for the LSA model is similarly performed by tracking the haplotypes. Let l_i, m_i, and n_i be the number of individuals with haplotype i (i = 1, …, 2^L) at the larval, subadult, and adult stages at time t. The following steps are taken to produce the corresponding numbers, l_i', m_i', and n_i', at time t+1. As in the TP model, bidirectional mutations with probability μ per locus and recombination with probability c for adjacent loci occur but in subadult and adult stages only. Recombination must occur among subadults and among adults only. The updated numbers of individuals by haplotypes in subadults and adults after mutations and recombination are m_i*, and n_i*. Next, the numbers of individuals by haplotypes in the next generation are obtained. l_i' is given by a Poisson number with mean $W_i^{(t)}(m_i^* + n_i^*)K_L^{(t)}/\sum_i(W_i^{(t)}(m_i^* + n_i^*))$ under soft selection and $W_i^{(t)}(m_i^* + n_i^*)K_L^{(t)}/\sum_i(m_i^* + n_i^*)$ under hard selection. m_i' and n_i' are given by Poisson numbers with mean $e_L l_i$ and $e_L e_S m_i$*, respectively.
