# Supplementary material for: Noncovarying storage effect: Balancing and positive directional selection on mutant alleles that amplify random fitness and demographic fluctuations
Source: PLoS One. 2025 Jul 22;20(7):e0328130. doi: 10.1371/journal.pone.0328130 (PMC12282919; doi:10.1371/journal.pone.0328130)
Supplement: S1 Table — (PDF) [file pone.0328130.s003.pdf]

Supporting Information

For "Noncovarying storage effect: balancing and positive directional selection on mutant alleles that amplify random fitness and demographic fluctuations"

by Yuseob Kim, Ewha Womans University

S1 Tables

Table A. One-locus simulation with variable migration rates in the TP model

mode	m_{FR}	m_{RF}	$\bar{H}$	P_{01}	P_{90}
neutral ($a_S = b_S = 0$, $a_U = 0.3$, $a_V = 0.05$, $b_U = b_V = 0.1$)	[0.495, 0.505]	[0.495, 0.505]	0.0633	0.60	0.24
	[0.1, 0.2]	[0.1, 0.2]	0.0657	0.61	0.24
soft selection, Φ = 1 ($a_S = 0.2$, $b_S = 0.1$, $a_U = 0.3$, $a_V = 0.05$, $b_U = b_V = 0.1$)	[0.495, 0.505]	[0.495, 0.505]	0.196	0.28	0.25
	[0.3, 0.7]	[0.3, 0.7]	0.177	0.45	0.14
	[0.3, 0.5]	[0.5, 0.7]	0.135	0.42	0.26
	[0.5, 0.7]	[0.3, 0.5]	0.147	0.42	0.25
	[0.1, 0.5]	[0.1, 0.5]	0.142	0.59	0.08
	[0.2, 0.4]	[0.2, 0.4]	0.118	0.70	0.03
	[0.1, 0.2]	[0.1, 0.2]	0.056	0.88	0.00
	[0.1. 0.2]	[0.4, 0.5]	0.186	0.34	0.22
	[0.4, 0.5]	[0.1, 0.2]	0.052	0.88	0.01
Hard selection, Φ = 0.8 ($a_S = 0.2$, $b_S = 0.15$, $a_U = 0.3$, $a_V = 0.05$, $b_U = b_V = 0.1$)	[0.495, 0.505]	[0.495, 0.505]	0.030	0.05	0.89
	[0.3, 0.7]	[0.3, 0.7]	0.020	0.01	0.95
	[0.2, 0.4]	[0.2, 0.4]	0.074	0.45	0.39
	[0.1, 0.2]	[0.1, 0.2]	0.034	0.93	0.00
	[0.1. 0.2]	[0.4, 0.5]	0.038	0.02	0.90
	[0.4, 0.5]	[0.1, 0.2]	0.036	0.91	0.01

Table B. Mean time to the fixation (q > 0.9) of A_2 in the multi-locus simulation of the TP model

mode	L	c	$\bar{T}$	c. v.*
soft selection, Φ = 2 (a_S = 0.1, b_S = 0, a_U = 0.25, a_V = 0.05, b_U = b_V = 0.05)	1	-	5863	-
	5	10^{-4}	9287	0.66 ± 0.21
	5	10^{-3}	8212	0.67 ± 0.20
	5	0.01	8621	0.70 ± 0.26
	5	0.1	8162	0.65 ± 0.26
hard selection, Φ = 0.75 (a_S = 0.2, b_S = 0, a_U = 0.25, a_V = 0.1, b_U = b_V = 0.05)	1	-	6242	-
	5	10^{-4}	3776	0.34 ± 0.17
	5	10^{-3}	3228	0.32 ± 0.16
	5	0.01	3030	0.31 ± 0.20
	5	0.1	3459	0.28 ± 0.19
hard selection, Φ = 1 (a_S = 0.15, b_S = 0, a_U = 0.15, b_U =0.05, a_V =0, b_V = 0.1)	1	-	5177	-
	5	10^{-4}	4648	0.35 ± 0.15
	5	10^{-3}	4018	0.35 ± 0.17
	5	0.01	3969	0.32 ± 0.20
	5	0.1	3467	0.37 ± 0.21

*Coefficient of variation for 5 consecutive times (T_1, ..., T_5) observed in a single simulation run, averaged over replicates (± standard deviation).

All results are based on 100 replicates for each parameter set

Table C. Long-term polymorphism in multi-locus simulations of the LSA model

mode	a_S	b_S	Φ’	$\bar{H}$	P_{01}	P_{90}
neutral	0	0	-	0.0757	0.42	0.40
soft selection	0.05	0.2	0.294	0.013	1	0
	0.1	0.2	0.5	0.017	0.96	0
	0.1	0.15	0.769	0.040	0.91	0.00
	0.2	0.1	1	0.152	0.63	0.08
	0.2	0	1.25	0.116	0.31	0.41
	0.15	0.05	1.5	0.054	0.17	0.71
	0.1	0.05	2	0.023	0.22	0.73
hard selection	0.05	0.2	0.294	0.006	0.99	0
	0.1	0.2	0.5	0.013	0.97	0
	0.1	0.15	0.769	0.009	0.22	0.76
	0.2	0.1	1	0.003	0.07	0.93
	0.15	0.087	1.25	0.003	0.24	0.76
	0.15	0.05	1.5	0.005	0.06	0.92

- Each simulation ran for 50,000 time units, except for the neutral simulation that ran 500,000 time units.

- Other parameters: $\mu = 2\times10^{-5}$, $K_{L0} = 5000$, $a_L = 0.25$, $b_L = 0.1$, $e_L = 0.7$, $e_S = 0.7$, $L = 4$, $c = 0.03$.
