# Supplementary material for: Noncovarying storage effect: Balancing and positive directional selection on mutant alleles that amplify random fitness and demographic fluctuations
Source: PLoS One. 2025 Jul 22;20(7):e0328130. doi: 10.1371/journal.pone.0328130 (PMC12282919; doi:10.1371/journal.pone.0328130)
Supplement: S1 Fig — (PDF) [file pone.0328130.s004.pdf]

Supporting Information

For “Noncovarying storage effect: balancing and positive directional selection on mutant alleles that amplify random fitness and demographic fluctuations”

by Yuseob Kim, Ewha Womans University

S1 Figures

Figure A

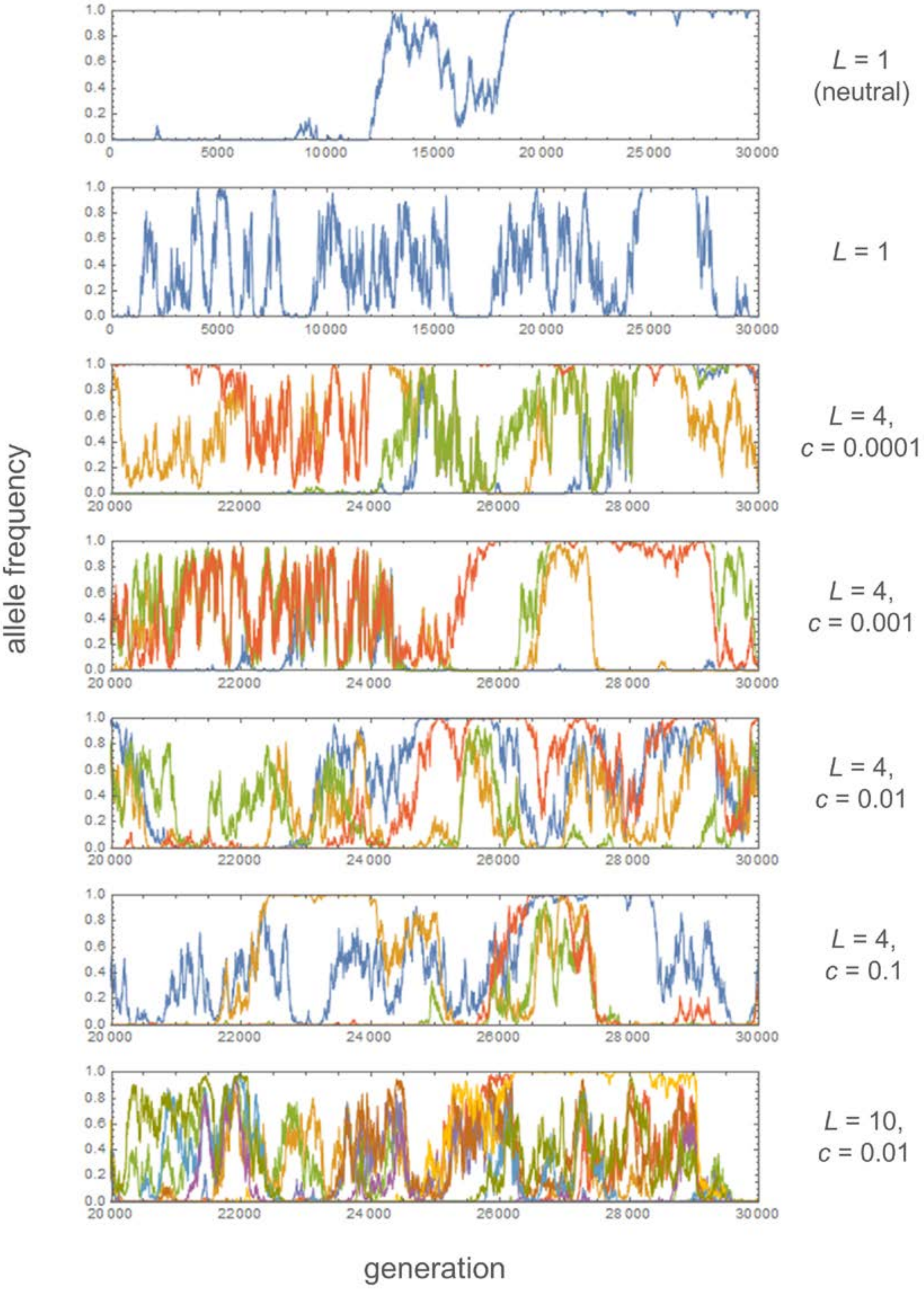

Figure A legend: Exemplary trajectories of allele (A_2) frequencies in simulations. Allele frequencies are plotted in different colors for different loci. The number of loci (L) and recombination rate (c) are shown on the right side of graphs. Other parameters: $K_{R0} = K_{F0} = 1000$, $\mu = 2\times10^{-5}$, $\Phi = 1$ ($a_S = 0.2$ [0 for neutral], $b_S = 0.1$, $a_U = 0.3$, $b_U = 0.1$, $a_V = 0.05$, $b_V = 0.1$).

Figure B

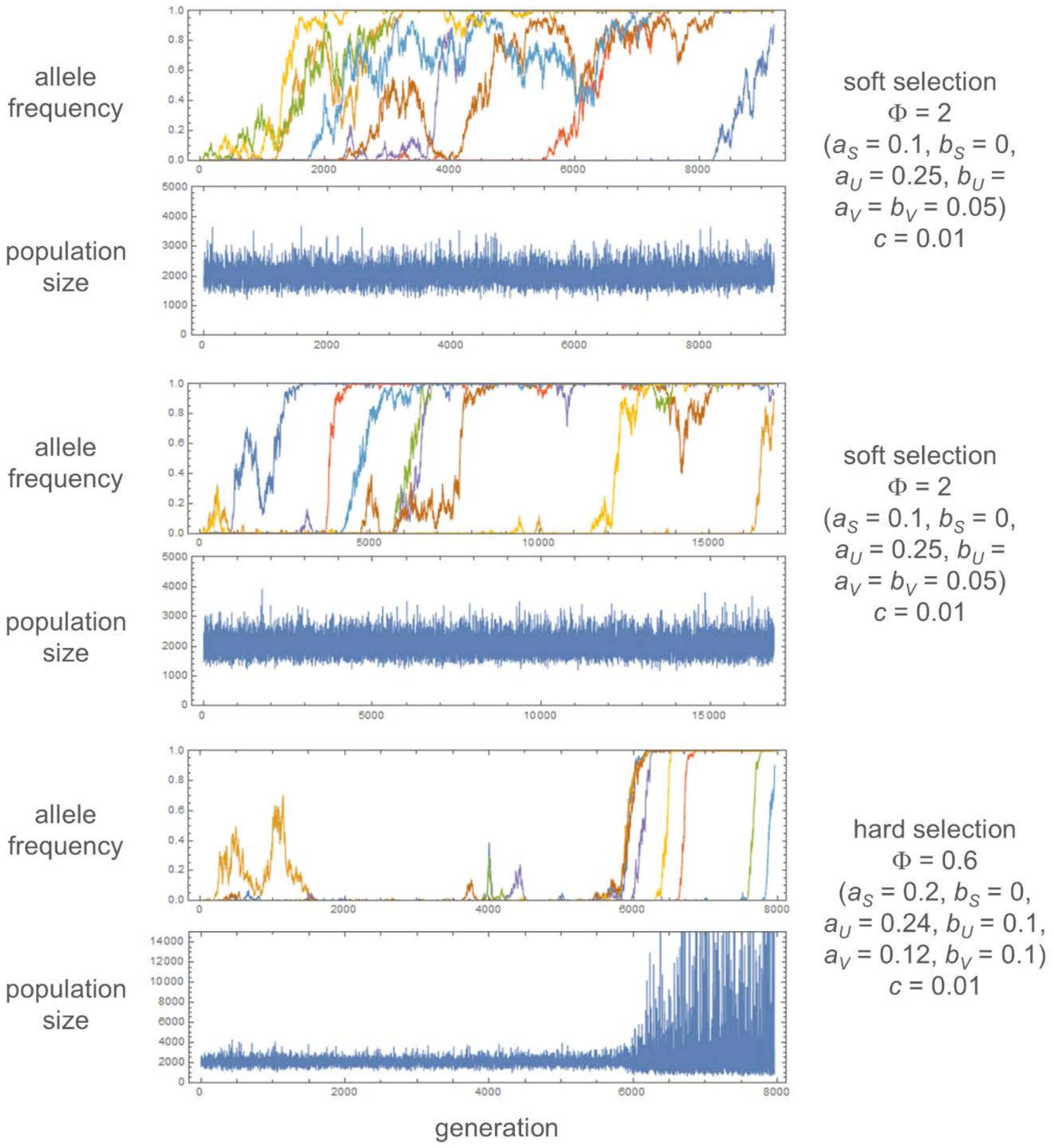
allele frequency
population size
soft selection
Φ = 2
(a_S = 0.1, b_S = 0,
a_U = 0.25, b_U =
a_V = b_V = 0.05)
c = 0.01
allele frequency
population size
soft selection
Φ = 2
(a_S = 0.1, b_S = 0,
a_U = 0.25, b_U =
a_V = b_V = 0.05)
c = 0.01
allele frequency
population size
hard selection
Φ = 0.6
(a_S = 0.2, b_S = 0,
a_U = 0.24, b_U = 0.1,
a_V = 0.12, b_V = 0.1)
c = 0.01
generation

Figure B (continued)

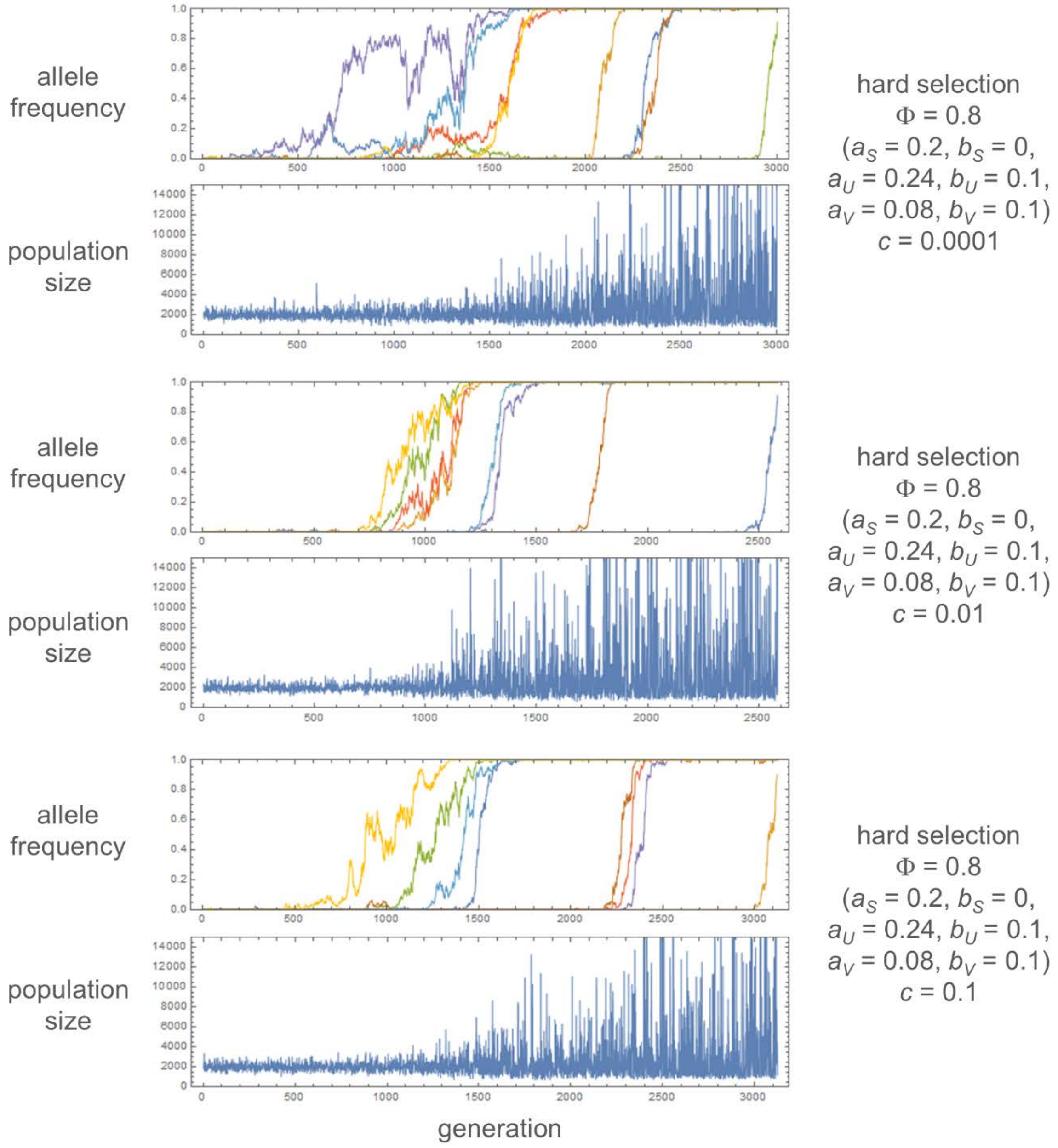

Figure B legend: Examples of simulation runs in which the mutant allele (A_2) reaches fixation sequentially at all 8 loci used in the TP model. The change of population size is shown below that of allele frequencies, plotted in different colors for different loci. Parameter values are shown on the right side of graphs. Other parameters: K_{R0} = K_{F0} = 1000, μ = 2×10^{-5}.

Figure C

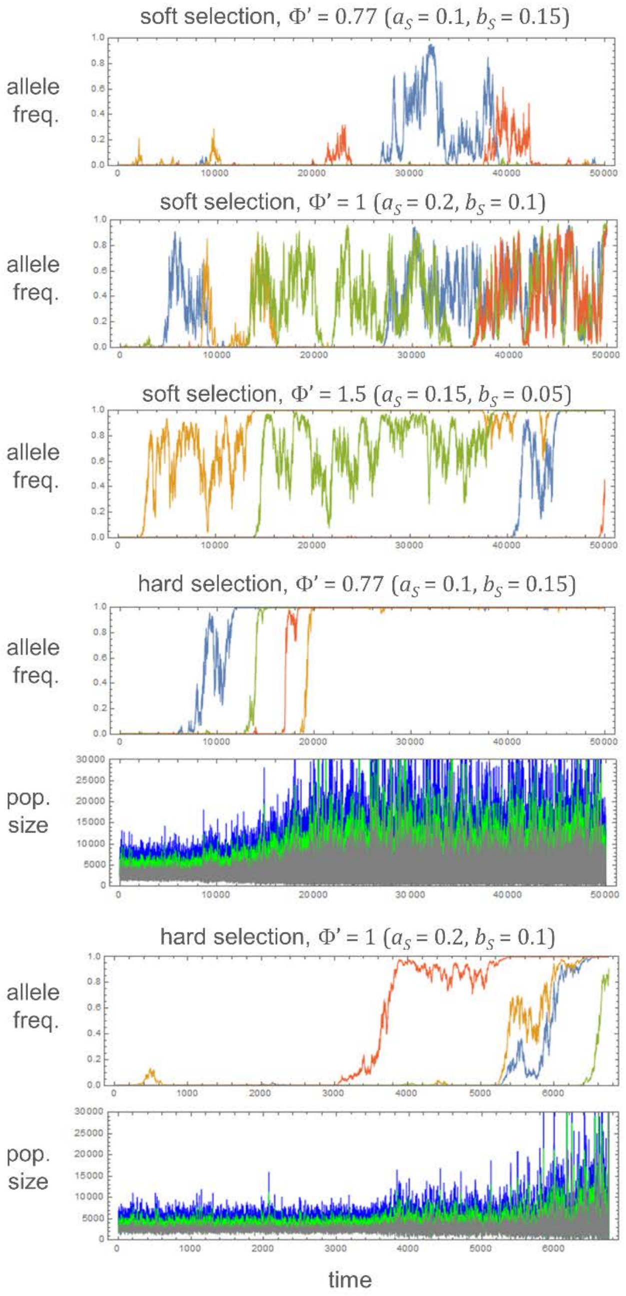

Figure C Legend: Exemplary allele frequency changes in the simulation of the LSA model. Each simulation started with a population fixed for the A_1 allele and ran for 50,000 time units, except for the case of hard selection with Φ' = 1. The A_2 allele appears by recurrent mutations at each of L = 4 loci. Allele frequencies are plotted in different colors for different loci. For hard selection, the change of population size (blue: larval, green: subadult, gray: adult) is shown below the allele frequency trajectories. Other parameters: K_{L0} = 5000, a_L = 0.25, b_L = 0.1, e_L = e_S = 0.7, μ = 2×10^{-5}, c = 0.03.

Figure D

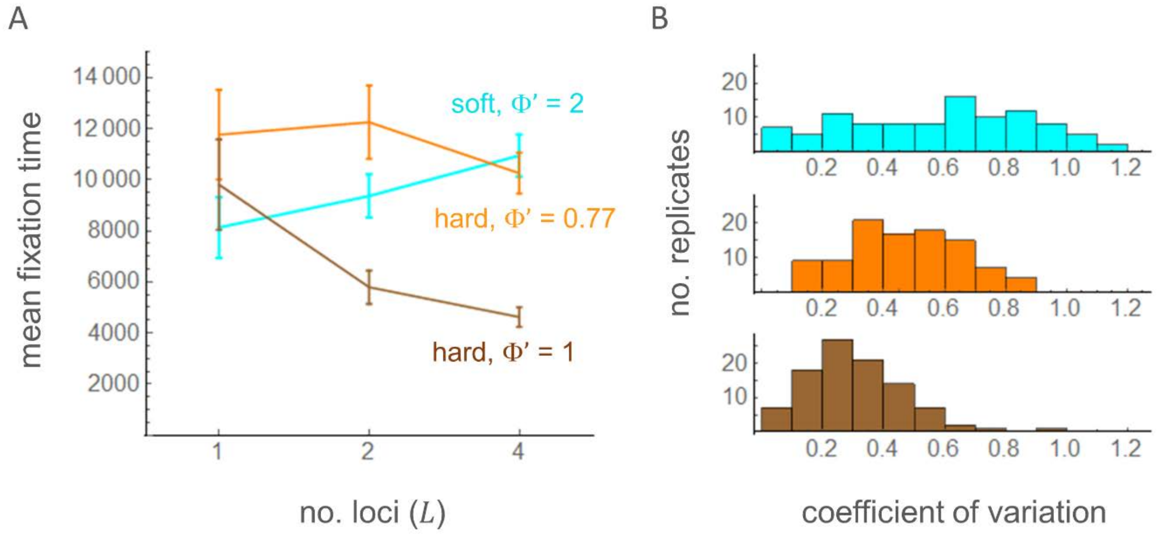

Figure D legend: Mean fixation time ($\bar{T}$) with varying number of loci (A; L = 1, 2, and 4) and the coefficient of variation in fixation times with L = 4 in the multi-locus simulation of the LSA model. Error bars show two times the standard errors. A total of 100 replicates of simulations were run under soft selection with Φ' = 2 (cyan; a_S = 0.1, b_S = 0.05, a_L = 0.25, b_L = 0.1, μ = 3×10^{-5}), hard selection with Φ' = 1 (brown; a_S = 0.2, b_S = 0.1, a_L = 0.25, b_L = 0.1, μ = 2×10^{-5}), or hard selection with Φ' = 0.77 (orange; a_S = 0.1, b_S = 0.15, a_L = 0.25, b_L = 0.1, μ = 3×10^{-5}). Other parameters are identical to those in Figure C.
