# Supplementary material for: Noncovarying storage effect: Balancing and positive directional selection on mutant alleles that amplify random fitness and demographic fluctuations
Source: PLoS One. 2025 Jul 22;20(7):e0328130. doi: 10.1371/journal.pone.0328130 (PMC12282919; doi:10.1371/journal.pone.0328130)
Supplement: Attachment — Submitted filename: GeneticsDecisionResponses.pdf [file pone.0328130.s005.pdf]

Here is the decision letter on the previous version of manuscript submitted to Genetics. Review comments by the editor and three referees are followed by my responses shown in blue. Page, line, and Figure numbers refer to those in the current manuscript.

Yuseob Kim, Ewha Womans University, Korea

November 13, 2024
MS ID#: GENETICS-2024-307536

Dear Dr. Kim:

Thank you for submitting your manuscript entitled "Noncovarying storage effect: positive selection on mutant alleles that amplify random fitness and demographic fluctuations" for publication in GENETICS. After considering the reviewers' comments we regret to inform you that it is not acceptable for publication. We would not consider a revised manuscript.

The reviewers were, unfortunately, all in agreement that the manuscript was technical and challenging to read. While I believe that the reviewers comments will offer substantial guidance to you in clarifying and streamlining the presentation, the overall evaluation is that it is too technical for GENETICS and not a substantial enough advance. In addition, the reviewers point out a few assumptions that limit its generality for this audience (e.g., no temporal autocorrelation).

Thank you again for your interest in GENETICS. We hope this action does not deter you from submitting future manuscripts to the journal.

Sincerely,
Sarah Otto
Associate Editor
GENETICS

Approved by:
Nicholas Barton
Senior Editor
GENETICS

I strongly disagree with the assessment by associate editors that the current manuscript is not a sufficient advancement over Kim (2023), for the following reasons.

- Changing the model from simple symmetrical oscillation to random fluctuation of environment alone greatly expands the biological systems to which the model applies.
- While Kim (2023) focused on the seed-bank model, this study analyzed the model of two ecological patches (the TP model) and the model of an age-structured population divided into larval, subadult, and adult stages.

- Mathematical solutions, the conditions for different evolutionary outcomes based on the covariance and variance of model parameters, on the random fluctuating environment were derived using approaches completely different from that used in Kim (2023). I believe this mathematical approach itself merits its own publication, because a similar problem in population genetics dealing with fluctuating environments might be solved by taking the similar steps. In addition, the analysis clarified the role and requirement of "refuge" that is essential in generating positive selection on rare fitness-amplifying alleles. This led to the proposal of new term "noncovarying storage effect"

- Most importantly, this study first demonstrated the positive feedback between population size fluctuation and fitness fluctuation using multi-locus simulation. The dramatic result is shown in Figure 4. I am very excited to report this result because, to my knowledge, no other studies demonstrated that evolutionary changes can contribute significantly to the amplification of demographic fluctuation. This result was only vaguely anticipated in Kim (2023), which analyzed only one-locus model.

I also disagree with the criticism that this study is too technical. Details of mathematical derivations are located in the Appendix. Readers can read main text only to understand the construction of the model and the main effects of parameters, particularly with simulation results.

Lastly, it is true that temporal autocorrelation was not explored in this study but I think the model of periodic oscillation in selective environment over multiple generations in Kim (2023) demonstrated that the noncovarying storage effect is operating well with temporal autocorrelation.

Reviewer #1 :

This work is a continuation of Kim (2023; Proc. R. Soc. B), where the author models fluctuating selection in a structured population with field vs refuge sub-populations. In this study, the author re-formulated the fitness and sub-population carrying capacities such that they fluctuates through combinations of random walks instead of cyclic oscillation (as in Kim 2023). This formulation allows easier analysis of covariances among fitness and demographic variables. It also allows the author to model soft vs hard selection, as defined in Wallace 1975, where the fitness also changes the population size through litter sizes in the latter case (modeled by letting fitness and carrying capacity partially covary). Through simulations, the author examines the distributions of allele frequencies over evolutionary times across the parameter space and shows they match the analytical approximations. They also extend the simulation framework to consider multiple linked loci and non-Wright Fisher populations with multiple life stages and overlapping generations. The simulations under these two models align with model predictions, that in addition to the storage effect that maintains polymorphisms, when fitness fluctuations covaries little with field-refuge carrying capacity differences, the fecundity-affecting fluctuating alleles are prone to fix, which would further increase the variance in litter size /amplitude of population size fluctuations.

This is very nice summary and rephrasing of this study. Thanks.

Whereas it's nice to have a model that incorporates eco-evolutionary population dynamics with pop gen,

my main issue is how the author defines directional vs balancing selection and their choice of criteria to classify them. Instead of frequency distributions after >>2Ne generations, maybe the author could consider the probability of fixation and/or time to fixation as key indicators. Examples see Cvijovic & Good et al. (2019, PNAS) and Kaushik & Jain (2021, Genetics). Even if the derivation gets too analytically challenging, distributions out of simulations are still informative. This could lend more support to the claims of directional selection for hard selection in some parameter space explored in this manuscript. (In other words, the evidence presented in the manuscript cannot yet convince me that those mutations fix faster than neutral ones under drift.)

There was a mistake of not explaining sufficiently the change of scheme in classifying simulation results (balanced, lost, fixed, neutral) after the first simulation result with $S^* = 0$ was given (by the distribution of allele frequencies at the 5000th generation). Beginning with the results with $S^* \neq 0$ (Figure 3), the evolutionary dynamics were summarized by the mean heterozygosity and the proportions of time the A_2 allele spent in the frequency interval [0, 0.1] and [0.9, 1.0] when a given simulation was run more than 100,000 generations. Visual inspection of allele frequency trajectories confirmed that this method could distinguish qualitative differences in the allele dynamics and the underlying dominant evolutionary forces.

About data availability: I had a hard time locating the correct entry using the DOI provided. I eventually found it by querying the author's name on zenodo site. Would it be more straightforward to show URL instead? (https://zenodo.org/records/12516522)

This mistake was corrected and now updated Mathematica files are available in Github.

Despite the above points, this study still warrants merits both in coupling selection and demography through fecundity, and in analyzing the conditions under which population structure becomes less capable of retaining polymorphisms due to fluctuating selection (somewhat echoing results in Cvijovic & Good et al. 2019 in unstructured populations).

Below are more specific comments:

All minor corrections and clarification of terms were made as suggested.

- Line 71: From the context, does the "size" here refer to the carrying capacity and/or litter size (instead of the effective population size)? I think this is worth clarifying at the beginning.

- Line 165: "a simple demographic model" is quite ambiguous here. Could you give a more specific definition to it? E.g. unstructured population? Constant population size?

- Line 180: the notion of soft/hard selection in the context of Wallace (1975) might conflate with soft/hard sweeps, which have been somewhat a hot topic in the pop gen community in the past two decades. The author might want to clarify this upfront earlier in the Introduction.

It is now stated that the use of terms soft and hard selection is for convenience. What they mean in this

manuscript is now described in more detail (page 9).

- Line 183: ", where W is 1 for A1..." --> ", where the fitness W is 1 for A1..." (although it's quite a common to practice to encode absolute fitness with W, it's still better to be clear when defining a variable.)
- Line 188: typo, expeced --> expected

- Line 225: I don't think δ has been properly defined yet. It could make subsequent reasoning hard to follow for some readers. The author should note that it is the fold difference between the adaptive effects of A2 in refuge vs field, and that it's a variable.

- Line 267: how are a's and b's defined here?

Definitions were added (page 12)

- Fig1:
○ Caption should also explain what the dashed lines in panel B & D are.
○ I don't think either the caption or main text properly defined P15 and P59
○ Why aren't there neutral reference lines in panel A & C? How are these distributions compared with selection with stochastic K and S=0? How about when there's only drift but no mutations?

There is no mutations and genetic drift in panel A & C. However, it is still stochastic because the parameters of fitness and carrying capacities are drawn from distributions each generation. Given these parameters, the change of allele frequencies are deterministic.

○ Is a duration of 2500--5000 generations (or 6e5 for fig2) really realistic, especially when the reference population size (K_R0 + K_F0) is only 2000? Considering that time-averaged Ne is the harmonic mean of N(t), the effective pop size would be even smaller. In other words, here the author is simulating over a duration that is several coalescent units long...

In both ways of classifying the dominant evolutionary forces, a sufficient number of generations in simulation is needed to distinguish balancing, negative, and positive selection from neutral genetic drift.

- Fig2:
○ What happened to the (x,y)=(0.3, 2.5) point?

Now positive selection is inferred only if $\overline{H} < 0.05$ and $P_{90} > 0.7$ (page 14, line 330). This increased the number of cases, in addition to (x,y)=(0.3, 2.5), that are classified as neutral. Explanation for results that appear neutral was added (page 14, lines 337-343).

○ I highly suspect the majority of the caption belongs to the main text.
- Table 1:
○ Instead of (or in addition to) showing the values of a & b parameters, it might be more intuitive to show the expectations of key variables (S, S*, U, V).

It was explained earlier that the expectations of those variables are all zero.

○ The author should also consider cases where recombination distance $c=1$ (i.e. linkage equilibrium).

Large scale simulations on larger parameter space are now performed for the next study investigating the feasibility of noncovarying storage effect in explaining the empirical observation of genome-wide oscillatory polymorphism.

○ 1e5 generations is way too long for a population with a small time-averaged Ne...
○ As mentioned earlier, I don't think $\bar{H}$, P_15, and P_59 (at >>10Ne generations) are good indicators for modes of selection, though one can argue that any polymorphism lasting ~10+ coalescent unit time is almost guaranteed to be under long-term balancing selection.
○ Instead of a table, the results might be better displayed by a barplot to show the values of H, P_15 and P_59 across varying settings. It's also worth running a few more replicates for each scenario so one can test for statistical significance.

Again these concerns stem from my failure of explaining the method of classifying evolutionary forces after Figure 2 – not by the allele frequency distributions at a fixed time point but averaging *H* over the entire duration of a simulation run.

Regarding the multi-locus model, on the one hand, it is commendable for the author to extend the framework to cases where multiple loci could affect fecundity. I appreciate the author's effort to address them. On the other hand, my mind kept going to the space of polygenic stabilizing selection (see Simons et al. 2018), seeing the litter size as a quantitative trait and selection operates on traits. I might be subconsciously compare the two model settings while reading, which could potentially bias my judgement on this front.

Investigation on more realistic model for multilocus genotype-phenotype-fitness relationship is being planned for the follow-up studies.

- Line 357: missing space in "selection can"
- Line 504: "... is not so weak or strong" <--- this reads very ambiguous. Might need rephrasing.

Reviewer #2 :

As the title indicates, this is a highly technical theory-based manuscript that addresses the consequences of alleles whose stochastically varying effects alter both relative and absolute reproductive success. The author clearly realizes that Peter Chesson introduced the term "storage effect" to describe fluctuation-based mechanisms of species coexistence. (For a detailed discussion, see pp. 351-353 of Chesson, 2000. Ann. Rev. Ecol. Syst. 31:343-366). However, the author makes no serious attempt to relate his population genetic analyses to Chesson's sophisticated body of mathematical theory that has been highly influential among both theoretical and empirical ecologists. Instead, Kim's point of reference is a newer theoretical literature on a "storage effect" in population genetics that he assumes his readers know. I doubt this is true for many readers of Genetics and the confusing Abstract provides little motivation to dig into the details of that literature.

In the previous version I stated in the Introduction that “The storage effect was first theorized in

community ecology as a mechanism for promoting species coexistence (Chesson & Warner 1981; Barabás *et al.* 2018).” If that is not enough, I added more details in the current version (page 5): “The storage effect was first theorized in community ecology, shown to arise not only with partial protection from fluctuation provided by a refuge but with general covariance between environment and competition, and is now one of the most important ecological theories for explaining the stable coexistence of species (Chesson & Warner 1981; Chesson 1994; Barabás *et al.* 2018).”

I found the Abstract essentially impossible to understand. It seems that a central conclusion of the analytical and numerical analyses concerns selection favoring "fitness-amplifying mutations" that also amplify "population size oscillations." Furthermore, "... this novel evolutionary force is generated when the size of the subpopulation subject to weak selection does not covary with the rest of the population." After reading this arcane summary, it was hard to be motivated to understand the details leading up to these conclusions. Without more detailed biological examples that motivate the analyses and a clearer presentation of the underlying models, these analyses seem far more appropriate for a theory-focused journal such as Theoretical Population Biology or Journal of Theoretical Biology. Surely, as indicated by my comments on the first few pages, a massive re-write is required to make this manuscript digestible for any audience.

I don’t think it is fair to criticize the lack of detailed information in the Abstract that is limited to only 250 words. One cannot judge if the article is only for theory oriented readers just by reading its abstract.

I made the comments below while I read the manuscript. They refer to specific lines.
34-36. Do any data indicate that populations that fluctuate more tend to be more polymorphic? My guess is that this is an implication of the theory. Overall, I see no attempt to relate the theory to empirically testable predictions. An obvious difficulty is that if populations crash, drift will purge variation.
46-47. Do we need a genetic explanation for fluctuating populations? How might a genetic explanation be distinguished from effects associated with climate variation or interacting species?
61-64. This summary of previous results is misleading. Selection acts to decrease variance only among alleles with comparable mean fitness effects. Specific models and results should be cited against which the new results can be compared.
97-113. This jargon-rich summery of previous results is unlikely to be accessible to many Genetics readers.
123-143. Overall, this manuscript is very difficult to follow. Weirdly, the author introduces the acronym LSA for one of his models before he defines the model.
TP model. After discussing some plausible biology in the introduction, the author presents a range of model assumptions -- e.g., hard versus soft selection, Poisson offspring numbers -- in what seems a stream of consciousness. Why conflate so many effects? If you have found an interesting/important new theoretical prediction, you should be able to explain it in the simplest possible context, then discuss elaborations if they seem useful.
209. It is typical of the chaotic presentation that the Poisson offspring numbers are introduced on lines 182-183 "to accommodate genetic drift," yet when the analysis starts, drift is ignored without mentioning Poisson.
215. I stopped reading at this point. In a paper aimed at theoreticians, surely you must clearly explain the underlying dynamics of the key equation, without referring to an Appendix!

It is clear from the last comment that this reviewer did not make enough effort in understanding the major results and conclusions of this study that two other reviewers had little problem in understanding.

Reviewer #3 :

Summary
I have reviewed the manuscript "Noncovarying storage effect: positive selection on mutant alleles that amplify random fitness and demographic fluctuations" by Yuseob Kim. This manuscript is a follow-up study of the author's 2023 paper "Partial protection from fluctuating selection leads to evolution towards wider population size fluctuation and a novel mechanism of balancing selection". Compared to the previous paper, the author is here exploring two additional somewhat more ecologically realistic models. The author provides nice analytical results for the invasion of alleles and for maintenance of polymorphism and supports them by simulations for one or multiple loci.

Although the paper is in terms of language well-written and the topic is very interesting, I still had quite a hard time understanding the model and results, at least at first reading. I think in many places the biological interpretation of parameters and results can be improved to make the paper more accessible and increase its impact. I provide some specific suggestions below.

I appreciate your positive assessment on this manuscript. I have rewritten many parts of the manuscript so that the construction of the models can be easier to be understood, as listed below.

Major comments
- At the beginning of the model description: What are the variables you are keeping track of? Having an overview would be helpful to orient readers.

- Please clearly describe the order of events in the life cycle early on in the model description. It took me a really long time to figure out how the life cycle works because the different processes were described not in the order in which they happen in the life cycle, I think. A flowchart might also help.

- On a related note, where does the new time step start? I also found it a bit confusing that the time index is often omitted.

Now a figure was added (Figure 1) to illustrate the overall structure of population, the flow of events over time, and the major variable to keep track of in the TP and LSA models. I also clarified texts regarding when the cycle of events starts and the order of events.

- Please give intuitive names to key quantities like phi, rho, delta etc. and use these names in addition to the Greek letters when talking about them. I think it will reduce the cognitive load for the readers.

- A relevant, but not cited paper looking at the effect of environmental fluctuations in an eco-evolutionary model with both allele frequencies and population sizes changing is Lande (2008) Adaptive topography of fluctuating selection in a Mendelian population. https://doi.org/10.1111/j.1420-9101.2008.01533.x It would be nice to discuss how this model and its results differs from yours.

- In my experience, soft selection and hard selection are notoriously confusing and people use these terms in different ways. Could you explain why you chose the specific expressions for soft and hard selection in L183 and L190?

It is now stated that the use of terms soft and hard selection is for convenience. What they mean in this manuscript is now described in more detail (page 9).

- L202-203: Please provide a clear biological motivation for these assumptions. For example, I could not understand why you looked here at the difference between U and V.

As explained in the next section, positive selection on the rare allele (the elevation of the geometric mean fitness of the A_2 allele in the whole population above 1) is possible when the size of the field (~exp[U]) does not covary with the size of refuge (~exp[V]). If U and V vary in correlated manner, the effect of demographic fluctuation increasing the geometric mean disappears. Therefore, what matters is the covariance of S with U-V. This heuristic argument for the noncovarying storage effect is given on page 22 (middle paragraph).

- L673-675: This is a small-variance approximation, right? Could you discuss somewhere in the main text in what settings this approximation and the resulting criteria would work well and under what conditions the approximation would break down?

It is true that the derivation is based on the quadratic approximation of Δ_2 and this approximation is better with smaller deviation of variables (U, U', V, V', S, S^*) from 0. Therefore, the solution will be more accurate with smaller variances. However, it is not easy to analyze mathematically how small the variances should be. A practical way would be to compare the analytic approximations to simulation results, which could not be done over the entire parameter space.

- I checked all the derivations in Appendix A and they all appear correct, with two doubts/questions that you can probably easily explain

I am very delighted to hear that you find most of my derivations correct.

1. I had to divide by the respective terms now in the denominators of L683 and L694 to obtain these inequalities. It is guaranteed that these terms are always positive so that the direction of the inequality is preserved? Why or under what conditions?

Thanks for pointing this out. Now I added "assuming $(\mathrm{Var}[S]+\rho^2\mathrm{Var}[S^*])/\rho > -2\mathrm{Cov}[S,S^*]$" (line 719)

2. In L703, where do the factors 1/(1+S_t) and 1/(1+S_t*) in the first expression after the = sign come from?
Additionally, for the invasion of the A_1 allele in L486-496, I was able to figure it out, but it might be helpful to provide some additional steps in the derivation similar to what you did for the invasion of the A_2 allele.

I explained that it is because the relative fitness of A_1 is the inverse of those for A_2 (line 723).

Minor comments:

- Data availability: I had some trouble finding the scripts on zenodo as there seems to be an error in the doi provided. I found some code for a manuscript with a different title and a similar doi, which is probably the code intended for this manuscript. Please clarify. If you used Mathematica for some of the analytical calculations, it might also be nice to provide that Mathematica notebook since some of the calculations are quite tedious to do by hand.

It was corrected. Now it is available from Github.

- Does the title fit well? The first part of the title suggests that it is about a mechanisms for maintenance of diversity, the second part that it is about positive selection. Could the title reflect that it is about both?

The title was changed.

- Please mention that there is no temporal autocorrelation in your model.
- L29: size of the rest of the population?
- The article summary (L40-47) could equally well apply to the 2023 Proceedings B paper, I think. Would it make sense to make it a bit more specific for the current paper?
- L41: What is new, the eco-evolutionary dynamics or their description?
- L53-58: The logical structure of this argument is a bit unclear because you talk about demographic fluctuations at the beginning and also at the end, but in a different way.
- L133, L136: Please introduce here what TP and LSA stand for to make it easier to memorize them.
- L166-167: Here you call K_F and K_R the carrying capacities, but for the hard selection model below you sometimes refer to K_F(1+S) etc. as carrying capacity, which is slightly confusing.
- L173: remains, migrates
- L174: "individual" missing after "each"
- L175: at the beginning of what?
- L176-177: What is the expectation over? Different outcomes of the random migration process?
- L182: Is it really a hard constraint if individuals produce Poisson-distributed offspring independently? Is just the expectation constrained?
- L183: progenies -> progeny (I think)
- L188: expeced -> expected
- L209: "the" missing before "derivation"
- L209: What is the biological interpretation of this fixed scheme of migration?
- L211-212: Is this a special case of the above model or an additional assumption?
- L216: "it" not needed
- L220-221: commas not needed
- L232: Not at all? Or not too strongly?
- L266: "the" at the beginning of the line -> "a"
- L275: Why initially?
- L294: "under the same condition" confused me here. Could it be removed? Also, I would replace "allele" by "locus".
- A couple of different comments on Fig. 1: Please explain in the figure caption what the dashed line represents. Also, could you motivate somewhere why you chose initial frequency 0.5 for these simulations? And do missing values indicate a zero proportion? Would it be possible to increase the number of replicates to get a somewhat less noisy picture?
- L307: balacing -> balancing
- L311-313: Where can we see this?

- L338: simulation -> simulations
- L357: space missing in selectioncan
- L365-366: Where can we see this? I somehow could not figure out which cells of the table to look at.
- L411: is assumed -> are assumed
- L412: What is a unit? "the" missing before "larval population"
- L414: subadut -> subadult
- L415: "the" missing at the very beginning
- L418: "the" missing before A_1
- L424-426: Could you motivate why you included two "refuge" stages? Would you expect the results to be different in a model with just one refuge stage?
- L436: Please provide a derivation or argument somewhere.
- L437: What value?
- L465: I think the "in" after "and/or" needs to be removed.
- L467-469: Under soft or hard selection?
- L487-489: But it could still be called non-covarying with respect to the selection pressures, right?
- L532-534: I could not understand this sentence.
- L676: E[Delta_2] missing in the expression of the Taylor coefficient.
- L714: "The" missing at the beginning of the new sentence.
- L730-732: Please explain the new dot notation.
- L733-734: How is it avoided that the number of migrants is larger than the size of the source population?
- L740-742: Why do mutation and recombination only occur in subadults and adults? This seems biologically a strange assumption.

I made corrections and clarifications to all of the minor review comments. Thanks for finding out errors and suggestions.
