# Supplementary material for: Noncovarying storage effect: Balancing and positive directional selection on mutant alleles that amplify random fitness and demographic fluctuations
Source: PLoS One. 2025 Jul 22;20(7):e0328130. doi: 10.1371/journal.pone.0328130 (PMC12282919; doi:10.1371/journal.pone.0328130)
Supplement: Attachment — Submitted filename: POneResponsToReview.pdf [file pone.0328130.s007.pdf]

Response to reviewer comments (PONE-D-24-54939)

I revised the manuscript following the comments provided by two reviewers. Below each review comment (in italic) is followed by my response. Line numbers are those of revised manuscript.

Yuseob Kim
Ewha Womans University

Editor's comment:
While both reviewers agree that there is some merit in the article reviewer#1 suggests a change on the pitch of the article, while reviewer #2 founds the current version unsuitable for publication. As I found the reviewers comments constructive, I would like to ask you to revise your manuscript. However, this will be a major revision, with substantial changes required. Particularly the language needs to be adjusted for better readability and the mathematics must be sound. As reviewer #2 suggests rejection, the manuscript must be a substantially improvement, with a clear presentation and rigorous mathematics. Reviewer #2 mentions that some frustration is accompanying the manuscript. However, I believe that both reviewers make constructive comments that will guide a substantial revision, which should be regarded an opportunity rather than a cause for more frustration.

>> I greatly appreciate your decision to allow me to revise and resubmit the manuscript. I revised manuscripts to address all comments by both reviewers. I tried to revise texts in Abstract, Introduction, and Discussion to improve readability and remove confusions.

Reviewers' comments:

Reviewer #1: The author studies the maintenance of variation under fluctuating selection. Using two different population models, two-patch model and larval-subadult-adult model with hard and soft selection. The work shown in this manuscript is a generalization of previous results of the author. While the analyses are complete and convincing, the reviewer is concerned about the pitch of the manuscript.My understanding is that variation is maintained only under a set of very restricted conditions, hence the focus should be on the difficulty to maintain variation under fluctuating selection, rather than portraying it as a realistic scenario. As written, the reviewer feels that the study may be cited for evidence that variation is maintained under fluctuating selection, although most results show the opposite. It may also help to make more effort to discuss how biologically reasonable the permissive parameter space is. In other words, how likely is it that these conditions are met in natural populations?

>> This aspect of the result – in larger parameter space the mutant alleles under fitness fluctuations are positively selected to fixation rather than maintained in polymorphism – was indeed not discussed in depth. I agree that it appears to suggest that the new version of storage effect affected by demographic fluctuations, the noncovarying storage effect (NSE), fails to generate balancing selection. Now I state "One may therefore regard the population size oscillation as a factor that destroys the potential of storage effect to generate balanced polymorphism." (l. 111)

However, whether there will be more or less balanced polymorphism in nature due to the NSE depends on what kinds of mutants with their own patterns of fluctuating fitness will be available for the NSE to act on. As pointed out in Kim (2023) and this study, the previous models of the storage effect (without population size fluctuation) can maintain polymorphism only when mutants have identical geometric mean fitness to the wild-type allele (thus GMF mutants), which is possible only if its arithmetic mean fitness is greater than the wild-type. NSE leads to either loss or fixation of GMF mutants. However, the NSE produces balanced polymorphism due to AMF mutations that confer the same arithmetic mean fitness as the wild-type. Therefore, given the assumption that AMF mutations are more likely to occur than GMF mutations, which I believe is probably true, population size fluctuation, thus NSE, makes it more plausible to have balanced polymorphism in the genome than the previous theories of the storage effect predicted. I therefore argue in the Introduction that "However, this population size oscillation led to balancing selection on a different type of mutants — alleles conferring smaller geometric mean but identical arithmetic mean fitness compared to the wild-type. Since such AMF (arithmetic-mean-preserving fitness fluctuation) mutations, which are deleterious in a simple population (without a refuge), are probably more likely to occur in the genome than the GMF mutations, this version of storage effect modified by demographic fluctuation might be equally important or even more important in generating balanced polymorphism in nature." (l.113-119).

Furthermore, even in the parameter space in which AMF mutants are not maintained as polymorphism but reach fixation, namely the cases of stronger demographic fluctuation relative to fitness fluctuation, there can be mutants whose fluctuation in fitness is further different from the AMF that is positively selected only when they are in low frequency so that they are maintained as polymorphism. Those mutations are likely to have net arithmetic mean fitness LOWER than that of an AMF allele, which even more plausible to occur in the genome. For this reason, we may not say that balanced polymorphism is less likely under fluctuating selection due to the NSE. This argument, using the heuristic explanation for the NSE, is given in the Discussion: "Because fixation rather than balanced polymorphism occurs in a wider parameter space, one may argue that the overall effect of NSE (i.e. the modification of the storage effect due to population size fluctuation) is to make balancing selection less likely to occur. However, given the possible patterns of mutant alleles' fitness fluctuations in nature beyond those considered so far, the NSE may be a force of increasing, rather than decreasing, the number of loci in which oscillatory polymorphism occurs. First, the NSE causes all GMF mutants to be either lost or fixed in the population, thus no balanced polymorphism (Kim 2023), but allows AMF mutants to be maintained as balanced polymorphism. As one may expect more AMF mutations than GMF mutations to occur in the genome, since more beneficial mutations (in net effect) are considered less likely in general, the number of loci under balancing selection may increase due to the NSE. This also suggests that for a given demographic fluctuation, mutations of certain fluctuation in

fitness are "picked up" to be maintained as polymorphism. In the heuristic argument above, the absolute fitness of the A_2 allele in the field is given by $W_{2F} = D_F w_2$, where D_F is its absolute fitness given by change in the population size of the field alone and w_2 is the relative fitness of A_2 in the field (e.g., in the TP model, $D_F = \frac{K_F^{(t)}}{\tilde{N}_F^{(t)}}$ and $w_2 = 1 + S_t$). It is a limited range of fluctuation in W_{2F} that produce polymorphism, as suggested in the above model with AMF mutations where a larger fluctuation in population size (thus in D_F) allows polymorphism only when the mutant alleles have a larger fluctuation in fitness relative to the wild-type (so that $\mathrm{Var}[S]$ matches $\mathrm{Cov}[S, \log[K_F/K_R]]$), which makes the arithmetic mean of W_{2F} decline. It might be possible that for a strong demographic fluctuation under which an AMF mutant is driven to fixation, a mutant with a new pattern of fitness fluctuation not considered in this study so far, possibly with even lower arithmetic mean than AMF mutants (thus < 1), might be picked up by the NSE into balanced polymorphism." (l. 574-596).

Reviewer #2:
The present article spans the fields of population genetics and ecology and, thus, should be considered interdisciplinary. The file includes comments of previous reviewers and the reply of the author. It seems that the reviewers as well as the author were quite frustrated. I do share the sentiments of the author: Like the author, I disagree "with the assessment ... that the current manuscript is not a sufficient advancement over Kim (2023)", for the reasons given by the author. I also do not think the article is too mathematical or technical and should rather be submitted to eg the journal "Theoretical Population Biology". However, I think that the present version is far from publishable. In this respect, I agree with some of the reviewers and share their frustration.

>> Thanks to your critical assessment, I could find problems in texts that were clarified and improved in presentation, as described below.

As this is an interdisciplinary study, I should maybe specify my background: I have published in population genetics, both articles that were data-heavy as well as theory-heavy. I should have no problems following the math in the present article. I have worked and published in various fields as a statistical consultant and co-written articles in those fields. I am therefore very aware of the difficulties working in interdisciplinary contexts and, especially, how much care and thinking must be invested in appropriately identifying the relevant readers, in choosing the right language, and in finding the correct terms and definitions that are acceptable in all relevant fields. It is also important to be very clear and precise, because otherwise readers of one or the other field may be lost completely. An example for an article that achieves this feat admirably is that of Bruce Wallace (1975), cited in the present article.

Unfortunately, the present article is very confusing. Like one of the earlier reviewers, I found the abstract already problematic; eg: "Recent study found that, under pre-existing oscillation in population size, the mutant is positively selected to fixation if its fitness change correlates with the rate of population growth, which further amplifies population size oscillation." This sentence should likely be split into three or more sentences. For a population geneticist the

fitness of a mutant compared to the wild-type (allele, since haploid individuals are assumed throughout) would make sense. What then does the "fitness change" of a mutant allele mean? And why should that correlate with the population size? An oscillating population size should not grow (on average); so why "the rate of population growth"? Hence, already the abstract confused me.

>> I extensively rewrote the Abstract, removing or breaking down the sentences you pointed out. Please find the track-change version of new manuscript.

The introduction did not help either, so I hoped for some clarity when the mathematical models were described. But here I was also left confused:
Fig 1. should provide an illustration for the model. I found the graphs reasonable, but the legend problematic, eg: "Reproduction, followed immediately by mutation and reproduction,..." Here the second "reproduction" makes no sense to me.

>> It was a mistake: reproduction (x) → recombination (o)

Before the passage starting at l202, populations sizes in the two patches, field F and refuge R, were described and denoted with symbols $N_F^{(t)}$ and $N_R^{(t)}$. Obviously, these are both finite. But consider: "The model so far described changes in the number of individuals without genetic drift. When a finite population size is assumed, as in simulation below, each parent in both subpopulations is modeled to produce a Poisson number of offspring with the mean specified above, which approximates genetic drift in the Wright-Fisher model." As genetic drift is zero only if the (effective) population size is infinite, the first sentence makes no sense, given that just before the finite population sizes $N_F^{(t)}$ and $N_R^{(t)}$ were described and effective population sizes are usually much smaller than census population sizes. Furthermore, the Wright-Fisher (WF) model usually considers diploid individuals; without selection a binomial distribution with mean offspring number of 2 per individual is assumed implicitly. If a haploid version of the Wright-Fisher model is assumed (as would be appropriate for the assumptions of the author), the offspring distribution per individual is again binomial with mean one. For large population sizes, the binomial distribution could be approximated by a Poisson distribution. In both cases, the strength of drift is inversely proportional the (effective) population size. In any case, the statement that "a Poisson number of offspring" approximates "genetic drift in the Wright-Fisher model" is at least confusing, if not simply wrong.

>> Now I made it clear that the model assumes finite population sizes but in the derivation of analytic approximations the changes due to genetic drift is ignored. In addition, I state that reproducing a Poisson number of offspring is not to approximate the Wright Fisher model but to set the variance of offspring number similar to that in the haploid version of the W-F model: "The model so far described only the expected changes in the numbers of A_1- and A_2-carrying individuals. However, as reproduction in a finite-sized population was implicitly assumed, how genetic drift occurs need to be specified for stochastic simulation. To set the

variance of offspring number similar to that in the haploid version of Wright-Fisher model, each parent in both subpopulations is modeled to produce a Poisson number of offspring with the mean specified above." (l. 214-220). "For convenience in the derivation, only the expected changes in the numbers of A_1- and A_2-carrying individuals will be tracked while ignoring genetic drift." (l. 237-239)
"

Close to the "heart" of the model is the stochastic variation of the key parameters S_t, S_t^*, U_t and V_t. We are informed that the expectations of all these variables are 0, and that the variances of S_t and S_t^* are positive (in the paragraph starting at l158). But we are not informed about the distribution of these two variables. On the other hand, we are informed that U_t and V_t are drawn from a joint normal distribution (paragraph starting with l207) without information on their variance-covariance matrix. Immediately, below an inequality relation of the covariances is given: $\Cov(S_t,U_t-V_t) > \Cov(S_t^*,U_t-V_t) $. Should we deduce from this that all four variables are jointly normally distributed with zero means and some arbitrary Var-Cov matrix, as long as the inequality is fulfilled? Actually in formula 2, $\Cov(S,S*)$ appears as a term. So: maybe yes?!?

>> Now, readers are informed that the specific forms of distribution is needed for simulation but not for deriving analytic solutions: "The fitness of an individual carrying A_2 relative to A_1 is given by $1 + S_t$ in the field and $1 + S_t^*$ in the refuge, where S_t and S_t^* are drawn each generation from a joint distribution with $\mathrm{E}[S_t] = \mathrm{E}[S_t^*] = 0$. While the specific form of the distribution is needed for simulation (below), for deriving analytic solution in the next section only the mean, variance, and covariance of the distribution will be needed. " (l. 174-178). Then, it is made clearer that four variables U_t, V_t, S_t and S_t^*, are all sampled together from a joint probability distribution. "More specifically, fluctuations occur such that $K_F^{(t)} = K_{F0}\mathrm{e}^{U_t}$ and $K_R^{(t)} = K_{R0}\mathrm{e}^{V_t}$ where U_t and V_t, together with S_t and S_t^*, are drawn each generation from a joint normal distribution satisfying $\mathrm{E}[U_t] = \mathrm{E}[V_t] = \mathrm{E}[S_t] = \mathrm{E}[S_t^*] = 0$. … Various assumptions can be made regarding the variance-covariance relationship among S_t, S_t^*, U_t, and V_t." (l. 224-229)

There is a further problem with S_t and S_t^*: Should S_t be less than 1, $1- S_t$ is negative and therefore the carrying capacity would also become negative (see the formulas in Appendix A), which is non-sensical, but if the two parameters are taken from a normal distribution this would be possible as the support of the normal distribution is from minus to plus infinity. The solutions would be the same as for the variables U_t and V_t: assume a log-normal distribution for S_t and S_t^* and then to use e^{S_t} instead of $1-S_t$ (the former approximates the latter anyway as long as $S-t$ is small). As the problem of negative carrying capacities may have messed up the simulations the author might have assumed that anyway. If the mathematics would have been described with the necessary rigor, including the description of the support of the random variables, this would have become obvious right away.

>> This problem was indeed recognized in early trials of simulations. The distribution of S_t or S_t^* is truncated at the lower bound -0.999 so that the fitness cannot go negative. This was mistakenly not stated in the previous version but now stated in the revised manuscript. (l. 303-304.)

The analytical results presented in Appendix A are deterministic in the allele frequencies. This fact should be pointed out in the main text. Note that this assumption leads to the paradoxical situation that a fluctuating population size is assumed to be infinitely large (such that the drift can be assumed to be zero). This problem (that I already stumbled on above) should be discussed.---In l333ff, the author actually shows that the simulations can reproduce the key predictions of the deterministic analysis. Given the assumptions, this should break down if the population sizes are very small and drift correspondingly large. Indeed, this seems to be confirmed, as the results for small population sizes are indistinguishable from neutrality. Hence, the analytical and simulation results seem to agree to the expected degree, which is reassuring.

>> Again, I made it clearer that a finite population is assumed (l. 216) and that "only the expected changes in the numbers of A_1- and A_2-carrying individuals will be tracked while ignoring genetic drift." (l. 237-239). In simulations, I made it clear that genetic drift is added to the previous step of simulation in which only deterministic allele frequency changes are tracked (l. 318). Then I mention that the results are consistent with genetic drift weakening the effect of selection (l. 327).

Generally, this impression of reassuring solidity hidden beneath a sloppy verbal description held for much of the results section; the discussion also seems generally OK. In summary, the article is far from readable. At least this reader with a popgen, math, and interdisciplinary background could not follow key parts in the abstract, the introduction and the model sections. Nevertheless, the analytic and simulation results are interesting, agree sufficiently to appear solid and seem interesting enough for eventual publication. The discussion also contained some useful further information.

>> I hope the above and other changes made in revision have improved the readability of the manuscript.

l193: "First, under soft selection, very strong density regulation occurs so that the final size of the field is constrained to $ _ $." The strength of density regulation under soft selection depends on the absolute number of individuals before density regulation. If the latter number is below the maximal capacity, no individuals would need to die or be excluded from reproduction. As a surplus of offspring is a prerequisite for evolution, this case may be considered degenerate, but at least possible.

>> Now I removed “very strong density regulation” that causes confusion. I think the earlier explanation made for soft selection gave a sufficient information: “First, under soft selection, the final size of the field is constrained to $K_F^{(t)}$.” (l. 206)

In the paragraph starting at l207: "The oscillation of carrying capacities is therefore symmetrical in the log scale, which is generally predicted in demographic models and observed in nature..." I guess that the symmetry is assumed (not predicted) in theoretical demographic models. And I consider it impossible to "observe" this in nature, although (most) data may be "consistent with" the assumption.

>> It indeed appears that the estimated population density, as described in Myers (1988), is compatible with symmetry in the log scale.

Why is it assumed that subadults are reproducing? I would define adults as individuals that have reached reproductive age, while subadults are below that. Maybe "young adults" and "old adults" would make more sense here. This model seems similar to that presented by Cenik and Wakeley (2010) PLoS One 5(9):e13019. (But those authors only consider the popgen part, not the ecological part or the feedback between the two as the present author. This shows again that the basic idea of the author is indeed interesting and worthwhile to follow up.)

>> Terms subadult and adult indeed mean young and old among reproducing individuals. Now it is made clearer (l. 152-153, 450-451). Have two age classes among reproducing adults is essential for the storage effect, where the old adults act as a storage of mutants not immediately removed under an unfavorable environment in the immediately preceding round of selection (which affect the subadult stage).
