# Supplementary material for: Noncovarying storage effect: Balancing and positive directional selection on mutant alleles that amplify random fitness and demographic fluctuations
Source: PLoS One. 2025 Jul 22;20(7):e0328130. doi: 10.1371/journal.pone.0328130 (PMC12282919; doi:10.1371/journal.pone.0328130)
Supplement: Attachment — Submitted filename: PONE-D-24-54939_R1_reviewer.pdf [file pone.0328130.s006.pdf]

PLOS ONE

Noncovarying storage effect: balancing and positive directional selection on mutant alleles that amplify random fitness and demographic fluctuations

--Manuscript Draft--

**Manuscript Number:**	PONE-D-24-54939R1
**Article Type:**	Research Article
**Full Title:**	Noncovarying storage effect: balancing and positive directional selection on mutant alleles that amplify random fitness and demographic fluctuations
**Short Title:**	Positive selection on fitness-amplifying mutants by the noncovarying storage effect
**Corresponding Author:**	Yuseob Kim<br>Ewha Womans University<br>Seoul, KOREA, REPUBLIC OF
**Keywords:**	Fluctuation; storage effect; balancing selection; Population size; polymorphism; eco-evolutionary dynamics; positive feedback
**Abstract:**	Temporally variable environments in natural populations generate fluctuations in both the population size and the fitness effects of mutant alleles. The theory of storage effect, a species/allelic diversity-promoting mechanism discovered in ecology, predicts that rare mutants with fluctuating fitness can be positively selected and then maintained in balanced polymorphism if one part of the population, the ‘field’, is exposed to and the other, the ‘refuge’, is protected from fluctuating selection. Recent study found that oscillation in population size modifies the storage effect such that positive selection on a rare mutant occurs if its fitness fluctuation correlates with oscillation in the size of the field. By this new version of storage effect, mutants with even smaller geometric mean fitness than the wild-type can be selected to intermediate frequencies and remain in balanced polymorphism or reach fixation. To further understand these eco-evolutionary dynamics and test their generality in natural populations, this study built more realistic models that assume randomly, not cyclically, fluctuating selection and common demographic features, including heterogeneous ecological patches or an age-structured population. Mathematical analysis elucidated that whether balanced polymorphism or fixation occurs depends on the relative magnitudes of demographic versus fitness fluctuations and that both results require incomplete correlation in population size between the field and refuge. Therefore this novel evolutionary force is called ‘noncovarying storage effect (NSE)’. Multi-locus simulations revealed that oscillatory polymorphism can be maintained simultaneously or reach fixations at many loci. The latter occurs in a wider range of parameters and, if the fitness effects of mutations change the mean absolute fitness of the population, leads to positive feedback between demography and selection that causes a drastic amplification of population size fluctuation. These results suggest that the NSE is a potentially prevalent evolutionary force in nature for maintaining genetic variation or causing large demographic fluctuations.
**Order of Authors:**	Yuseob Kim
**Response to Reviewers:**	Response to reviewer comments are found in the "POneResonsToReview.pdf" file that was submitted along with the revised mansucript.
**Additional Information:**	
**Question**	**Response**
**Financial Disclosure**<br><br>Enter a financial disclosure statement that describes the sources of funding for the work included in this submission. Review the submission guidelines for detailed requirements. View published research articles from *PLOS ONE* for specific	Yes

examples.<br><br>This statement is required for submission and **will appear in the published article** if the submission is accepted. Please make sure it is accurate.<br><br>**Funded studies**<br>Enter a statement with the following details:<br>• Initials of the authors who received each award<br>• Grant numbers awarded to each author<br>• The full name of each funder<br>• URL of each funder website<br>• Did the sponsors or funders play any role in the study design, data collection and analysis, decision to publish, or preparation of the manuscript?<br><br>Did you receive funding for this work?	
Please add funding details.<br>as follow-up to "**Financial Disclosure**<br><br>Enter a financial disclosure statement that describes the sources of funding for the work included in this submission. Review the submission guidelines for detailed requirements. View published research articles from *PLOS ONE* for specific examples.<br><br>This statement is required for submission and **will appear in the published article** if the submission is accepted. Please make sure it is accurate.<br><br>**Funded studies**<br>Enter a statement with the following details:<br>• Initials of the authors who received each award<br>• Grant numbers awarded to each author<br>• The full name of each funder<br>• URL of each funder website<br>• Did the sponsors or funders play any role in the study design, data collection and analysis, decision to publish, or preparation of the manuscript?	This study was supported by the National Research Foundation (NRF) grant 2020R1A2C1009261 funded by the Korean government.

Did you receive funding for this work?"	
Please select the country of your main research funder (please select carefully as in some cases this is used in fee calculation).<br>as follow-up to "**Financial Disclosure**<br><br>Enter a financial disclosure statement that describes the sources of funding for the work included in this submission. Review the submission guidelines for detailed requirements. View published research articles from *PLOS ONE* for specific examples.<br><br>This statement is required for submission and **will appear in the published article** if the submission is accepted. Please make sure it is accurate.<br><br>**Funded studies**<br>Enter a statement with the following details:<br>• Initials of the authors who received each award<br>• Grant numbers awarded to each author<br>• The full name of each funder<br>• URL of each funder website<br>• Did the sponsors or funders play any role in the study design, data collection and analysis, decision to publish, or preparation of the manuscript?<br><br>Did you receive funding for this work?"	KOREA, REPUBLIC OF - KR
**Competing Interests**<br><br>Use the instructions below to enter a competing interest statement for this submission. On behalf of all authors, disclose any competing interests that could be perceived to bias this work—acknowledging all financial support and any other relevant financial or non-financial competing interests.	The author has declared that no competing interests exist

This statement is required for submission and **will appear in the published article** if the submission is accepted. Please make sure it is accurate and that any funding sources listed in your Funding Information later in the submission form are also declared in your Financial Disclosure statement.<br><br>View published research articles from *PLOS ONE* for specific examples.<br><br>**NO authors have competing interests**<br><br>Enter: *The authors have declared that no competing interests exist.*<br><br>**Authors with competing interests**<br><br>Enter competing interest details beginning with this statement:<br><br>*I have read the journal's policy and the authors of this manuscript have the following competing interests: [insert competing interests here]*<br><br>* typeset	
**Ethics Statement**<br><br>Enter an ethics statement for this submission. This statement is required if the study involved:<br><br>• Human participants<br>• Human specimens or tissue<br>• Vertebrate animals or cephalopods<br>• Vertebrate embryos or tissues<br>• Field research<br><br>Write "N/A" if the submission does not require an ethics statement.<br><br>General guidance is provided below. Consult the submission guidelines for detailed instructions. **Make sure that all information entered here is included in the Methods section of the manuscript.**	N/A

Powered by Editorial Manager® and ProduXion Manager® from Aries Systems Corporation

**Format for specific study types**<br><br>**Human Subject Research (involving human participants and/or tissue)**<br>• Give the name of the institutional review board or ethics committee that approved the study<br>• Include the approval number and/or a statement indicating approval of this research<br>• Indicate the form of consent obtained (written/oral) or the reason that consent was not obtained (e.g. the data were analyzed anonymously)<br><br>**Animal Research (involving vertebrate animals, embryos or tissues)**<br>• Provide the name of the Institutional Animal Care and Use Committee (IACUC) or other relevant ethics board that reviewed the study protocol, and indicate whether they approved this research or granted a formal waiver of ethical approval<br>• Include an approval number if one was obtained<br>• If the study involved *non-human primates*, add *additional details* about animal welfare and steps taken to ameliorate suffering<br>• If anesthesia, euthanasia, or any kind of animal sacrifice is part of the study, include briefly which substances and/or methods were applied<br><br>**Field Research**<br><br>Include the following details if this study involves the collection of plant, animal, or other materials from a natural setting:<br>• Field permit number<br>• Name of the institution or relevant body that granted permission	
**Data Availability**<br><br>Authors are required to make all data underlying the findings described fully available, without restriction, and from the time of publication. PLOS allows rare exceptions to address legal and ethical concerns. See the PLOS Data Policy and FAQ for detailed information.	Yes - all data are fully available without restriction

A Data Availability Statement describing where the data can be found is required at submission. Your answers to this question constitute the Data Availability Statement and **will be published in the article**, if accepted.<br><br>**Important:** Stating 'data available on request from the author' is not sufficient. If your data are only available upon request, select 'No' for the first question and explain your exceptional situation in the text box.<br><br>Do the authors confirm that all data underlying the findings described in their manuscript are fully available without restriction?	
**Describe where the data may be found in full sentences. If you are copying our sample text, replace any instances of XXX with the appropriate details.**<br><br>• If the data are **held or will be held in a public repository**, include URLs, accession numbers or DOIs. If this information will only be available after acceptance, indicate this by ticking the box below. For example: *All XXX files are available from the XXX database (accession number(s) XXX, XXX.).*<br>• If the data are all contained **within the manuscript and/or Supporting Information files**, enter the following: *All relevant data are within the manuscript and its Supporting Information files.*<br>• If neither of these applies but you are able to provide **details of access elsewhere**, with or without limitations, please do so. For example:<br><br>*Data cannot be shared publicly because of [XXX]. Data are available from the XXX Institutional Data Access / Ethics Committee (contact via XXX) for researchers who meet the criteria for access to confidential data.*<br><br>*The data underlying the results presented in the study are available*	Mathematica notebook files for conducting all simulations reported are available from https://github.com/YuseobKimLab/RandomFluctuationEcoEvo

from (include the name of the third party and contact information or URL).<br>• This text is appropriate if the data are owned by a third party and authors do not have permission to share the data.<br><br>* typeset	
Additional data availability information:	

Manuscript
Click here to access/download;Manuscript;DSCb0224_clean.docx

Noncovarying storage effect: balancing and positive directional selection on mutant alleles that amplify random fitness and demographic fluctuations

Yuseob Kim

Division of EcoScience, Ewha Womans University, Seoul, Korea 03760

yuseob@ewha.ac.kr

Running head: Eco-evolutionary fluctuation dynamics

Keywords: fluctuation, storage effect, population size, positive feedback, eco-evolutionary

Correspondence:

Yuseob Kim, Science Hall B308, Ewhayeodae-gil 52, Seodaemun-gu, Seoul, Korea 03760

Tel: +82 10 5138 5175

ABSTRACT

Temporally variable environments in natural populations generate fluctuations in both the population size and the fitness effects of mutant alleles. The theory of storage effect, a species/allelic diversity-promoting mechanism discovered in ecology, predicts that rare mutants with fluctuating fitness can be positively selected and then maintained in balanced polymorphism if one part of the population, the 'field', is exposed to and the other, the 'refuge', is protected from fluctuating selection. Recent study found that oscillation in population size modifies the storage effect such that positive selection on a rare mutant occurs if its fitness fluctuation correlates with oscillation in the size of the field. By this new version of storage effect, mutants with even smaller geometric mean fitness than the wild-type can be selected to intermediate frequencies and remain in balanced polymorphism or reach fixation. To further understand these eco-evolutionary dynamics and test their generality in natural populations, this study built more realistic models that assume randomly, not cyclically, fluctuating selection and common demographic features, including heterogeneous ecological patches or an age-structured population. Mathematical analysis elucidated that whether balanced polymorphism or fixation occurs depends on the relative magnitudes of demographic versus fitness fluctuations and that both results require incomplete correlation in population size between the field and refuge. Therefore this novel evolutionary force is called 'noncovarying storage effect (NSE)'. Multi-locus simulations revealed that oscillatory polymorphism can be maintained simultaneously or reach fixations at many loci. The latter occurs in a wider range of parameters and, if the fitness effects of mutations change the mean absolute fitness of the population, leads to positive feedback between demography and selection that causes a drastic amplification of population size fluctuation. These results suggest that the NSE is a potentially prevalent evolutionary force in nature for maintaining genetic variation or causing large demographic fluctuations.

ARTICLE SUMMARY

Natural populations experience rapid environmental changes that cause fluctuations in the fitness of genetic variants and population size. This study investigates how evolutionary changes due to fitness differences interact with concurrent demographic changes. The theory of the storage effect, which is a mechanism for promoting genetic diversity, was extended to add random population size fluctuation with heterogeneous environmental patches or in a population divided into different age classes. A novel evolutionary force that either increases genetic variation or drives evolution toward further amplification of population size fluctuation, an unconventional contributor to large demographic fluctuations in nature, was identified and analyzed.

INTRODUCTION

Natural populations experience temporal changes in abiotic and biotic environments. A randomly variable environment is expected to produce fluctuations in the fitness effects of non-neutral or phenotype-changing alleles segregating in the population. If the magnitude of fluctuation in the relative fitness is large, evolutionary changes may occur rapidly at the pace of short-term ecological/demographic fluctuations. Recent developments in evolutionary biology have revealed that such rapid evolutionary changes are widespread (Bell 2010; Thompson 2013; Messer *et al.* 2016; Lynch *et al*. 2024). Temporally variable environments should also generate fluctuations in various aspects of demography, most importantly the number of reproducing individuals (i.e., population size). One may then ask whether mutual interaction between demographic and selective fluctuations creates qualitatively novel eco-evolutionary dynamics that cannot arise if one of the demographic or population genetic variables is held constant.

One of major questions in the population genetics of fluctuating selection is whether different alleles that are favored in different time points can coexist in polymorphism. The prevalent conclusion has been that polymorphism is more difficult to be maintained under temporal fluctuation in selection than without fluctuation, as the allele under selection is pushed closer to frequency 0 or 1, after which it is more readily lost or fixed in the population by genetic drift (Hedrick et al. 1976; Hedrick 1986). However, recent studies suggested that temporal fluctuating selection can turn into balancing selection, an evolutionary force maintaining polymorphism at a level higher than expected under neutral evolution. It was shown that such balancing selection occurs if a population is partially protected from the cyclical selection, namely if fitness fluctuation occurs only in a subset of the population (Chesson & Warner 1981; Ellner & Hairston 1994; Reinhold 2000; Gulisija & Kim 2015; Gulisija *et al.* 2016; Bertram & Masel 2019; Yamamichi *et al.* 2023). Partial protection from fluctuating selection commonly occurs in many species. In spatially structured species the amplitude of fluctuation in selective pressure is likely to be heterogeneous over the geographic range. In plants and animals that have their life cycles divided into several stages and reproduce in overlapping generations, selective pressure is unlikely to act uniformly over individuals of all stages or ages. For example, if a trait under fluctuating selection is expressed in the insect larval stage, alleles affecting this trait currently carried by adults are not visible to the selection. Similarly, alleles affecting traits that appear after germination remain neutral in the seed bank of a plant species. Such a subset of the population protected from fluctuating selection may be called a "refuge." The presence of ~~a refuge~~ of various kinds was found to generate the advantage of rare alleles, an eco-evolutionary process commonly referred to as the storage effect

(Chesson 1983; Ellner & Hairston 1994; Reinhold 2000; Gulisija & Kim 2015; Gulisija *et al.* 2016; Bertram & Masel 2019; Yamamichi *et al.* 2023). A refuge acts to temporarily store the variants that otherwise will be removed from the population during unfavorable periods. More importantly, a refuge dampens fluctuation in the mean absolute fitness (per-capita rate of reproduction) of individuals carrying the rare allele under selection, which increases the geometric mean fitness of the rare allele above that of the common allele (Kim 2023). The storage effect was first theorized in community ecology, shown to arise not only with partial protection from fluctuation provided by a refuge but with general covariance between environment and competition, and is now one of the most important ecological theories for explaining the stable coexistence of species (Chesson & Warner 1981; Chesson 1994; Barabás *et al.* 2018). Imported into population genetics, it was recently proposed as one of a few mechanisms that may produce the genome-wide occurrence of oscillatory polymorphism, as observed in North American *Drosophila melanogaster* and other species, which appear to be maintained under fluctuating environments (Bergland et al. 2014; Park and Kim 2019; Johnson et al. 2023).

However, Kim (2023) showed that if the oscillation of population size is added to the model of storage effect a variant whose fitness also oscillates in the same directions can be either maintained in balanced polymorphism or positively selected at all frequencies until it reaches fixation. Such a variant may be called a fitness-amplifying allele because the relative fitness of an individual carrying this allele is high when the absolute fitness of all individuals is also high due to temporary population growth. Mathematical analysis showed that whether balancing or directional selection occurs on the fitness-amplifying allele depends on the relative strengths of fitness versus population size fluctuations. Previous studies on balancing selection by the storage effect in a constant-sized population required the coexisting variants to confer identical geometric mean fitness to the carriers. Such variants, exhibiting the geometric-mean-preserving fitness fluctuation (GMF) as defined in Kim (2023), are however selected either to loss or fixation in the presence of oscillation in the size of population outside the refuge. One may therefore regard the population size oscillation as a factor that destroys the potential of storage effect to generate balanced polymorphism. However, this population size oscillation led to balancing selection on a different type of mutants — alleles conferring smaller geometric mean but identical arithmetic mean fitness compared to the wild-type. Since such AMF (arithmetic-mean-preserving fitness fluctuation) mutations, which are deleterious in a simple population (without a refuge), are probably more likely to occur in the genome than the GMF mutations, this version of storage effect modified by demographic fluctuation might be equally important or even more important in

generating balanced polymorphism in nature. It was also shown that AMF variants are positively selected all the way to fixation if demographic fluctuation is relatively stronger than fitness fluctuation. Furthermore, Kim (2023) found that, under a weak regulation of population density under which the population size can over- or undershoot the carrying capacity, the positive selection leads to the amplification of oscillation in mean absolute fitness, thus population size, due to the fixation of the fitness-amplifying allele. In summary, the addition of population size fluctuation was found to generate novel eco-evolutionary dynamics not predicted by the conventional model of the storage effect.

The generality of novel eco-evolutionary dynamics discovered in Kim (2023) may however be questioned since the major result was obtained from the simulation of cyclically (seasonally) and symmetrically changing environments under which the population size and fitness oscillations are fully correlated. Although a mathematical analysis found general conditions for determining which evolutionary outcome (the loss, balanced polymorphism, or fixation of the fitness-amplifying allele) is expected, this result was based on the model of only two alternating seasons with fixed sizes. Therefore further investigation using more realistic models is needed to obtain better theoretical understanding and to find out whether the novel eco-evolutionary dynamics can arise in a substantially wider range of biological settings.

This study broadens the scope of investigation in the following directions. First, mathematical models assuming random, rather than deterministic and symmetrically seasonal, fluctuations in the environment were built and analyzed. A model with seasonal fluctuation is suitable for cases in which one cycle of environmental changes spans multiple generations. However, for many species, one generation is not shorter than a seasonal cycle. Random fluctuation of carrying capacities and the direction/strength of selection over generations, with a varying degree of correlation between these fluctuations, is probably relevant to a wider range of species including plants that exhibit mast seeding. Second, new models of population structures and inter-deme differences in selection intensity were investigated. Whereas Kim (2023) focused on a population with a seed bank (Ellner & Hairston 1994; Turelli *et al.* 2001; Gremer & Venable 2014) in which the mutant is completely protected from selection in the refuge, this study uses a model (the two-patch or TP model) that assumes two demes or ecological patches, both reproducing and undergoing changes in the carrying capacity, under general heterogeneity in selection intensity. Selection in the refuge may not be completely neutral but simply weaker than that in the field. This TP model was also analyzed extensively by simulation to test its robustness against perturbations. Another model (the larval-subadult-adult or LSA model) assuming a population

with an age structure, in which the life-cycle is divided into the larval (juvenile), subadult (young adult), and (old) adult stages, was also explored by simulation. Third, for both TP and LSA models, multi-locus stochastic simulations were performed. Simulations showed that mutant alleles causing wider fluctuation in offspring number can reach fixation at multiple loci in a wide range of conditions. These fixations were found to occur at faster rates with increasing numbers of loci, as fluctuating population size and fluctuating selection reinforced each other in positive feedback.

As will be clarified in the mathematical analysis, a key ingredient for this novel eco-evolutionary dynamics is the presence of a refuge whose size (or carrying capacity) remains relatively invariant or does not covary with the size of the rest of the population. One may therefore call this evolutionary mechanism the "noncovarying storage effect (NSE)". The result suggests that the NSE arising under the general condition of heterogeneous fluctuating selection in structured populations likely contributes to maintaining non-neutral genetic variation or to increasing demographic variability. Analytical and simulation results for the TP model will be first presented, followed by simulation results for the LSA model.

Two-patch (TP) model

Consider a haploid population that reproduces in discrete generations. The outline of the life cycle is illustrated in Fig. 1A. The population is divided into two subpopulations or patches that are subject to different levels and/or phases of fluctuating selection (thus two-patch or TP model). For convenience, these subpopulations will be called the field and the refuge following the terminology of Kim (2023). The latter refers to a subpopulation where selection on a mutant allele is weaker. It is assumed that the population is initially fixed for the ancestral A_1 allele at a locus. Then, a new mutant allele A_2, subject to fluctuating selection arises. The fitness of an individual carrying A_2 relative to A_1 is given by $1 + S_t$ in the field and $1 + S_t^*$ in the refuge, where S_t and S_t^* are drawn each generation from a joint distribution with $\mathrm{E}[S_t] = \mathrm{E}[S_t^*] = 0$. While the specific form of the distribution is needed for simulation (below), for deriving analytic solution in the next section only the mean, variance, and covariance of the distribution will be needed. It should be noted that this study considers only AMF mutations; while the arithmetic mean of the fitness of A_2 over time is 1, its geometric mean is less than 1, in both subpopulations. Natural selection is thus expected to eliminate such an allele from a simple panmictic (unstructured) population.

A fluctuating environment causes fluctuations in the carrying capacities of both the field and

refuge, which are given by $K_F^{(t)}$ and $K_R^{(t)}$ respectively for generation t. The carrying capacity is defined as the expected number of (reproductive) individuals at the end of each generation. It is assumed that the carrying capacities of both subpopulations depend on common environmental factors that fluctuate randomly over time but that subpopulations may differ in the strengths of the dependence. The sizes of subpopulations, $N_F^{(t)}$ and $N_R^{(t)}$, after the reproduction step in generation t, may or may not match the corresponding carrying capacities depending on the strength of population density regulation (see below). Before a new generation starts, each individual in the field migrates to the refuge or remains in the field with probability m_{FR} and 1 - m_{FR}. Similarly, each individual in the refuge either migrates or not with probability m_{RF} and 1 - m_{RF}. Next, the size of the field (refuge) changes from $\tilde{N}_F^{(t)}$ ($\tilde{N}_R^{(t)}$) at the beginning of generation t to $N_F^{(t)}$ ($N_R^{(t)}$) at the end, where $\mathrm{E}[\tilde{N}_F^{(t)}] = (1 - m_{FR})N_F^{(t-1)} + m_{RF}N_R^{(t-1)}$ and $\mathrm{E}[\tilde{N}_R^{(t)}] = m_{FR}N_F^{(t-1)} + (1 - m_{RF})N_R^{(t-1)}$.

Let $n_{1\mathrm{F}}$ and $n_{2\mathrm{F}}$ be the numbers of individuals in the field carrying A_1 and A_2 alleles at the beginning (before the reproduction step) of generation t (thus $n_{1\mathrm{F}} + n_{2\mathrm{F}} = \tilde{N}_F^{(t)}$). In the reproduction step, two different assumptions can be made regarding the effect of the mean fitness of individuals on the total population size. When the allele with higher or lower fitness increases in frequency, the total population size may not change due to an ecological constraint. In other words, the absolute fitness of the allele is frequency-dependent while its relative fitness remains constant. Conversely the mutant allele may increase or decrease the absolute reproductive output of the carriers regardless of its frequency such that the population size can increase over or decrease below the carrying capacity, which is now interpreted as the expected population size assuming that all individuals carry the ancestral (A_1) allele. For convenience, these two modes of natural selection will be simply referred to as soft and hard selection, respectively, following the terminology in Wallace (1975). First, under soft selection, the final size of the field is constrained to $K_F^{(t)}$. The number of progeny born to each parent is given by $WK_F^{(t)}/\bar{N}_F^{(t)}$, where the fitness W is 1 for A_1 and $1 + S_t$ for A_2, and $\bar{N}_F^{(t)} = n_{1\mathrm{F}} + (1 + S_t)n_{2\mathrm{F}}$. Second, under hard selection, fitness-changing mutations lead to changes in not only allele frequencies but also in the population size. To this effect, the number of progeny per parent is now $WK_F^{(t)}/\tilde{N}_F^{(t)}$. Namely, when $q = n_{2\mathrm{F}}/(n_{1\mathrm{F}} + n_{2\mathrm{F}})$ is the relative frequency of A_2 at the beginning of a generation, the size of the field is expected to reach $(1 + qS_t)K_F^{(t)}$, which becomes the effective carrying capacity of the field under hard selection. Similarly, reproduction in the refuge occurs either with soft or hard selection, where

subscript F in the above expressions is replaced by R and W is 1 for A_1 and $1 + S_t^*$ for A_2. The model so far described only the expected changes in the numbers of A_1- and A_2-carrying individuals. However, as reproduction in a finite-sized population was implicitly assumed, how genetic drift occurs need to be specified for stochastic simulation. To set the variance of offspring number similar to that in the haploid version of Wright-Fisher model, each parent in both subpopulations is modeled to produce a Poisson number of offspring with the mean specified above.

Finally, how the environment fluctuates over time and how demographic and selective parameters — $K_F^{(t)}$, $K_R^{(t)}$, S_t, and S_t^* — respond need to be specified. The environmental condition is assumed to fluctuate randomly in each generation, therefore with no temporal autocorrelation. More specifically, fluctuations occur such that $K_F^{(t)} = K_{F0}\mathrm{e}^{U_t}$ and $K_R^{(t)} = K_{R0}\mathrm{e}^{V_t}$ where U_t and V_t, together with S_t and S_t^*, are drawn each generation from a joint normal distribution satisfying $\mathrm{E}[U_t] = \mathrm{E}[V_t] = \mathrm{E}[S_t] = \mathrm{E}[S_t^*] = 0$. The oscillation of carrying capacities is therefore symmetrical in the log scale, which is generally predicted in demographic models and observed in nature (Tuljapurkar & Orzack 1980; Myers 1988). Various assumptions can be made regarding the variance-covariance relationship among S_t, S_t^*, U_t, and V_t. However, the correlation among them satisfies that $\mathrm{Cov}[S_t, U_t - V_t]$ is greater than $\mathrm{Cov}[S_t^*, U_t - V_t]$, which gives the mathematical definition of the field and refuge. The specific joint distribution for these variables will be given below for simulation.

Analytic results: the fate of the A_2 allele

This section aims to predict the fate of the rare A_2 allele — whether it will be lost, fixed, or increased to intermediate frequencies and maintained polymorphic in the population — as a function of the demographic and fitness parameters in the TP model. For convenience in the derivation, only the expected changes in the numbers of A_1- and A_2-carrying individuals will be tracked while ignoring genetic drift. In addition, a fixed scheme of migration, $m_{FR} = 1 - m_{RF} = r$, that sends a constant fraction r of the population to the refuge regardless of the current location is assumed. (Below the dynamics will be examined in simulation without this restriction in the migration rate.) First, soft selection, under which $N_F^{(t)} = K_F^{(t)}$ and $N_R^{(t)} = K_R^{(t)}$, is assumed. Let n_1 and n_2 be the total numbers of individuals (counted after reproduction/selection and before migration) carrying A_1 and A_2 in generation t – 1 and n'_1 and n'_2 be their expected numbers in

generation t. Then, with $n_1 \gg n_2$,

$$\frac{n_2'}{n_2} \approx (1-r)\frac{K_F^{(t)}}{\tilde{N}_F^{(t)}}(1+S_t) + r\frac{K_R^{(t)}}{\tilde{N}_R^{(t)}}(1+S_t^*) = \frac{(1+S_t)K_F^{(t)}+(1+S_t^*)K_R^{(t)}}{K_F^{(t-1)}+K_R^{(t-1)}} \tag{1}$$

(Appendix A). This equation takes into account that the change in the absolute number of A_2 is determined first by demography, as the size of field changes from $\tilde{N}_F^{(t)}$ to $K_F^{(t)}$ and that of refuge from $\tilde{N}_R^{(t)}$ to $K_R^{(t)}$, and also by fitness $1+S_t$ and $1+S_t^*$. Whether the copy number of rare allele A_2 is expected to increase or decrease is determined by the expectation of $\log\left[\frac{n_2'}{n_2}\right]$ over demographic and fitness fluctuations. It is shown in Appendix A that the condition for the A_2 allele being positively selected (i.e. successfully invading the population initially fixed for A_1), $\mathrm{E}\left[\log\left[\frac{n_2'}{n_2}\right]\right] > 0$, is simplified to

$$\frac{\mathrm{Cov}[S-S^*,U-V]}{\mathrm{Var}[S]+\rho^2\mathrm{Var}[S^*]+2\rho\mathrm{Cov}[S,S^*]} = \frac{\mathrm{Cov}[S-S^*,\log[K_F/K_R]]}{\mathrm{Var}[S]+\rho^2\mathrm{Var}[S^*]+2\rho\mathrm{Cov}[S,S^*]} > \frac{1}{2\rho} \tag{2}$$

where $\rho = K_{R0}/K_{F0}$ (note that $K_F^{(t)} = K_{F0}\mathrm{e}^{U_t}$ and $K_R^{(t)} = K_{R0}\mathrm{e}^{V_t}$) represents the relative size of the refuge and super-/subscripts for time t in variables were omitted.

If it is assumed that selection on A_2 in the refuge is weaker than that in the field by a ratio δ, namely $S_t^* = \delta S_t$, the above inequality is simplified to

$$\Phi \equiv \frac{\mathrm{Cov}[S,\log[K_F/K_R]]}{\mathrm{Var}[S]} > \frac{(1+\delta\rho)^2}{2(1-\delta)\rho} \tag{3}$$

Therefore, the rare A_2 allele is positively selected if the covariance between its fitness and the log-ratio of the field to refuge size is large relative to the amplitude of the fitness fluctuation. This requires that the demographic fluctuation in the population be sufficiently large, as already shown in Kim (2023), and that, to allow ~~the~~ substantial fluctuation of $K_F^{(t)}/K_R^{(t)}$, the carrying capacity of the refuge should not covary strongly with that of the field. Since this requirement for the refuge is essential and distinguishes the current from the previous models of storage effect, the evolutionary force driving this positive selection may be called the noncovarying storage effect (NSE; see Discussion for examples of noncovarying and covarying storages). The conditions (2) and (3) are easier to meet as δ becomes small well below 1, namely when the fitness fluctuation of A_2 becomes either smaller in the refuge compared to the field or negatively correlated to S_t.

In a similar manner, the condition for the A_1 allele to invade a population initially fixed for A_2 under soft selection becomes

$$\frac{\mathrm{Cov}[S-S^*,U-V]}{(1+2\rho)\mathrm{Var}[S]+(2\rho+\rho^2)\mathrm{Var}[S^*]-2\rho\mathrm{Cov}[S,S^*]} < \frac{1}{2\rho} \tag{4}$$

(Appendix A) or

$$\Phi < \frac{1+\delta^2}{1-\delta} + \frac{(1+\delta\rho)^2}{2(1-\delta)\rho} \tag{5}$$

if $S_t^* = \delta S_t$.

In a simpler case with δ = 0, from inequalities (3) and (5), it is predicted that the A_1 and A_2 alleles coexist in the population if $1/(2\rho) < \Phi < 1 + 1/(2\rho)$ under soft selection. With $\Phi < 1/(2\rho)$, the A_2 allele is eliminated from the population, as the fitness fluctuation that reduces its geometric mean in the field is more important than population size fluctuation. With $\Phi > 1 + 1/(2\rho)$, the A_2 allele is predicted to reach fixation in the population.

Using the same approach, the fate of the A_2 allele under hard selection can be analyzed. Note that the carrying capacities of the field and refuge are modified to $(1 + qS_t)K_F^{(t)}$ and $(1 + qS_t^*)K_R^{(t)}$ with hard selection where q is the frequency of A_2. The frequency change of A_2 when rare is therefore effectively identical to that in soft selection. Therefore, the condition for A_2 invading the population fixed for A_1 is again given by inequalities (2) and (3). However, the condition for the fixation of A_2 is drastically different from that under soft selection. It is shown in Appendix A that the condition for $\mathrm{E}\left[\log\left[\frac{n_1'}{n_1}\right]\right] > 0$ for rare A_1 is exactly the condition for $\mathrm{E}\left[\log\left[\frac{n_2'}{n_2}\right]\right] < 0$ for rare A_2. Therefore, with $S_t^* = \delta S_t$, the condition for the A_1 allele to invade the population fixed for A_2 is approximately $\Phi < (1 + \delta\rho)^2/(2(1-\delta)\rho)$. This means that the fixation of A_2 is ensured if $\Phi > (1 + \delta\rho)^2/(2(1-\delta)\rho)$; once positively selected after its appearance by mutation, the A_2 allele is expected to increase in frequency all the way to fixation. This leads to the amplification of fluctuation in the size of the field, from $K_F^{(t)}$ to $(1 + S_t)K_F^{(t)}$. This result is consistent with the finding in Kim (2023) that, under weak density regulation of the population size, an allele with a wider oscillation in absolute fitness reaches fixation in the presence of refuge.

One-locus simulation

To validate the above analysis and explore further eco-evolutionary dynamics in the TP model, the one-locus stochastic simulation was performed. The demographic and fitness fluctuations were modeled by $U_t = a_U Z_1 + b_U Z_2$, $V_t = a_V Z_1 + b_V Z_3$, $S_t = a_S Z_1 + b_S Z_4$, and $S_t^* = a_{S^*} Z_1 + b_{S^*} Z_5$ where a_X (b_X) is a constant for determining the magnitude of change in variable X due to a

common environmental factor (other factors) and the Z_is are standard normal variates that were drawn independently each generation. Therefore, Z_1 represents the state of the environment affecting both population sizes and fitness. To avoid negative fitness, the distributions of S_t and S_t^* were truncated at the lower bound -0.999.

Simulations were first performed for a scenario in which A_2 allele is completely protected from selection in the refuge ($S_t^* = 0$) and migration follows a simple scheme ($m_{FR} = m_{RF} = 0.5$). As suggested by the analysis above, the major parameter of eco-evolutionary dynamics in this case is $\Phi = \mathrm{Cov}[S, U - V]/\mathrm{Var}[S] = (a_S a_U - a_S a_V)/(a_S^2 + b_S^2)$. Simulation results were obtained for various values of Φ under soft (Fig. 2A, B) and hard (Fig. 2C, D) selection. Initially the numbers of A_1 and A_2 individuals changed deterministically once the demographic and fitness parameters (U, V, and S) for a given generation were sampled from their joint distribution, effectively assuming that the sizes of the field and refuge were finite and fluctuating but very large so that genetic drift can be ignored (Fig. 2A). Various values of a_S and b_S were chosen so that Φ ranged from 0.3 to 2, while ρ was set to 1. Starting from 0.5, the frequency of the A_2 allele at the 5,000th generation was recorded for 1000 replicates per parameter set. As predicted, with $\Phi \leq 0.5$, the frequency of A_2 approached 0, thus confirming negative selection, and with $\Phi \geq 1.5$ the frequency approached 1, thus confirming positive directional selection. The distribution of the allele frequency was approximately uniform between 0 and 1 when $\Phi = 1$. Next, genetic drift and bidirectional mutation between two alleles were added to the above simulations (Fig. 2B). This weakened the strengths of all modes of selection. With $\Phi = 1$, more simulation runs ended with allele frequencies close to 0 and 1. However, the proportion of runs ending with intermediate allele frequencies was still larger than that observed when the alleles were set as neutral (dashed curve in Fig. 2B). Therefore, an evolutionary force to maintain polymorphism ~~beyond~~ the level under ~~the~~ neutrality, ~~namely~~ balancing selection, was confirmed.

Similarly, the fate of the A_2 allele under hard selection was examined. Stochastic simulation confirmed the approximation above predicting that A_2 is positively selected towards fixation if $\Phi > \frac{1}{2\rho} = 0.5$, although genetic drift and mutation weakened the trend (Fig 2C,D). Positive selection on both rare and common A_2 under the same condition means that this allele may not remain polymorphic for long in the population. Indeed, across all values of Φ there were fewer simulation runs ending with intermediate frequencies of A_2 compared to the simulation of neutral alleles subject to genetic drift and bidirectional mutations only (dashed curve in Fig. 2D). Therefore, balancing selection that was observed with soft selection did not arise with hard

selection.

Next, the cases in which A_2 allele is not completely neutral in the refuge was examined. A simple model setting $S_t^* = \delta S_t$ was used. This time, the A_2 allele is initially absent in the population but introduced with recurrent mutation. For a given parameter set simulation was run over at least 10^5 generations and the mean $\bar{H}$ of expected heterozygosity ($2q(1-q)$, where q is the relative frequency of A_2) and the proportion of time when $0 \le q < 0.1$, P_{01}, and $0.9 < q \le 1$, P_{90}, during simulation were recorded. Simulation with $S_t = S_t^* = 0$ but under the same demographic structure yielded $\bar{H}$ = 0.063 (average over 6×10^5 generations). Then, as balancing selection is defined to be a force yielding genetic variation above the neutral level, a result was classified as balanced polymorphism ("balanced" in Fig. 3) if $\bar{H}$ > 0.075. If $\bar{H}$ < 0.05 and $P_{01} > P_{90}$, it suggests that negative selection against A_2 was the dominant evolutionary force. Such results were therefore classified as the loss of A_2 ("lost" in Fig. 3), since recurrent losses of A_2 should be the major events in the simulation (confirmed by visual inspection of allele frequency trajectories; not shown). Conversely, results yielding $\bar{H}$ < 0.05 and P_{90} > 0.7, which indicates that positive directional selection on A_2 was the dominant force, were classified as the fixation of A_2 ("fixed" in Fig. 3). All others were classified as not distinguishable from the neutral evolution. Simulations largely confirmed the analytic approximations for the conditions for the loss, fixation, or balanced polymorphism of A_2 in a population initially fixed for A_1 (e.g., $\frac{(1+\delta\rho)^2}{2(1-\delta)\rho} < \Phi < \frac{1+\delta^2}{1-\delta} + \frac{(1+\delta\rho)^2}{2(1-\delta)\rho}$ for the balanced polymorphism under soft selection and $\Phi > \frac{(1+\delta\rho)^2}{2(1-\delta)\rho}$ for the fixation under hard selection; Fig. 3). It is shown that increasing δ above 0 makes it harder for balancing and positive directional selection on A_2 to occur; for a large value of δ, the fluctuation (variance) of S_t needs to be much smaller than its covariance with the fluctuation of $\log[K_F^{(t)}/K_R^{(t)}]$ (eq. 3). Small Var[S_t] then means that the relative strength of genetic drift affecting the evolutionary dynamics of A_2 is large, explaining the results not distinguishable from the neutrality with large δ and Φ in Figure 3. Other results not distinguishable from neutral evolution were obtained when the predicted effects are near the boundary between balancing and directional selection. Another important result is that negative δ, thus selection in the refuge being in the opposite direction, widens the parameter range for balancing selection.

Finally, the robustness of balancing and positive directional selection above to deviations from the simple migration scheme, $m_{FR} = 1 - m_{RF} = r$, was examined. In actual populations, migration rates may vary over time and may not be symmetrical between the field and refuge, at least in a

short time scale. New simulations therefore sampled migration rates m_{FR} and m_{RF} from uniform distributions in ranges $[m_1, m_2]$ and $[m_3, m_4]$, respectively, each generation (Table S1). With ρ = 1 (K_{F0} = K_{R0}), limiting both m_{FR} and m_{RF} close to 0.5 yielded the highest level of balanced polymorphism under soft selection. However, allowing random deviation from 0.5 (e.g. $[m_1, m_2]$ = $[m_3, m_4]$ = [0.3, 0.7]) only slightly lowered the level of polymorphism. Smaller migration rates generally disrupted balancing selection. Interestingly, the effect of m_{FR} and m_{RF} was not symmetric; balancing selection remained effective with m_{RF} close to 0.5 and m_{FR} less than 0.2, however not with the opposite ratio of rates (compare cases with $[[m_1, m_2], [m_3, m_4]]$ = [[0.1, 0.2], [0.4, 0.5]] and [[0.4, 0.5], [0.1, 0.2]] in Table S1). Similar effects of m_{FR} and m_{RF} on the directional increase of A_2 allele frequency under hard selection were observed.

In summary, one-locus simulations for the TP model not only confirmed the analytic approximations for the conditions under which the NSE leads to either balanced polymorphism or the fixation of the fluctuation-amplifying allele but also showed that the effect is robust to perturbations in the model such as genetic drift and randomness/asymmetry in the migration rate.

Multi-locus simulation

To investigate how the eco-evolutionary dynamics generated by the NSE in the TP model play out when there are many such loci in the genome, new sets of simulations were performed in which a haploid individual is modeled to have L linked loci, each carrying either the A_1 or A_2 allele described above. To avoid exploring too many parameter dimensions while focusing on the effect of increasing the number of loci, $S_t^* = 0$ and a fixed migration scheme ($m_{FR} = m_{RF} = 0.5$) are assumed. The simulation tracks the numbers of individuals of different haplotypes (see Appendix B). Epistasis is not considered; the relative fitness of haplotype i is given by $W_i = \prod_{l=1}^{L}(1 + S_{t,l})$, where $S_{t,l}$ is 0 if the lth locus of the haplotype carries A_1 and $a_S Z_1 + b_S Z_4$ if A_2. Z_1 is drawn once for all loci while Z_4 is drawn separately for different loci. Individuals randomly pair and undergo recombination by crossing-over such that the probability of recombination between adjacent loci is c per generation. The simulation starts with a population fixed for A_1 at all loci. Then A_2 arises by mutations with probability μ/locus/generation.

Two major outcomes of the NSE – balancing selection and positive directional selection – were addressed. First, considering that (1) the negative frequency-dependent selection on the A_2 allele

was caused primarily by its fitness correlated to the fluctuation in the carrying capacity (population size) of the field and that (2) this demographic fluctuation should be "felt" at all loci in the genome, one may expect that such balancing selection can occur simultaneously at many loci harboring such variants. To verify if this is true, simulations were run under soft selection and demographic/selective parameters that promoted polymorphism in the single-locus model (Φ = 1 for ρ = 1) with L = 1, 4, or 10. Exemplary trajectories of allele frequencies are shown in Fig. S1. Each run lasted at least 10^5 generations over which $\bar{H}$, the expected heterozygosity $(2p(1-p))$ averaged over time and loci, and the proportion of time the A_2 alleles spent in frequency between 0.1 and 0.5, P_{15}, and between 0.5 and 0.9, P_{59}, were recorded (Table 1). In all cases with fluctuating fitness ($a_S > 0$), $\bar{H}$ was above the level observed in equivalent simulations with neutral alleles (S_t = 0), thus confirming multi-locus balancing selection. For cases of stronger selection (larger Var[S_t]), the level of polymorphism per locus declined as L increased from 1 to 10. The combined effects of the number of loci and the rate of recombination were not easy to interpret as they depended on the parameters of selection (for sets of fitness/demographic parameters that yield the same Φ = 1, $\bar{H}$ responded differently with varying L and c). One possible explanation for generally lower variation with 10 loci instead of 1 or 4 is that the simultaneous segregation of alleles at many loci increased the variance of the offspring numbers, thus decreasing the effective population size and weakening the negative frequency-dependent selection that is needed for protected polymorphism. Further exploration of multi-locus polymorphism at a larger scale (in both number of loci and population size) may not be feasible with the current simulation approach based on haplotype frequencies.

Second, simulation was performed with parameter values under which directional selection for the A_2 allele is expected in the single-locus model ($\Phi > 1 + 1/(2\rho)$ for soft selection and $\Phi > 1/(2\rho)$ for hard selection). As expected, substitutions occurred sequentially until A_2 reached fixation at all L loci (Fig. 4A and Fig. S2 for example). Times (in generations) when the frequency of A_2 first exceeded 0.9 at each locus were recorded and sorted into T_1 to T_L. There is a large difference in substitution dynamics between soft and hard selection. With soft selection, the mean waiting time, $\bar{T} = (T_1 + \cdots + T_L)/L$, increased moderately as L increased from 1 to 8 (Fig. 5A). This may be explained by a reduction in the efficacy of positive selection on A_2 at a given locus when selection at other loci results in genome-wide reduction in the effective population size.

With hard selection, however, $\bar{T}$ decreased as L increased. Furthermore, the coefficient of variation, the standard deviation of T_i's divided by $\bar{T}$, was significantly smaller with hard selection than with soft selection (Fig. 5B). As expected for hard selection, the amplitude of the population

size fluctuation progressively increased as the number of loci fixed for A_2 increased (Fig. 4A). These results clearly demonstrate that positive feedback between demographic and selective fluctuations under hard selection led to a great amplification of population size fluctuation; an initial fluctuation in $K_F^{(t)}$ positively selected the mutant allele A_2 at one locus whose fitness fluctuates in positive correlation with $K_F^{(t)}$, leading to the fixation of A_2 and therefore a larger fluctuation of the field (given by $(1+S_t)K_F^{(t)}$), which makes it easier for the A_2 allele at another locus to be positively selected. This process could continue to increase the fluctuation of the field until its carrying capacity is given by $(1+S_t)^L K_F^{(t)}$, where L is the number of loci available in the genome for mutation into A_2. There was also a moderate effect of the recombination rate in accelerating the multi-locus fixation of A_2 with hard selection (Table S2). Fixation times generally increased with decreasing recombination rates for both soft and hard selection, possibly because tighter linkage increased the occurrence of clonal interference.

Larval-subadult-adult (LSA) model

Although the refuge in the TP model is expected to play the role of various demographic/genetic elements that were previously shown to produce the storage effect, such as seed banks, diapausing stages, or a particular life-stage in a population with overlapping generations, the noncovarying storage effect found in the TP model may not be replicated in other population structures as the nature of refuge (i.e. whether it undergoes its own reproduction and whether the lineage of an allele can persist in a refuge for many generations) and the pattern of 'migration' in and out of the refuge can be qualitatively different. I therefore investigate an explicit discrete-time model of an age-structured population with overlapping generations. Three stages – 'larval', 'subadult', and 'adult' – in the life cycle of a haploid species are assumed. Subadult means the early stage of reproducing adults. Time is counted in intervals of the length required for one life stage to advance to the next one. At the end of each interval, the final size of larval population is regulated to its carrying capacity ($K_L^{(t)}$ at time t). Probabilities for an individual to advance from larval to subadult and from subadult to adult stages are given by e_L and e_S, respectively. Therefore, the sizes of the subadult and adult populations are expected to be $e_L K_L^{(t-1)}$ and $e_L e_S K_L^{(t-2)}$ at time t. $K_L^{(t)}$ is assumed to vary randomly over time with no autocorrelation. Specifically it is modeled to be $K_L^{(t)} = K_{L0}\exp(a_L Z_1 + b_L Z_2)$, where Z_1 and Z_2 are standard normal variables drawn each time.

The population is made of haploids carrying either the A_1 or A_2 allele at each of L linked loci. The (marginal) fitness of A_2 relative to A_1 at locus j is given by $1 + S_{j,t}$ (j = 1, …, L), where $S_{j,t} = a_S Z_1 + b_S Z_{3,j}$. $Z_{3,j}$ is drawn independently for different loci at each interval t. Then, the fitness of haplotype i, $W_i^{(t)}$, is given by the multiplication of allelic fitness across all loci. It is assumed that this haplotype fitness determine the viability in the larval stage only; the carriers of two alleles are not different either in the probability of advancing to the next stage or in fertility in the subadult and adult stages. If there are only larval and subadult stages, they constitute a population that reproduces in effectively discrete generations. The addition of the (old) adult stage provides a storage for alleles regardless of their reproductive success in the previous (subadult) stage. Therefore, the adult stage plays the role of the refuge and is an example of a noncovarying storage because its size is independent of the concurrent subadult stage.

Multi-locus simulations, allowing not only genetic drift in the reproduction step but also stochastic advances from one to the next life stage (see Appendix B for method), were performed with limited sets of parameters, each starting with a population fixed for haplotype 0, which carries A_1 at all loci. The evolutionary dynamics of the A_2 alleles in each simulation run over 50,000 time units was summarized by the mean heterozygosity and the proportion of time A_2 remained close to relative frequency 0 or 1 (Table S3). Major results produced in the TP model – multi-locus balancing selection under soft selection or positive directional selection on A_2 under hard selection – were readily obtained with this model. In agreement with Kim (2023) and the TP model, the relative strength of demographic vs. fitness fluctuation determined the outcome; when we define $\Phi' = \mathrm{Cov}\left[\log[K_L^{(t)}/K_{L0}], S_{j,t}\right]/\mathrm{Var}\left[S_{j,t}\right] = a_L a_S/(a_S^2 + b_S^2)$, which is analogous to Φ in the TP model, positive selection on rare A_2 occurred when Φ' was above 0.7. With soft selection and above this threshold of Φ', the frequency of A_2 underwent sustained oscillations that are indicative of balancing selection (Fig. S3). Then, as Φ' continued to increase, balancing selection transitioned into positive directional selection. With hard selection, positive directional selection occurred once Φ' increased above the threshold. Exemplary replicates show that, as the A_2 alleles reach fixation across loci, fluctuations in the sizes of all three life stages become amplified (Fig. 4B, Fig. S3). Negative (positive) correlation between mean fixation times and the number of loci under hard (soft) selection and the clustering of fixation times with hard selection were also observed in other sets of simulations (Fig. S4), which basically replicated the results from the TP model (Fig. 5). This confirmed that positive feedback between demographic and fitness fluctuations under hard selection occurs in both models. In conclusion, all major aspects of the

NSE found in the TP model were shown to arise in the LSA model as well.

DISCUSSION

A key assumption in this study is that a tradeoff (antagonistic environmental pleiotropy; Zhang 2023) commonly occurs for the fitness effects of a mutant allele between different time points in the environmental fluctuation. Namely, a mutant phenotype that increases the reproductive output relative to others when the environment is generally favorable is likely to suffer a decline in reproductive output relative to others during an unfavorable period. Such a mutant allele increases the variance of the offspring number over time while the mean may remain unchanged. For example, as postulated by Slatkin (1974), a mutation that makes a female bird lay 10 eggs in one batch instead of spreading them over time in a randomly and rapidly fluctuating environment will increase the variance of her fitness while the mean remains the same. Setting the fitness of a mutant to $1 + S_t$ with $E[S_t] = 0$, thus an AMF mutant, models such a tradeoff. Classical theory predicts that such a mutant is eliminated from a population as its geometric mean fitness is less than 1.

Kim (2023) and this study however found that an AMF mutant at a low frequency can be positively selected (with both soft and hard selection) if a correlation between demographic and fitness fluctuations exists in the presence of a refuge whose carrying capacity oscillates in a narrower range and/or out of phase relative to the field. A heuristic argument for this NSE on the fitness-amplifying mutant allele may be given. In the TP model, the mean number of offspring per parent, *i.e.* the absolute fitness, of the rare mutant in the field is given by $W_{2F} = \frac{K_F^{(t)}}{\tilde{N}_F^{(t)}}(1 + S_t)$ (eq. 1), where $K_F^{(t)}/\tilde{N}_F^{(t)}$ is the absolute fitness given under demographic fluctuation alone. With a positive correlation between $K_F^{(t)}$ and S_t, the fluctuation of W_{2F} becomes greater than that of $1 + S_t$ and, more importantly, its arithmetic mean can now exceed 1. Still, the geometric mean of W_{2F} is not greater than 1 due to its fluctuation over time. However, the presence of a refuge can reduce the variance of the mutant's fitness averaged across the whole population such that the geometric mean of this average fitness becomes greater than 1. Here, to generate this dampening effect, the refuge should not undergo the same fluctuation in size with the field; the absolute fitness in the refuge $W_{2R} = \frac{K_R^{(t)}}{\tilde{N}_R^{(t)}}(1 + S_t^*)$ in eq. (1) should fluctuate differently than W_{2F}. With parameter values used in of the TP model, $K_R^{(t)}$ was given to covary incompletely with $K_F^{(t)}$. In

the LSA model, the fluctuation of the refuge (the adult stage) is uncorrelated to the fluctuation of the field (the subadult stage). In Kim (2023), the seed bank fluctuates in size similar to the subadult and adult stages in the LSA model. In summary, such refuges, or noncovarying storages, turn the heightened arithmetic mean of the mutant allele raised by the demographic fluctuation of the field into an elevation of the geometric mean in the total population.

Not all refuges in the previously proposed mechanisms of the storage effect are noncovarying. For example, it was suggested that if a gene is expressed in one sex in a dioecious species the other sex could be a refuge (Reinhold 2000; Kim 2023). However, as equal numbers of male and female gametes are used in reproduction, the subpopulation sizes of reproductive males and females are effectively equal while the sex ratio of the adults may fluctuate. Therefore, as the size of the field relative to the refuge does not fluctuate at all, the NSE should not arise for the genes of the sex-limited expressions.

One may also assume GMF mutations; the fluctuating fitness of a mutant can be given by $\exp[S_t]$, for example, making the mutant quasi-neutral as its geometric mean fitness is identical to that of the wild-type allele even without demographic fluctuation. Analyzing the model of cyclic environmental oscillation, Kim (2023) showed that the parameter range of positive selection for a rare GMF allele is wider than that for an AMF allele. Similarly, GMF alleles should be subject to the NSE under broader conditions. However, a GMF allele may be considered simply a beneficial allele as the arithmetic mean of offspring number is greater than the wild-type allele. Such a mutation is probably rare and therefore was not considered in this study. One should however note that GMF alleles might be still important for genetic variation in nature since they can be maintained in balanced polymorphism in the conventional models of the storage effect, for example without demographic fluctuations and/or in the case of sex-limited gene expressions.

Analytic approximation for the one-locus TP model predicts that, under soft selection or strict density regulation that keeps the population size to the carrying capacity of the field at a given time regardless of its genetic composition, balancing selection arises if the demographic fluctuation (a major contributor to $\mathrm{Cov}[S, \log[K_F/K_R]]$) is not so weak or strong compared to fitness fluctuation ($\mathrm{Var}[S]$ in case of $S^* = 0$). Multi-locus simulation showed that balanced polymorphism can be simultaneously maintained at many loci, as expected since the demographic fluctuation (together with the presence of refuge) that turns the geometric mean fitness of an AMF allele from < 1 to > 1 should apply equally to all loci in the genome. Previously, Park and Kim (2019) showed that, in the presence of a refuge but without fluctuation in population size,

polymorphism can arise at multiple loci that contribute to the expression of a phenotype under fluctuating selection. However, the number of loci where balanced polymorphism emerged increased very slowly as the total number of loci in the model increased. In contrast, in the current simulation, balanced polymorphism was observed at all loci available for mutations to arise. However, the model containing only up to 10 loci was examined, due to the limitation of the simulation method used, and the 10-loci polymorphism at each locus was generally lower than the level observed in the one-locus simulation. This is probably because the segregation of alleles at multiple loci elevates variance in the offspring number well above that of the Poisson distribution, thus increasing genetic drift that weakens the positive selection on rare alleles. The possibility that this mechanism can maintain the genome-wide polymorphism over 1,000 loci, currently observed in several natural populations (Bergland, et al. 2014; Johnson, et al. 2023), should be addressed by a simulation on a large scale in terms of both the number of loci and the census population size.

If the mutant (A_2) alleles that were positively selected from low to high frequencies are not maintained as a stable, oscillatory polymorphism as described above, they are fixed in the population. Such events, the fixations of mutations that amplify fitness fluctuations are another outcome of the NSE with eco-evolutionary significance. In the TP model, the condition for the fixation is less restrictive compared to that for balanced polymorphism, because it requires relatively weaker selection (smaller variance in relative fitness) for a given demographic fluctuation to satisfy, assuming $S^* = 0$, $\Phi > 1 + 1/(2\rho)$ with soft selection and $\Phi > 1/(2\rho)$ with hard selection. Similar ranges of conditions (for Φ') seem to apply for the LSA model (Table S3). Because fixation rather than balanced polymorphism occurs in a wider parameter space, one may argue that the overall effect of NSE (i.e. the modification of the storage effect due to population size fluctuation) is to make balancing selection less likely to occur. However, given the possible patterns of mutant alleles' fitness fluctuations in nature beyond those considered so far, the NSE may be a force of increasing, rather than decreasing, the number of loci in which oscillatory polymorphism occurs. First, the NSE causes all GMF mutants to be either lost or fixed in the population, thus no balanced polymorphism (Kim 2023), but allows AMF mutants to be maintained as balanced polymorphism. As one may expect more AMF mutations than GMF mutations to occur in the genome, since more beneficial mutations (in net effect) are considered less likely in general, the number of loci under balancing selection may increase due to the NSE. This also suggests that for a given demographic fluctuation, mutations of certain fluctuation in fitness are "picked up" to be maintained as polymorphism. In the heuristic argument above, the

absolute fitness of the A_2 allele in the field is given by $W_{2F} = D_F w_2$, where D_F is its absolute fitness given by change in the population size of the field alone and w_2 is the relative fitness of A_2 in the field (e.g., in the TP model, $D_F = \frac{K_F^{(t)}}{\tilde{N}_F^{(t)}}$ and $w_2 = 1 + S_t$). It is a limited range of fluctuation in W_{2F} that produce polymorphism, as suggested in the above model with AMF mutations where a larger fluctuation in population size (thus in D_F) allows polymorphism only when the mutant alleles have a larger fluctuation in fitness relative to the wild-type (so that Var[S] matches Cov[S, log[K_F/K_R]]), which makes the arithmetic mean of W_{2F} decline. It might be possible that for a strong demographic fluctuation under which an AMF mutant is driven to fixation, a mutant with a new pattern of fitness fluctuation not considered in this study so far, possibly with even lower arithmetic mean than AMF mutants (thus < 1), might be picked up by the NSE into balanced polymorphism.

Demographic fluctuation that generates the NSE should apply equally to all loci in the genome. Therefore, fixations occur at all available loci on which fitness-amplifying mutations (*i.e.* the A_2 allele) arise, as observed in the multi-locus simulation. With soft selection, the pattern of demographic fluctuation that generates the NSE does not change over time regardless of any evolutionary change at any locus. However, with hard selection, fluctuation in the relative size of the field generating the NSE at a given locus intensifies as more fluctuation-amplifying mutations reach fixation at other loci. Since the carrying capacity of the field in the TP model is effectively $(1 + S_t)^l K_F^{(t)}$, where l is the number of loci where A_2 reached fixation, and wider fluctuation generates stronger positive selection on the rare A_2 (larger $\mathrm{E}[\Delta_2]$ in Appendix A), fixation at one locus facilitates the subsequent fixations at other loci. This positive feedback between demographic fluctuation and positive selection drastically increases the amplitude of fluctuation in the total population size (Fig. 4). How much the amplitude will increase should depend on the number of loci at which mutations subject to hard selection arise. There should be multiple independent ways in which changes in mutants' relative fitness can be translated into changes in the mean absolute fitness of the entire population. For various life history traits, one may imagine phenotypes (smaller eggs, smaller adult body, earlier onset of first reproduction, etc.) that increase the absolute number of surviving offspring during a favorable, resource-rich period but decrease the number in a bad period while not affecting the amount of resources that the carriers of the alternative, ancestral alleles acquire. Then, many loci may be available for hard selection, the combined effect of which may lead to a very large fluctuation in population size.

The size of a population and its fluctuation should be governed not only by extrinsic factors (the

environmental variables such as temperature and parasite prevalence) but also by intrinsic factors (species-specific responses to those extrinsic factors). The intrinsic factors such as various life-history traits are the product of the species' evolutionary history. For example, phenotypic plasticity responding to environmental cues can reduce the amplitude of the population size fluctuation, thus acting as a mechanism of population regulation (Caswell 1983; Reed *et al.* 2010), which according to the classical theory is adaptive as it enhances long-term reproductive success by increasing the geometric mean fitness (Gillespie 1977; Tuljapurkar 1982; Pfister 1998). If life history traits evolved in the direction toward suppressing random or cyclic fluctuations in the population size (Pfister 1998), fluctuations observed in nature should be interpreted as the effect of variable environments that the evolved mechanisms of population regulation were unable to contain. However, this study suggests that evolution can proceed in the opposite direction. A large random fluctuation in population size can be the product of adaptive evolution driven by positive feedback between demographic and selective fluctuations. A recent study also suggested that a species may evolve to undergo a larger fluctuation in its population size. Liu *et al.* (2019) found that a reproductive strategy increasing the temporal variance of offspring number can be advantageous in populations reproducing in overlapping generations under various patterns of environmental fluctuations. When the degrees of population size fluctuation are different across species, the eco-evolutionary dynamics proposed in this study might be particularly important for those exhibiting large fluctuations, for example mast seeding or insect outbreaks (Dwyer *et al.* 2004; White 2008; Bogdziewicz *et al.* 2020). Interestingly, annual desert plants experiencing wider fitness fluctuations after germination have greater proportions of their population remaining in the seed bank (Gremer & Venable 2014). This observation is in agreement with the analytic results in this study because a larger seed bank corresponds to a larger value of ρ, which makes it easier for the fixation of the fluctuation-amplifying alleles (i.e., lowering the threshold of Φ for positive selection) by the NSE.

DATA AVAILABILITY

Mathematica notebook files for conducting all simulations reported are available from https://github.com/YuseobKimLab/RandomFluctuationEcoEvo

ACKNOWLEDGMENT

I thank Kristan Schneider, Claus Vogl, Jason Wolf, Sally Otto, and anonymous reviewers who read the previous and earlier versions of the manuscript and provided comments that greatly improved the scope and depth of this study.

CONFLICT OF INTEREST

The author declares no conflict of interest.

REFERENCES

Barabás G, D'Andrea R, Stump SM. (2018). Chesson's coexistence theory. *Ecological monographs* 88:277-303.

Bell, G. (2010). Fluctuating selection: the perpetual renewal of adaptation in variable environments. *Philos Trans R Soc Lond B Biol Sci*, 365, 87-97.

Bergland AO, Behrman EL, O'Brien KR, Schmidt PS, Petrov DA. (2014). Genomic evidence of rapid and stable adaptive oscillations over seasonal time scales in Drosophila. *PLoS Genet,* 10, e1004775.

Bertram, J. & Masel, J. (2019). Different mechanisms drive the maintenance of polymorphism at loci subject to strong versus weak fluctuating selection. *Evolution*, 73, 883-896.

Bogdziewicz, M., Ascoli, D., Hacket-Pain, A., Koenig, W.D., Pearse, I., Pesendorfer, M. *et al.* (2020). From theory to experiments for testing the proximate mechanisms of mast seeding: an agenda for an experimental ecology. *Ecology Letters*, 23, 210-220.

Caswell, H. (1983). Phenotypic plasticity in life-history traits: demographic effects and evolutionary consequences. *American Zoologist*, 23, 35-46.

Chesson, P.L. (1983). Coexistence of competitors in a stochastic environment: the storage effect. *Lecture Notes in Biomathematics* 54:188-198.

Chesson, P.L. (1994). Multispecies competition in variable environments. *Theoretical Population Biology*, 45, 227-276.

Chesson, P.L. & Warner, R.R. (1981). Environmental variability promotes coexistence in lottery competitive systems. *The American Naturalist*, 117, 923-943.

Dwyer, G., Dushoff, J. & Yee, S.H. (2004). The combined effects of pathogens and predators on insect outbreaks. *Nature*, 430, 341-345.

Ellner, S. & Hairston, N.G. (1994). Role of overlapping generations in maintaining genetic variation

in a fluctuating environment. *The American Naturalist*, 143, 403-417.
Gillespie, J.H. (1977). Natural selection for variances in offspring numbers: a new evolutionary principle. *The American Naturalist*, 111, 1010-1014.
Gremer, J.R. & Venable, D.L. (2014). Bet hedging in desert winter annual plants: optimal germination strategies in a variable environment. *Ecol Lett*, 17, 380-387.
Gulisija, D. & Kim, Y. (2015). Emergence of long-term balanced polymorphism under cyclic selection of spatially variable magnitude. *Evolution*, 69, 979-992.
Gulisija, D., Kim, Y. & Plotkin, J.B. (2016). Phenotypic plasticity promotes balanced polymorphism in periodic environments by a genomic storage effect. *Genetics*, 202, 1437-1448.
Hedrick, P.W. (1986). Genetic polymorphism in heterogeneous environments: a decade later. *Annual Review of Ecology and Systematics* 17:535-566.
Hedrick, P.W., Ginevan, M.E. & Ewing, E.P. (1976). Genetic polymorphism in heterogeneous environments. *Annual Review of Ecology and Systematics* 7:1-33.
Johnson O.L, Tobler R, Schmidt J.M., Huber C.D. (2023). Fluctuating selection and the determinants of genetic variation. *Trends in Genetics,* 39, 491-504
Kim, Y. (2023). Partial protection from fluctuating selection leads to evolution towards wider population size fluctuation and a novel mechanism of balancing selection. *Proceedings of the Royal Society B*, 290, 20230822.
Liu, M., Rubenstein, D.R., Liu, W.-C. & Shen, S.-F. (2019). A continuum of biological adaptations to environmental fluctuation. *Proceedings of the Royal Society B*, 286, 20191623.
Lynch, M., Wei, W., Ye, Z. & Pfrender, M. (2024). The genome-wide signature of short-term temporal selection. *Proceedings of the National Academy of Science USA*, 121:e2307107121.
Messer, P.W., Ellner, S.P. & Hairston, N.G. (2016). Can population genetics adapt to rapid evolution? *Trends in Genetics*, 32, 408-418.
Myers, J.H. (1988). Can a general hypothesis explain population cycles of forest Lepidoptera? In: *Advances in Ecological Research*. Elsevier, pp. 179-242.
Park Y, Kim Y. 2019. Partial protection from cyclical selection generates a high level of polymorphism at multiple non-neutral sites. *Evolution*, 73, 1564-1577.
Pfister, C.A. (1998). Patterns of variance in stage-structured populations: evolutionary predictions and ecological implications. *Proceedings of the National Academy of Sciences*, 95, 213-218.
Reed, T.E., Waples, R.S., Schindler, D.E., Hard, J.J. & Kinnison, M.T. (2010). Phenotypic plasticity and population viability: the importance of environmental predictability. *Proceedings of the Royal Society B: Biological Sciences*, 277, 3391-3400.

Reinhold, K. (2000). Maintenance of a genetic polymorphism by fluctuating selection on sex-limited traits. *Journal of Evolutionary Biology*, 13, 1009-1014.

Slatkin, M. (1974). Hedging one's evolutionary bets. *Nature*, 250, 704-705.

Thompson, J.N. (2013). *Relentless evolution*. University of Chicago Press.

Tuljapurkar, S. & Orzack, S.H. (1980). Population dynamics in variable environments I. Long-run growth rates and extinction. *Theoretical Population Biology*, 18, 314-342.

Tuljapurkar, S.D. (1982). Population dynamics in variable environments. III. Evolutionary dynamics of r-selection. *Theoretical population biology*, 21, 141-165.

Turelli, M., Schemske, D.W. & Bierzychudek, P. (2001). Stable two-allele polymorphisms maintained by fluctuating fitnesses and seed banks: protecting the blues in Linanthus parryae. *Evolution*, 55, 1283-1298.

Wallace, B. (1975). Hard and soft selection revisited. *Evolution*, 465-473.

White, T. (2008). The role of food, weather and climate in limiting the abundance of animals. *Biological Reviews*, 83, 227-248.

Yamamichi, M., Letten, A.D. & Schreiber, S.J. (2023). Eco-evolutionary maintenance of diversity in fluctuating environments. *Ecology Letters*, 26, S152-S167.

Zhang, J. (2023). Patterns and evolutionary consequences of pleiotropy. *Ann Rev Ecol Evol Syst* 54:1-19.

Appendix A. Derivation of conditions for balancing and directional selection on A_2 in the TP model

Here only deterministic changes in allele frequencies are tracked as a very large population is assumed. At the end of reproduction but before migration into subpopulations at generation t - 1, there are n_1 and n_2 copies (individuals) of A_1 and A_2 in the entire population. After migration, the numbers of A_1 and A_2 copies are $n_{1F} = (1-r)n_1$ and $n_{2F} = (1-r)n_2$ in the field and $n_{1R} = rn_1$ and $n_{2R} = rn_2$ in the refuge. Therefore, the relative frequencies of alleles are identical between the field and refuge before reproduction at the beginning of generation t.

Soft selection is first considered. Then, each A_2 allele produces $(1+S_t)K_F^{(t)}/\overline{N}_F^{(t)}$ copies of descendants, where $\overline{N}_F^{(t)} = n_{1F} + (1+S_t)n_{2F} = (1-r)(n_1 + (1+S_t)n_2)$. Since $n_1 \gg n_2$ after A_2 appears by mutation, $n_1 + (1+S_t)n_2 \approx n_1$ and $\overline{N}_F^{(t)} \approx \widetilde{N}_F^{(t)} = (1-r)(n_1+n_2) = (1-r)\left(K_F^{(t-1)} + K_R^{(t-1)}\right)$. Similar approximations apply to the refuge. Let n'_2 be the expected number

of A_2 in generation t. This leads to eq. (1). Namely,

$$\frac{n_2'}{n_2} = (1-r)\frac{(1+S_t)K_F^{(t)}}{\bar{N}_F^{(t)}} + r\frac{(1+S_t^*)K_R^{(t)}}{\bar{N}_R^{(t)}} \approx (1-r)\frac{(1+S_t)K_F^{(t)}}{\tilde{N}_F^{(t)}} + r\frac{(1+S_t^*)K_R^{(t)}}{\tilde{N}_R^{(t)}} = \frac{(1+S_t)K_F^{(t)}+(1+S_t^*)K_R^{(t)}}{K_F^{(t-1)}+K_R^{(t-1)}}$$

Then, with $K_F^{(t-1)} = K_{F0}\mathrm{e}^{U}$, $K_R^{(t-1)} = K_{R0}\mathrm{e}^{V}$, $K_F^{(t)} = K_{F0}\mathrm{e}^{U'}$, and $K_R^{(t)} = K_{R0}\mathrm{e}^{V'}$,

$$\Delta_2 \equiv \log\left[\frac{n_2'}{n_2}\right] = \log\left[\mathrm{e}^{U'}(1+S) + \rho\mathrm{e}^{V'}(1+S^*)\right] - \log[\mathrm{e}^{U} + \rho\mathrm{e}^{V}],$$

where $S = S_t$, $S^* = S_t^*$, and $\rho = K_{R0}/K_{F0}$. If $\mathrm{E}[\Delta_2] > 0$, where the expectation is over demographic and fitness fluctuations, the rare allele A_2 is expected to increase its copy number over time. As the expectations of U, V, U', V', S, and S^* are all zero and non-zero correlations exist only among U', V', S, and S^* and between U and V, from the Taylor expansion of Δ_2 up to the second order, it can be shown that

$$\begin{aligned}\mathrm{E}[\Delta_2] \approx\ & \frac{1}{2}\alpha_{U'U'}\mathrm{Var}[U'] + \frac{1}{2}\alpha_{V'V'}\mathrm{Var}[V'] + \frac{1}{2}\alpha_{UU}\mathrm{Var}[U] + \frac{1}{2}\alpha_{VV}\mathrm{Var}[V] + \frac{1}{2}\alpha_{SS}\mathrm{Var}[S] \\ & + \frac{1}{2}\alpha_{S^*S^*}\mathrm{Var}[S^*] + \alpha_{U'V'}\mathrm{Cov}[U',V'] + \alpha_{UV}\mathrm{Cov}[U,V] + \alpha_{SS^*}\mathrm{Cov}[S,S^*] \\ & + \alpha_{SU'}\mathrm{Cov}[S,U'] + \alpha_{SV'}\mathrm{Cov}[S,V'] + \alpha_{S^*U'}\mathrm{Cov}[S^*,U'] + \alpha_{S^*V'}\mathrm{Cov}[S^*,V']\end{aligned}$$

where $\alpha_{XY} = \frac{\partial^2\Delta_2}{\partial X \partial Y}$ is the Taylor coefficient for variables X and Y evaluated at $(U,V,U',V',S,S^*) = (0,0,0,0,0,0)$. Since $\alpha_{U'U'} = \alpha_{V'V'} = \alpha_{UV} = \alpha_{SU'} = \alpha_{S^*V'} = \frac{\rho}{(1+\rho)^2}$, $\alpha_{UU} = \alpha_{VV} = \alpha_{U'V'} = \alpha_{SV'} = \alpha_{S^*U'} = \alpha_{SS^*} = -\frac{\rho}{(1+\rho)^2}$, $\alpha_{SS} = -\frac{1}{(1+\rho)^2}$, $\alpha_{S^*S^*} = -\frac{\rho^2}{(1+\rho)^2}$, $\mathrm{Var}[U] = \mathrm{Var}[U']$, $\mathrm{Var}[V] = \mathrm{Var}[V']$, and $\mathrm{Cov}[U,V] = \mathrm{Cov}[U',V']$, the above equation is simplified to

$$\mathrm{E}[\Delta_2] = \frac{2\rho\mathrm{Cov}[S-S^*,U'-V'] - \mathrm{Var}[S] - \rho^2\mathrm{Var}[S^*] - 2\rho\mathrm{Cov}[S,S^*]}{2(1+\rho)^2}.$$

Therefore, the condition for the A_2 allele being positively selected (i.e. invading the population initially fixed for A_1), is $\mathrm{E}[\Delta_2] > 0$ or

$$\frac{\mathrm{Cov}[S-S^*,U'-V']}{\mathrm{Var}[S]+\rho^2\mathrm{Var}[S^*]+2\rho\mathrm{Cov}[S,S^*]} > \frac{1}{2\rho}$$

assuming $(\mathrm{Var}[S] + \rho^2\mathrm{Var}[S^*])/\rho > -2\mathrm{Cov}[S,S^*]$. If $S_t^* = \delta S_t$, the above condition becomes

$$\Phi = \frac{\mathrm{Cov}[S,U'-V']}{\mathrm{Var}[S]} = \frac{\mathrm{Cov}[S,\log[K_F/K_R]]}{\mathrm{Var}[S]} > \frac{(1+\delta\rho)^2}{2(1-\delta)\rho}.$$

A similar result can be obtained for the condition for the A_1 allele to invade a population initially

fixed for A_2. The expected change in the number of A_1, n'_1/n_1, is obtained with a procedure identical to that leading to eq. (1) except that the relative fitness is flipped. Therefore

$$\frac{n_1'}{n_1} \approx \frac{K_F^{(t)}}{K_F^{(t-1)}+K_R^{(t-1)}}\frac{1}{1+S_t} + \frac{K_R^{(t)}}{K_F^{(t-1)}+K_R^{(t-1)}}\frac{1}{1+S_t^*} = \frac{(1+S_t^*)\mathrm{e}^{U'}+(1+S_t)\rho\mathrm{e}^{V'}}{(1+S_t)(1+S_t^*)(\mathrm{e}^{U}+\rho\mathrm{e}^{V})}$$

for $n_1 \ll n_2$, the dynamics of the rare A_1 allele depends on the expectation of

$$\Delta_{1,\mathrm{soft}} \equiv \log\left[\frac{n_1'}{n_1}\right] = \log\left[(1+S_t^*)\mathrm{e}^{U'} + (1+S)\rho\mathrm{e}^{V'}\right] - \log[(1+S)(1+S_t^*)(\mathrm{e}^{U}+\rho\mathrm{e}^{V})].$$

Applying the quadratic approximation used for obtain $\mathrm{E}[\Delta_2]$ above, one obtains

$$\mathrm{E}\left[\Delta_{1,\mathrm{soft}}\right] = \frac{(1+2\rho)\mathrm{Var}[S]+(2\rho+\rho^2)\mathrm{Var}[S^*]-2\rho\mathrm{Cov}[S,S^*]-2\rho\mathrm{Cov}[S-S^*,U'-V']}{2(1+\rho)^2}$$

The condition for the rare A_1 allele to increase in frequency is $\mathrm{E}\left[\Delta_{1,\mathrm{soft}}\right] > 0$ or

$$\frac{\mathrm{Cov}[S-S^*,U'-V']}{(1+2\rho)\mathrm{Var}[S]+(2\rho+\rho^2)\mathrm{Var}[S^*]-2\rho\mathrm{Cov}[S,S^*]} < \frac{1}{2\rho}.$$

If $S_t^* = \delta S_t$, the above condition becomes

$$\Phi < 1-\delta+\frac{(1+\delta\rho)^2}{2(1-\delta)\rho}.$$

With hard selection, the frequency change of A_2 when rare is again given by eq. (1). Therefore, the condition for A_2 invading the population fixed for A_1 ($\mathrm{E}[\Delta_2] > 0$ above) is no different from that for soft selection. However, because the carrying capacities of the field and refuge fixed for A_2 at generation t is given by $(1+S_t)K_F^{(t)}$ and $(1+S_t^*)K_R^{(t)}$ and $\tilde{N}_F^{(t)} = (1-r)((1+S_{t-1})K_F^{(t-1)} + (1+S_{t-1}^*)K_R^{(t-1)})$ and $\tilde{N}_R^{(t)} = r((1+S_{t-1})K_F^{(t-1)} + (1+S_{t-1}^*)K_R^{(t-1)})$ under hard selection, the opposite condition for A_1 invading A_2 is given by

$$\frac{n_1'}{n_1} = (1-r)\frac{(1+S_t)K_F^{(t)}}{\tilde{N}_F^{(t)}}\frac{1}{1+S_t} + r\frac{(1+S_t^*)K_R^{(t)}}{\tilde{N}_R^{(t)}}\frac{1}{1+S_t^*} = \frac{K_F^{(t)}+K_R^{(t)}}{(1+S_{t-1})K_F^{(t-1)}+(1+S_{t-1}^*)K_R^{(t-1)}} = \frac{e^{U'}+\rho\mathrm{e}^{V'}}{(1+S_{t-1})e^{U}+(1+S_{t-1}^*)\rho e^{V}}$$

Then,

$$\Delta_{1,\mathrm{hard}} \equiv \log\left[\frac{n_1'}{n_1}\right] = \log\left[\mathrm{e}^{U'}+\rho\mathrm{e}^{V'}\right] - \log[(1+S_{t-1})\mathrm{e}^{U} + (1+S_{t-1}^*)\rho\mathrm{e}^{V}].$$

Now, given that (U',V',S_t,S_t^*) and (U,V,S_{t-1},S_{t-1}^*) are independent samples of a joint distribution, the expectation of $-\Delta_{1,\mathrm{hard}}$ is exactly that of Δ_2 above. Therefore, the condition for the fixation of A_2 ($\mathrm{E}\left[\Delta_{1,\mathrm{hard}}\right] < 0$) is exactly the condition for positive selection on a rare A_2

($E[\Delta_2] > 0$).

Appendix B. Methods of stochastic simulation

Stochastic simulations of the single- and multi-locus eco-evolutionary models were implemented in Mathematica ver. 11.3. In both TP and LSA models, an individual has L loci that are arranged linearly with probability c for recombination between adjacent loci per generation. The simulation keeps track of two sets of lists: one containing the sets of haplotypes carried by non-zero individuals in the field and in the refuge ($\boldsymbol{H}_F$ and $\boldsymbol{H}_R$) and the other containing the corresponding numbers of individuals ($\boldsymbol{N}_F$ and $\boldsymbol{N}_R$).

In the TP model, generation t begins after the migration step (re-distribution of individuals produced in the previous generation into the field and refuge) is completed. Haplotype numbers are first updated by mutation and recombination. A Poisson variate with mean $NL\mu$, where N is the current size of a subpopulation at hand, is drawn as the total number of mutation events and each event is assigned to haplotypes and loci proportional to their current frequencies. Similarly, for $L > 1$, a Poisson variate with mean $N(L\text{-}1)c$ is drawn as the total number of recombination events in the subpopulation. For each recombination event, two haplotypes are chosen proportional to their frequencies and one of the L-1 intervals is chosen to be the position of crossing-over between the two haplotypes. Newly created or lost haplotypes during mutation and recombination steps are tracked by updating ($\boldsymbol{H}_F$, $\boldsymbol{H}_R$) and ($\boldsymbol{N}_F$, $\boldsymbol{N}_R$). Then, the updated lists are further updated in the step of reproduction according to their fitness. Under soft selection, the number of individuals with haplotype i born in the next generation is Poisson distributed with mean $W_i\tilde{N}_{F,i}^{(t)}K_F^{(t)}/\sum_j W_j\tilde{N}_{F,j}^{(t)}$ in the field and $\tilde{N}_{R,i}^{(t)}K_R^{(t)}/\sum_j \tilde{N}_{R,j}^{(t)}$ in the refuge, where W_i is the relative fitness of haplotype i and $\tilde{N}_{F,i}^{(t)}$ ($\tilde{N}_{R,i}^{(t)}$) is the number of haplotype i individuals in the field (refuge) after the haplotypes are updated by mutation and recombination. Under hard selection, the corresponding mean number in the field is $W_i\tilde{N}_{F,i}^{(t)}K_F^{(t)}/\sum_j \tilde{N}_{F,j}^{(t)}$. Finally, when there are N_F and N_R individuals in the field and refuge, Poisson variates with the mean $N_F m_{FR}$ ($N_R m_{RF}$) truncated at the upper bound N_F (N_R) is drawn to determine the numbers of migrants from field to refuge (from refuge to field). Migrants are chosen proportional to the haplotype frequencies and then merged with the residents of the destination. This completes a single-generation iteration in updating haplotype lists.

Simulation for the LSA model is similarly performed by tracking the haplotypes. Let l_i, m_i, and n_i

be the number of individuals with haplotype i (i = 1, …, 2^L) at the larval, subadult, and adult stages at time t. The following steps are taken to produce the corresponding numbers, l_i', m_i', and n_i', at time t+1. As in the TP model, bidirectional mutations with probability μ per locus and recombination with probability c for adjacent loci occur but in subadult and adult stages only. Recombination must occur among subadults and among adults only. The updated numbers of individuals by haplotypes in subadults and adults after mutations and recombination are m_i*, and n_i*. Next, the numbers of individuals by haplotypes in the next generation are obtained. l_i' is given by a Poisson number with mean $W_i^{(t)}(m_i^* + n_i^*)K_L^{(t)}/\sum_i(W_i^{(t)}(m_i^* + n_i^*))$ under soft selection and $W_i^{(t)}(m_i^* + n_i^*)K_L^{(t)}/\sum_i(m_i^* + n_i^*)$ under hard selection. m_i' and n_i' are given by Poisson numbers with mean $e_L l_i$ and $e_L e_S m_i$*, respectively.

Table 1. The long-term level of polymorphism in the multi-locus simulation

demography	selection mode	L	c	$\bar{H}$	P_{15}	P_{59}
$a_U = 0.2$, $a_V = b_U = b_V = 0.1$	neutral ($a_S = b_S = 0$)	1	-	0.0639	0.069	0.074
	soft selection, $\Phi = 1$ ($a_S = 0.1$, $b_S = 0$)	1	-	0.132	0.15	0.16
		4	10^{-4}	0.0960	0.099	0.12
		4	10^{-3}	0.103	0.12	0.12
		4	0.01	0.0977	0.11	0.12
		4	0.1	0.136	0.11	0.14
		10	10^{-4}	0.080	0.087	0.095
		10	10^{-3}	0.102	0.12	0.12
		10	0.01	0.109	0.15	0.10
		10	0.1	0.107	0.13	0.12
$a_U = 0.3$, $a_V = 0.05$, $b_U = b_V = 0.1$	neutral ($a_S = b_S = 0$)	1	-	0.0633	0.081	0.074
	soft selection, $\Phi = 1$ ($a_S = 0.2$, $b_S = 0.1$)	1	-	0.177	0.24	0.18
		4	10^{-4}	0.162	0.19	0.19
		4	10^{-3}	0.130	0.21	0.11
		4	0.01	0.168	0.22	0.18
		4	0.1	0.183	0.25	0.19
		10	10^{-4}	0.120	0.14	0.14
		10	10^{-3}	0.141	0.17	0.17
		10	0.01	0.0934	0.076	0.14
		10	0.1	0.105	0.093	0.15
$a_U = 0.05$, $a_V = b_U = b_V = 0$	neutral ($a_S = b_S = 0$)	1	-	0.0660	0.085	0.063
	soft selection, $\Phi = 1$ ($a_S = 0.05$, $b_S = 0$)	1	-	0.0816	0.11	0.081
		10	10^{-4}	0.0728	0.085	0.079
		10	10^{-3}	0.0982	0.12	0.11
		10	0.01	0.0953	0.12	0.096
		10	0.1	0.0952	0.13	0.092

- Simulations were run for 5×10^5, 2×10^5, and 10^5 generations with L = 1, 4, and 10, respectively.
- Other parameters: $K_{R0} = K_{F0} = 1{,}000$ (ρ=1), $\mu = 2\times10^{-5}$.

FIGURE LEGENDS

Figure 1. Schematic diagrams of population models. **A**. Two-patch (TP) model assumes a population of haploid individuals occupying a field, where the mutant allele A_2 has selective advantage $1 + S_t$ in generation t, and a refuge, where the advantage is $1 + S_t^*$. Each generation starts with young individuals that either migrated across or stayed in subpopulations after being reproduced in the previous generation. They also undergo mutation and recombination before selection starts. Then, they undergo selection such that the total number of reproducing individual is constrained to the carrying capacity $K_F^{(t)}$ for the field and $K_R^{(t)}$ for the refuge assuming soft selection. In case of hard selection the carrying capcities are effectively $(1 + qS_t)K_F^{(t)}$ and $(1 + qS_t^*)K_F^{(t)}$. **B**. In the larval-subadult-adult (LSA) model, at each time step haploid individuals move from larval to subadult stage with probability e_L and from subadult to adult stage with probability e_S. Reproduction, followed immediately by mutation and recombination, occurs only in the subadult and adult stages. A new larval subpopulation at time t starts with individuals produced at time t-1. At the end of time interval t, $K_L^{(t)}$individuals remain in the larval stage before moving to the next stage.

Figure 2. Distribution of A_2 allele frequencies (p) at the 5,000th generation in the stochastic simulations of soft (A, B) and hard (C, D) selection in the TP model. The initial frequency of A_2 is 0.5 in both field and refuge. Reproduction occurred while $K_F^{(t)}$, $K_F^{(t)}$ and S_t varied stochastically without (A, C) or with (B, D) genetic drift and bidirectional mutation ($\mu = 5\times10^{-5}$). Proportions of simulation replicates (out of 1000) with the frequencies in the interval $0 \le p < 0.1$, $0.1 \le p < 0.2$, …, $0.8 \le p < 0.9$, and $0.9 \le p \le 1$ were plotted and connected by lines. Fluctuations of population sizes are given by $K_{R0} = K_{F0} = 1000$ $(\rho = 1), a_U = 0.3, b_U = 0.1, a_V = 0.05, b_V = 0.1$. Parameters of fluctuation were chosen to yield Φ = 0.3, 0.5, 0.77, 1, 1.2, 1.5, and 2: (a_S, b_S) = (0.07, 0.23), (0.1, 0.2), (0.14, 0.16), (0.2, 0.1), (0.2, 0.04), (0.15, 0.05), and (0.1, 0.05). In the case of hard selection with Φ = 1 and no genetic drift (panel C), simulation ran only up to 2,500 generations: longer simulations or larger Φ resulted in all replicates with final frequencies > 0.9. The results connected by dashed lines in B and D represent the cases of neutral evolution ($a_S = b_S = 0$).

Figure 3. Simulation results for non-zero values of δ. For a given parameter set simulation started with the A_1 allele fixed in the population and ran for 10^5 generations with recurrent bidirectional mutations and soft (left) or hard (right) selection. Four different outcomes regarding the overall behavior of the A_2 allele (balanced, lost, fixed, neutral) were marked by different symbols and plotted as functions of δ and Φ. The demographic parameters are identical to those in Figure 1. Five different values of δ (-0.3, -0.1, 0.1, 0.3, and 0.3) and ten different values of Φ were chosen: 0.246, 0.5, 0.8, 1, 1.25, 1.5, 1.786, 2.027, 2.5 and 2.94: (a_S, b_S) = (0.05, 0.22), (0.1, 0.2), (0.2, 0.15), (0.2, 0.1), (0.2, 0), (0.15, 0.05), (0.14, 0), (0.12, 0.02), (0.1, 0) and (0.085, 0). Gray and dashed curves plot $\Phi = \frac{(1+\delta\rho)^2}{2(1-\delta)\rho}$ and $\Phi = \frac{1+\delta^2}{1-\delta} + \frac{(1+\delta\rho)^2}{2(1-\delta)\rho}$ with $\rho = 1$.

Figure 4. Changes of mutant (A_2) allele frequencies and subpopulation sizes (A. TP model, blue: $N_F^{(t)}$, green: $N_R^{(t)}$; B. LSA model, blue: larval, green: subadult, gray: adult population) in the multi-locus simulations of hard selection. Allele frequency trajectories of 8 loci were plotted in different colors. Parameters: A (TP model): L = 8, c = 0.02, $K_{R0} = K_{F0}$ = 1000, μ = 2×10^{-5}, and Φ = 1 (a_S = 0.15, b_S = 0, a_U = 0.15, a_V = 0, b_U =0, b_V = 0.1). B (LSA model): L = 8, c = 0.02, K_{L0} = 5000, μ = 2×10^{-5}, and Φ' = 1 (a_S = 0.1, b_S = 0.1, a_L = 0.2, b_L= 0.1).

Figure 5. Mean fixation time ($\bar{T}$) with varying number of loci (A; L = 1, 2, 4, and 8) and the coefficient of variation in fixation times with L = 8 in the multi-locus simulation of the TP model. Error bars show two times the standard errors. A total of 100 replicates of simulations were run under soft selection with Φ = 2 (cyan; a_S = 0.1, b_S = 0, a_U = 0.25, b_U = a_V = b_V = 0.05), hard selection with Φ = 1 (brown; a_S = 0.1, b_S = 0, a_U = 0.1, a_V = 0, b_U = b_V = 0.1), or hard selection with Φ = 0.75 (orange; a_S = 0.2, b_S = 0, a_U = 0.25, a_V = 0.1, b_U = b_V = 0.05). Other parameters are identical to those in Figure 4A.

 Click here to access/download;Figure;Fig1A.tif

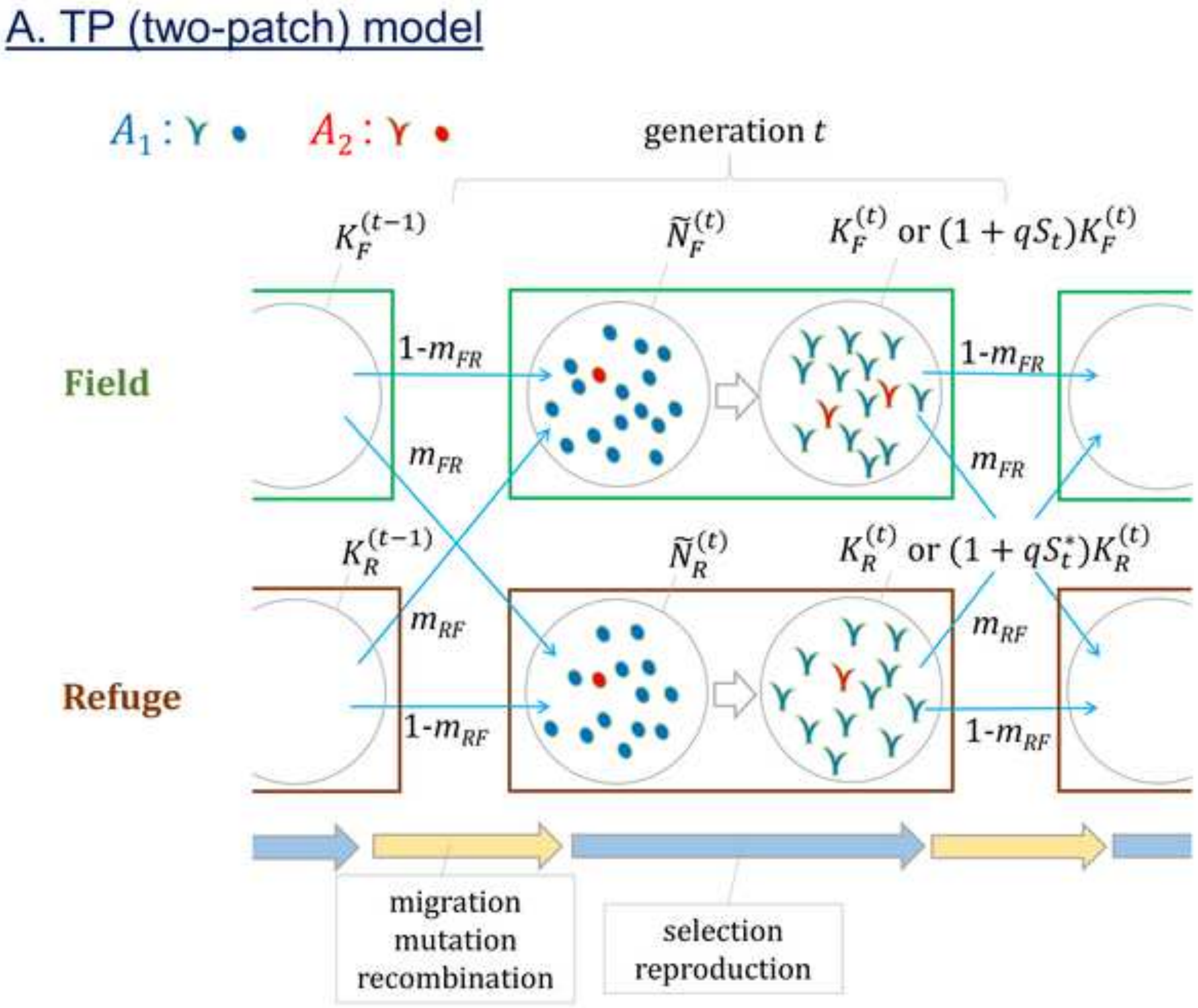

Click here to access/download;Figure;Fig1B.tif

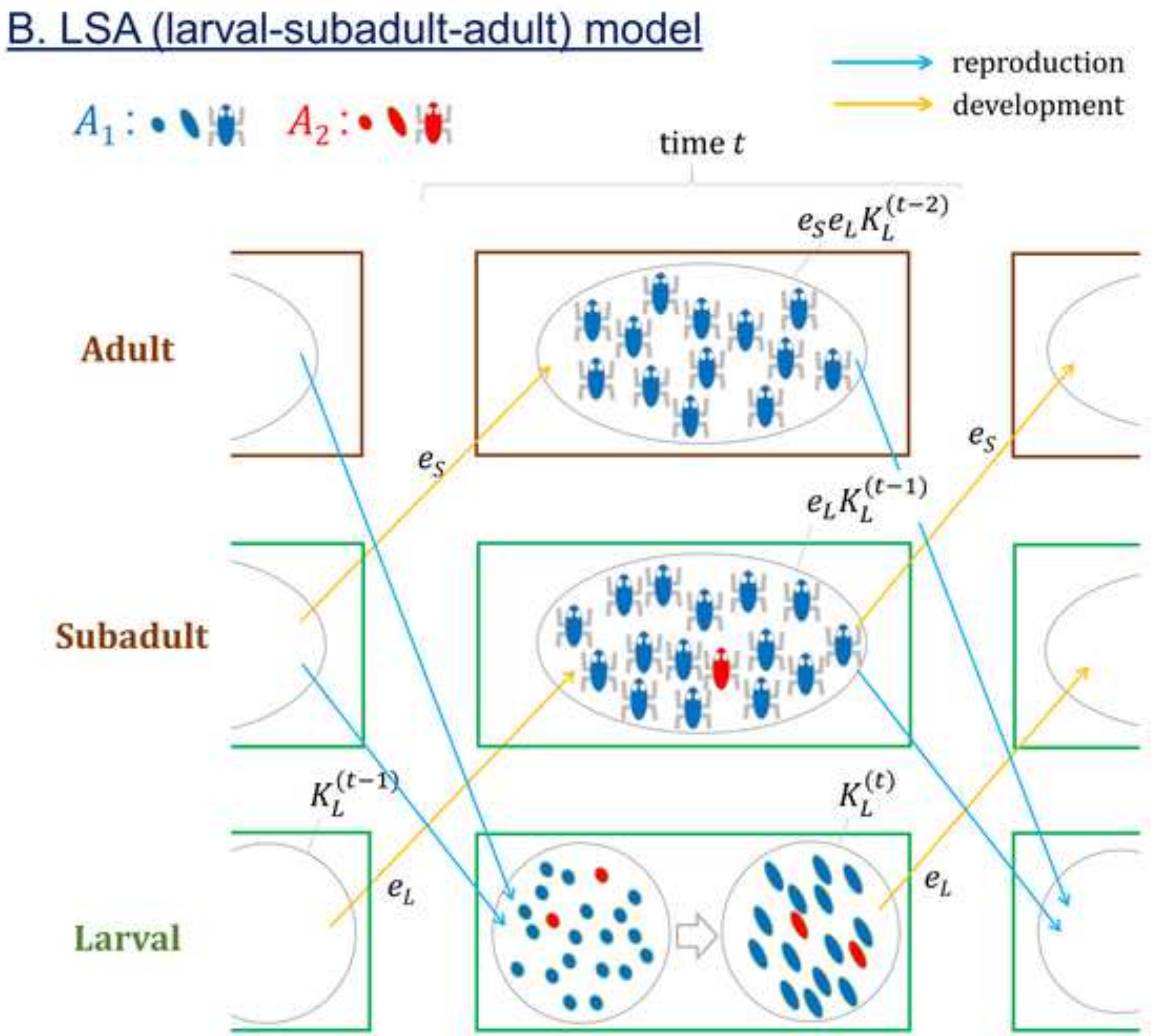

Figure 2

Click here to access/download;Figure;Fig2.tif

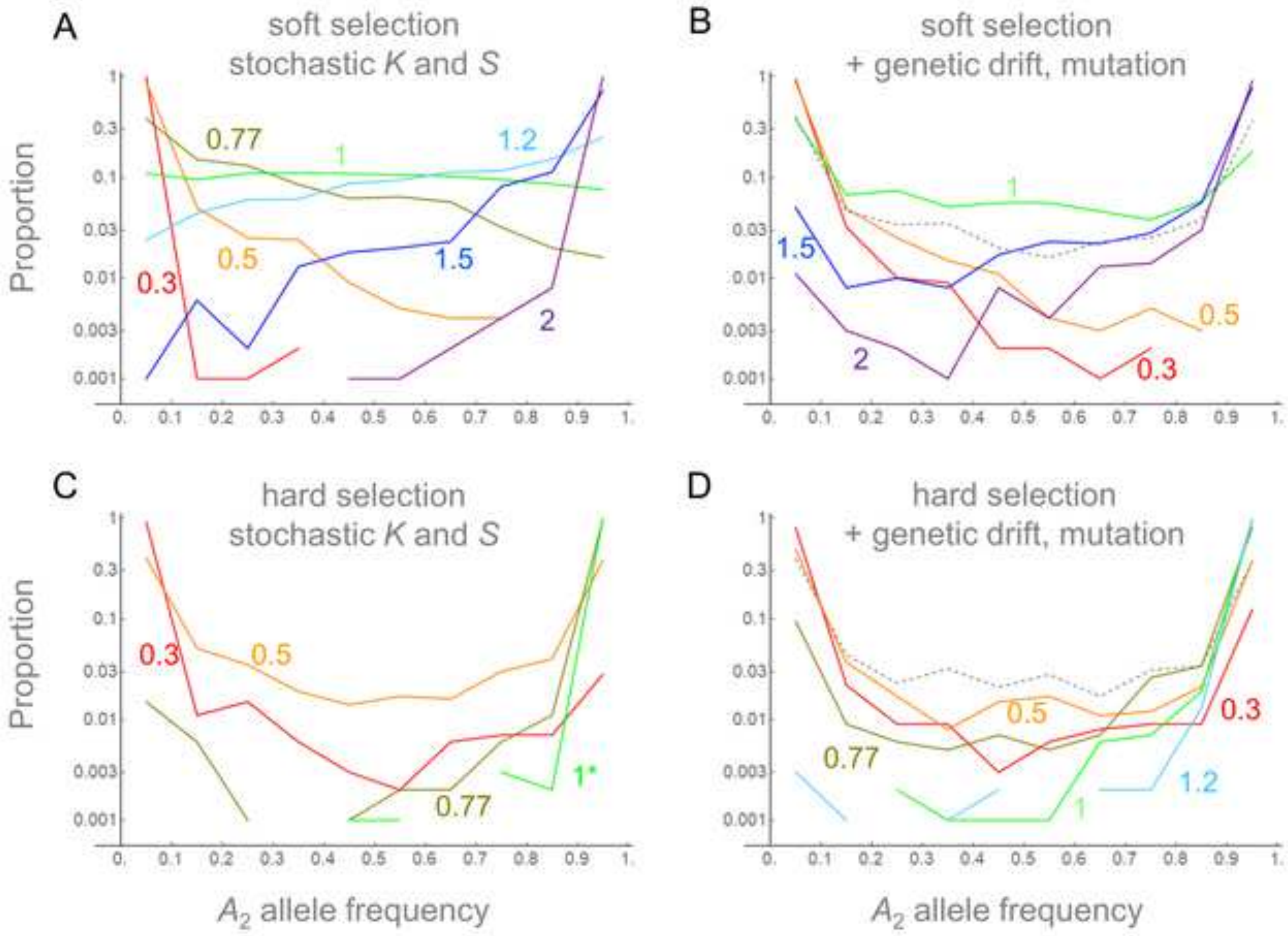

Figure 3

Click here to access/download;Figure;Fig3.tif

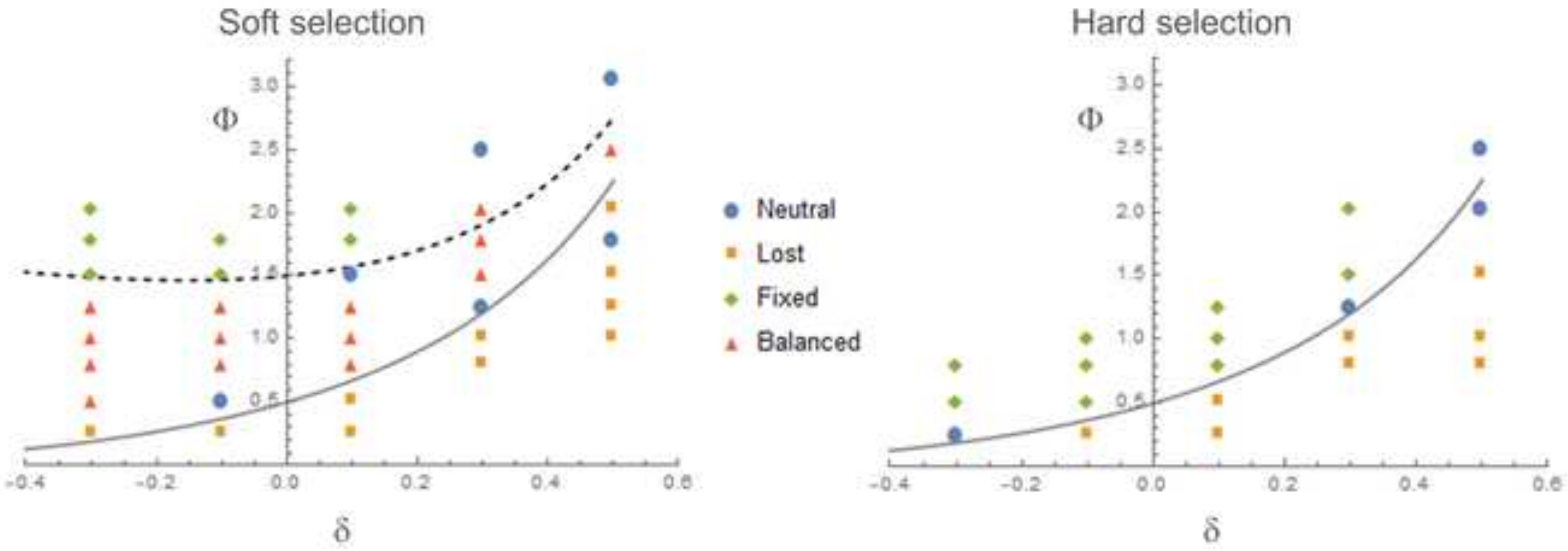

Figure 4

Click here to access/download;Figure;Fig4.tif

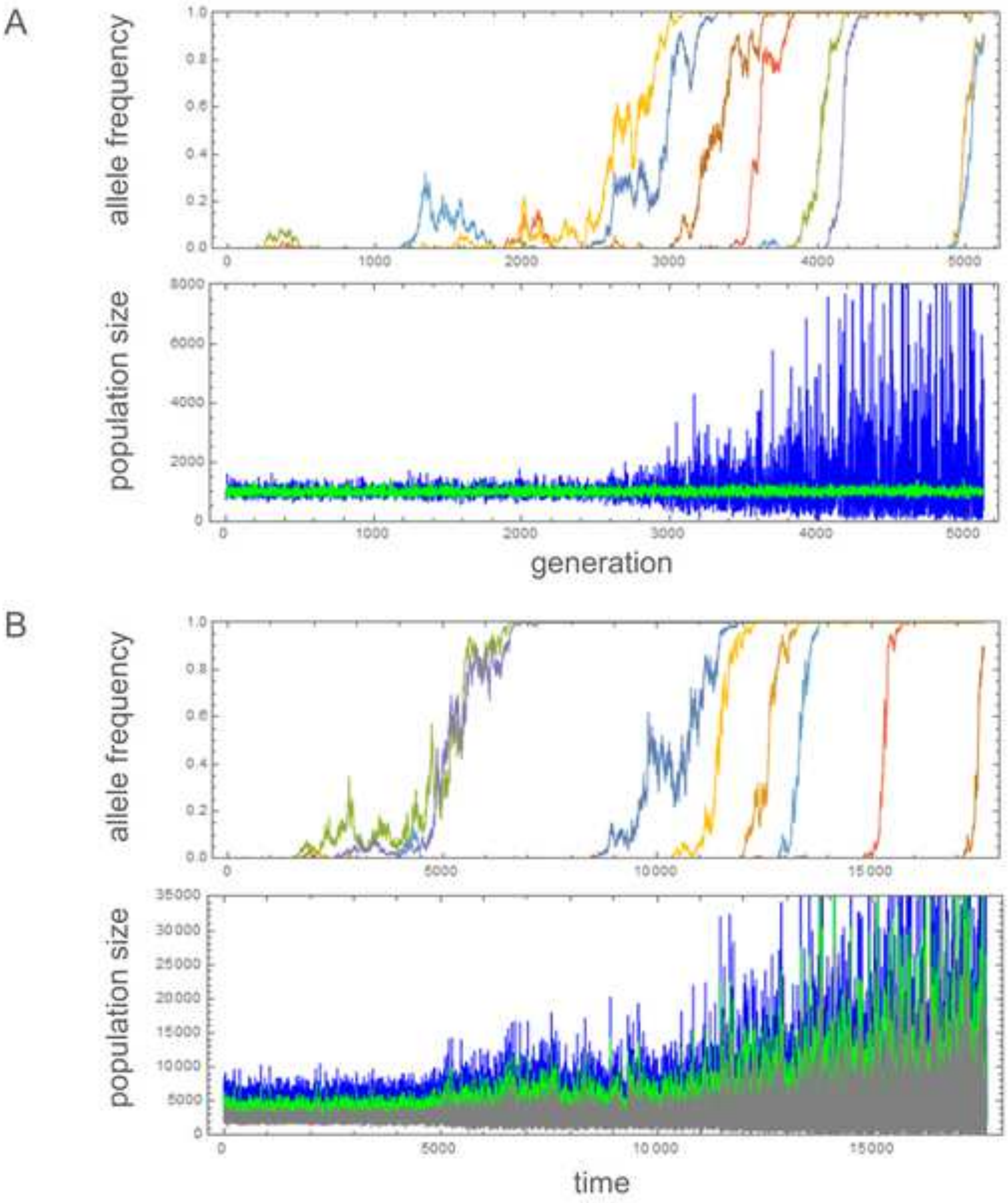

Figure 5

Click here to access/download;Figure;Fig5.tif

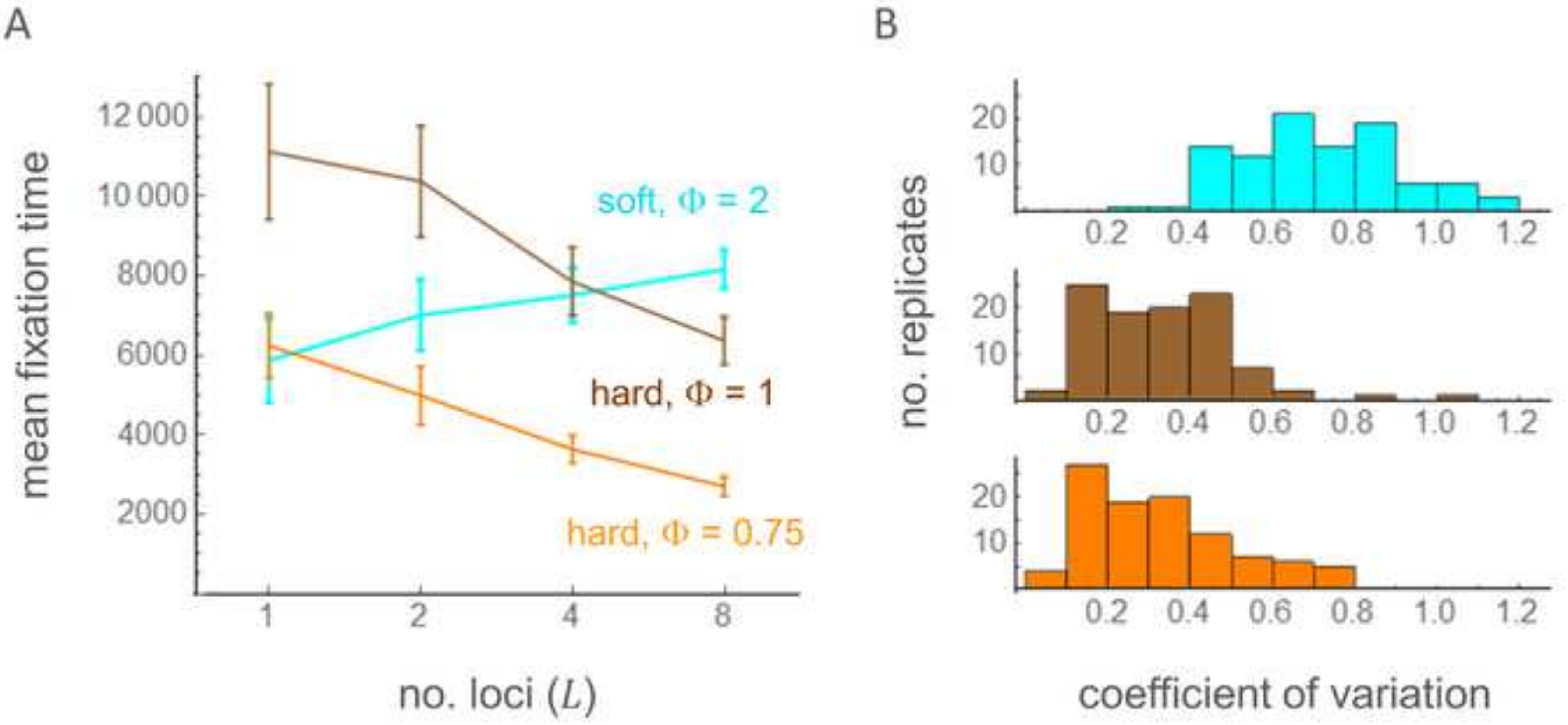

Click here to access/download
Supporting Information
SuppMaterial0221.pdf

Noncovarying storage effect: balancing and positive directional selection on mutant alleles that amplify random fitness and demographic fluctuations

Yuseob Kim

Division of EcoScience, Ewha Womans University, Seoul, Korea 03760

yuseob@ewha.ac.kr

Running head: Eco-evolutionary fluctuation dynamics

Keywords: fluctuation, storage effect, population size, positive feedback, eco-evolutionary

Correspondence:

Yuseob Kim, Science Hall B308, Ewhayeodae-gil 52, Seodaemun-gu, Seoul, Korea 03760

Tel: +82 10 5138 5175

ABSTRACT

Temporally variable environments in natural populations generate fluctuations in both the population size and the fitness effects of mutant alleles. The theory of storage effect, a species/allelic diversity-promoting mechanism discovered in ecology, predicts that rare mutants with fluctuating fitness can be positively selected and then maintained in balanced polymorphism if one part of the population, the 'field', is~~ is subdivided into two parts that are respectively~~ exposed to and the other, the 'refuge', is~~and~~ protected from fluctuating selection~~environment~~. Recent study found that oscillation in population size modifies the storage effect such that positive selection on a rare mutant occurs if its fitness fluctuation correlates with oscillation in the size of the field. By this new version of storage effect, mutants with even smaller geometric mean fitness than the wild-type can be selected to intermediate frequencies and remain in balanced polymorphism or reach fixation.~~, under pre-exisiting oscillation in population size, the mutant is positively selected to fixation if its fitness change correlates with the rate of population growth, which further amplifies population size oscillation.~~ To further understand these eco-evolutionary dynamics and ~~elucidate~~ test their generality in natural populations, this study built more realistic models that assume randomly, not cyclically, fluctuating selection and common demographic features, including heterogeneous ecological patches or an age-structured population. Mathematical analysis elucidated~~revealed~~ that whether balanced polymorphism or fixation occurs depends on the relative magnitudes of demographic versus fitness fluctuations and that both results require incomplete correlation in population size between the field and refuge. Therefore this novel evolutionary force is~~ generated when the size of the subpopulation subject to weak selection does not covary with that of the other subpopulation. Simulations showed that this~~ called 'noncovarying storage effect (NSE)'~~ is robust to various perturbations in the model~~. Multi-locus simulations revealed that oscillatory polymorphism ~~at many loci~~ can be maintained simultaneously or reach fixations at many loci. The latter occurs in a wider range of parameters and, if the fitness effects of mutations change the mean absolute fitness of the population, leads to~~ maintained and that~~ positive feedback between demography and selection ~~can accelerate the sequential fixations of fluctuation-amplifying mutations and thus lead to~~that causes a drastic amplification of population size fluctuation. These results suggest that the ~~noncovarying storage effect~~NSE is a potentially prevalent evolutionary force in nature for maintaining genetic variation ~~and~~ or causing large demographic fluctuations.

ARTICLE SUMMARY

Natural populations experience rapid environmental changes that cause fluctuations in the fitness of genetic variants and population size. This study investigates how evolutionary changes due to fitness differences interact with concurrent demographic changes. The theory of the storage effect, which is a mechanism for promoting genetic diversity, was extended to add random population size fluctuation with heterogeneous environmental patches or in a population divided into different age classes. A novel evolutionary force that either increases genetic variation or drives evolution toward further amplification of population size fluctuation, an unconventional contributor to large demographic fluctuations in nature, was identified and analyzed.

INTRODUCTION

Natural populations experience temporal changes in abiotic and biotic environments. A randomly variable environment is expected to produce fluctuations in the fitness effects of non-neutral or phenotype-changing alleles segregating in the population. If the magnitude of fluctuation in the relative fitness is large, evolutionary changes may occur rapidly at the pace of short-term ecological/demographic fluctuations. Recent developments in evolutionary biology have revealed that such rapid evolutionary changes are widespread (Bell 2010; Thompson 2013; Messer *et al.* 2016; Lynch *et al.* 2024). Temporally variable environments should also generate fluctuations in various aspects of demography, most importantly the number of reproducing individuals (i.e., population size). One may then ask whether mutual interaction between demographic and selective fluctuations creates qualitatively novel eco-evolutionary dynamics that cannot arise if one of the demographic or population genetic variables is held constant.

~~Considering that a fluctuation in population size reflects a fluctuation in the reproductive success of individuals and that long-term reproductive success is maximized if its variance in the offspring number over time is minimized (Slatkin 1974; Gillespie 1977; Tuljapurkar 1982), one may predict that a population will evolve to reduce size fluctuation as much as possible (Pfister 1998). For example, a variant increasing the egg size rather than the egg number at reproduction when plentiful resources are available may be positively selected as the offspring can survive better during an unfavorable period. Then, when a large fluctuation in population density, such as mast seeding or insect outbreak, does occur despite the advantage of a small temporal variance in offspring number, this may be seen as a non-adaptive response of the population to a heavy fluctuation of the biotic and/or abiotic environment (Myers 1988; Dwyer *et al.* 2004; White 2008; Bogdziewicz *et al.* 2020). However, recent studies suggested that a species may evolve to undergo a larger fluctuation in its population size. Liu *et al.* (2019) found that a reproductive strategy increasing the temporal variance of offspring number can be advantageous in populations reproducing in overlapping generations under various patterns of environmental fluctuations. Kim (2023) showed that a mutation amplifying the cyclic fluctuation of population size can be positively selected if this population is partially protected from the cyclical selection (i.e. fitness fluctuation occurs only in a subset of the population).~~

One of major questions in the population genetics of fluctuating selection is whether different alleles that are favored in different time points can coexist in polymorphism. The prevalent conclusion has been that polymorphism is more difficult to be maintained under temporal

fluctuation in selection than without fluctuation, as the allele under selection is pushed closer to frequency 0 or 1, after which it is more readily lost or fixed in the population by genetic drift (Hedrick et al. 1976; Hedrick 1986). However, recent studies suggested that temporal fluctuating selection can turn into balancing selection, an evolutionary force maintaining polymorphism at a level higher than expected under neutral evolution. It was shown that such balancing selection occurs if a population is partially protected from the cyclical selection, namely if fitness fluctuation occurs only in a subset of the population (Chesson & Warner 1981; Ellner & Hairston 1994; Reinhold 2000; Gulisija & Kim 2015; Gulisija *et al.* 2016; Bertram & Masel 2019; Yamamichi *et al.* 2023). Partial protection from fluctuating selection commonly occurs in many species. In spatially structured species the amplitude of fluctuation in selective pressure is likely to be heterogeneous over the geographic range. In plants and animals that have their life cycles divided into several stages and reproduce in overlapping generations, selective pressure is unlikely to act uniformly over individuals of all stages or ages. For example, if a trait under fluctuating selection is expressed in the insect larval stage, alleles affecting this trait currently carried by adults are not visible to the selection. Similarly, alleles affecting traits that appear after germination remain neutral in the seed bank of a plant species. Such a subset of the population protected from fluctuating selection may be called a "refuge." The presence of a refuge of various kinds was found to generate the advantage of rare alleles, an eco-evolutionary process~~ form of balancing selection~~ commonly referred to as the storage effect (Chesson 1983; Ellner & Hairston 1994; Reinhold 2000; Gulisija & Kim 2015; Gulisija *et al.* 2016; Bertram & Masel 2019; Yamamichi *et al.* 2023). A~~, as the~~ refuge acts to temporarily store the variants that otherwise will be removed from the population during unfavorable periods. More importantly, a refuge dampens fluctuation in the mean absolute fitness (per-capita rate of reproduction) of individuals carrying the rare allele under selection, which increases the geometric mean fitness of the rare allele above that of the common allele (Kim 2023). The storage effect was first theorized in community ecology, shown to arise not only with partial protection from fluctuation provided by a refuge but with general covariance between environment and competition, and is now one of the most important ecological theories for explaining the stable coexistence of species (Chesson & Warner 1981; Chesson 1994; Barabás *et al.* 2018). Imported into population genetics, it was recently proposed as one of a few mechanisms that may produce the genome-wide occurrence of oscillatory polymorphism, as observed in North American *Drosophila melanogaster* and other species, which appear to be maintained under fluctuating environments (Bergland et al. 2014; Park and Kim 2019; Johnson et al. 2023).

However, Kim (2023) showed that if the oscillation of population size is added to the model of

storage effect a variant whose fitness also oscillates in the same directions can be either maintained in balanced polymorphism or positively selected at all frequencies until it reaches fixation. Such a variant may be called a fitness-amplifying allele because the relative fitness of an individual carrying this allele is high when the absolute fitness of all individuals is also high due to temporary population growth. Mathematical analysis showed that whether balancing or directional selection occurs on the~~this~~ fitness-amplifying allele depends on the relative strengths of fitness versus population size fluctuations. Previous studies on balancing selection by the storage effect in a constant-sized population required the coexisting variants to ~~have~~ confer identical geometric mean fitness to the carriers. Such variants, exhibiting the geometric-mean-preserving fitness fluctuation (GMF) as defined in Kim (2023), are however selected either to loss or fixation in the presence of oscillation in the size of population outside the refuge. One may therefore regard the population size oscillation as a factor that destroys the potential of storage effect to generate balanced polymorphism. However, this ~~when the~~ population size ~~was allowed to~~ oscillation~~, it~~ led to balancing~~negative frequency-dependent~~ selection on a different type of mutants — alleles conferring~~with even~~ smaller geometric mean but identical arithmetic mean fitness compared to~~than~~ the wild-type ~~allele~~. Since such AMF (arithmetic-mean-preserving fitness fluctuation) mutations, which are deleterious in a simple population (without a refuge), are probably more likely to occur in the genome than the GMF mutations, this version of storage effect modified by demographic fluctuation might be equally important or even more important in generating balanced polymorphism in nature. It was also shown that ~~positive directional selection on such~~ AMF variants are positively selected all the way to fixation~~, which cannot arise in the previous models of the storage effect, occurs~~ if demographic fluctuation is relatively stronger than fitness fluctuation. Furthermore, Kim (2023) found that, under a weak regulation of population density under which the population size can over- or undershoot the carrying capacity, the positive selection leads to the amplification of oscillation in mean absolute fitness, thus population size, due to the fixation of the fitness-amplifying allele. In summary, the addition of population size fluctuation~~, either externally imposed by ecology or due to the segregation of fitness-amplifying mutants, in the model~~ was found to generate novel eco-evolutionary dynamics not predicted by the conventional model of the storage effect.

The generality of novel eco-evolutionary dynamics discovered in Kim (2023) may however be questioned since the major result was obtained from the simulation of cyclically (seasonally) and symmetrically changing environments under which the population size and fitness oscillations are fully correlated. Although a mathematical analysis found general conditions for determining

which evolutionary outcome (the loss, balanced polymorphism, or fixation of the fitness-amplifying allele) is expected, this result was based on the model of only two alternating seasons with fixed sizes. Therefore further investigation using more realistic models is needed to obtain better theoretical understanding and to find out whether the novel eco-evolutionary dynamics can arise in a substantially wider range of biological settings.

This study broadens the scope of investigation in the following directions. First, mathematical models assuming random, rather than deterministic and symmetrically seasonal, fluctuations in the environment were built and analyzed. A model with seasonal fluctuation is suitable for cases in which one cycle of environmental changes spans multiple generations. However, for many species, one generation is not shorter than a seasonal cycle. Random fluctuation of carrying capacities and the direction/strength of selection over generations, with a varying degree of correlation between these fluctuations, is probably relevant to a wider range of species including plants that exhibit mast seeding. Second, new models of population structures and inter-deme differences in selection intensity were investigated. Whereas Kim (2023) focused on a population with a seed bank (Ellner and Hairston 1994; Turelli *et al.* 2001; Gremer and Venable 2014) in which the mutant is completely protected from selection in the refuge, this study uses a model (the two-patch or TP model) that assumes two demes or ecological patches, both reproducing and undergoing changes in the carrying capacity, under general heterogeneity in selection intensity. Selection in the refuge may not be completely neutral but simply weaker than that in the field. This TP model was also analyzed extensively by simulation to test its robustness against perturbations. Another model (the larval-subadult-adult or LSA model) assuming a population with an age structure, in which the life-cycle is divided into the larval (juvenile), subadult (young adult), and (old) adult stages, was also explored by simulation. Third, for both TP and LSA models, multi-locus stochastic simulations were performed. Simulations showed that mutant alleles causing wider fluctuation in offspring number can reach fixation at multiple loci in a wide range of conditions. These fixations were found to occur at faster rates with increasing numbers of loci, as fluctuating population size and fluctuating selection reinforced each other in positive feedback.

As will be clarified in the mathematical analysis, a key ingredient for this novel eco-evolutionary dynamics is the presence of a refuge whose size (or carrying capacity) remains relatively invariant or does not covary with the size of the rest of the population. One may therefore call this evolutionary mechanism the "noncovarying storage effect (NSE)". The result suggests that the ~~noncovarying storage effect~~NSE arising under the general condition of heterogeneous fluctuating selection in structured populations likely contributes to maintaining non-neutral genetic

variation ~~and~~ or to increasing demographic variability. Analytical and simulation results for the TP model will be first presented, followed by simulation results for the LSA model.

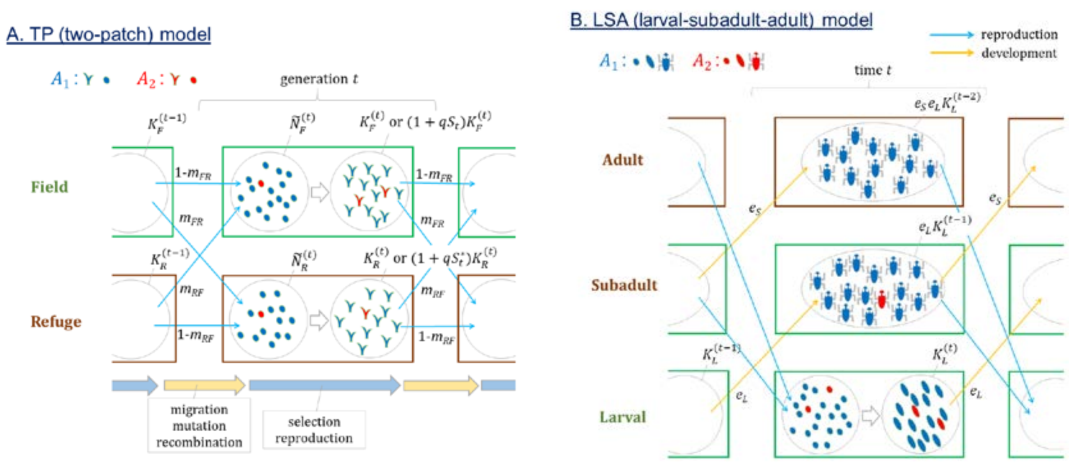

Figure 1. Schematic diagrams of population models. **A.** Two-patch (TP) model assumes a population of haploid individuals occupying a field, where the mutant allele A_2 has selective advantage $1 + S_t$ in generation t, and a refuge, where the advantage is $1 + S_t^*$. Each generation starts with young individuals that either migrated across or stayed in subpopulations after being reproduced in the previous generation. They also undergo mutation and recombination before selection starts. Then, they undergo selection such that the total number of reproducing individual is constrained to the carrying capacity $K_F^{(t)}$ for the field and $K_R^{(t)}$ for the refuge assuming soft selection. In case of hard selection the carrying capcities are effectively $(1 + qS_t)K_F^{(t)}$ and $(1 + qS_t^*)K_F^{(t)}$. **B.** In the larval-subadult-adult (LSA) model, at each time step haploid individuals move from larval to subadult stage with probability e_L and from subadult to adult stage with probability e_S. Reproduction, followed immediately by mutation and ~~reproduction~~recombination, occurs only in the subadult and adult stages. A new larval subpopulation at time t starts with individuals produced at time t-1. At the end of time interval t, $K_L^{(t)}$individuals remain in the larval stage before moving to the next stage.

Two-patch (TP) model

Consider a haploid population that reproduces in discrete generations. The outline of the life cycle is illustrated in Fig. 1A. The population is divided into two subpopulations or patches that are subject to different levels and/or phases of fluctuating selection (thus two-patch or TP model). For convenience, these subpopulations will be called the field and the refuge following the

terminology of Kim (2023). The latter refers to a subpopulation where selection on a mutant allele is weaker. It is assumed that the population is initially fixed for the ancestral A_1 allele at a locus. Then, a new mutant allele A_2, subject to fluctuating selection arises. The fitness of an individual carrying A_2 relative to A_1 is given by $1 + S_t$ in the field and $1 + S_t^*$ in the refuge, where S_t and S_t^* are drawn each generation from a joint distribution with $\mathrm{E}[S_t] = \mathrm{E}[S_t^*] = 0$. While the specific form of the distribution is needed for simulation (below), for deriving analytic solution in the next section only the mean, variance, and covariance of the distribution will be needed.~~, $\mathrm{Var}[S_t] > 0$, and $\mathrm{Var}[S_t^*] \geq 0$. Notably,~~It should be noted that this study considers only AMF mutations; while the arithmetic mean of the fitness of A_2 over time is 1, its geometric mean is less than 1, in both subpopulations. Natural selection is thus expected to eliminate such an allele from a simple panmictic (unstructured) population.

A fluctuating environment causes fluctuations in the carrying capacities of both the field and refuge, which are given by $K_F^{(t)}$ and $K_R^{(t)}$ respectively for generation t. The carrying capacity is defined as the expected number of (reproductive) individuals at the end of each generation. It is assumed that the carrying capacities of both subpopulations depend on common environmental factors that fluctuate randomly over time but that subpopulations may differ in the strengths of the dependence. The sizes of subpopulations, $N_F^{(t)}$ and $N_R^{(t)}$, after the reproduction step in generation t, may or ma~~n~~y not match the corresponding carrying capacities depending on the strength of population density regulation (see below). Before a new generation starts, each individual in the field migrates to the refuge or remains in the field with probability m_{FR} and 1 - m_{FR}. Similarly, each individual in the refuge either migrates or not with probability m_{RF} and 1 - m_{RF}. Next, the size of the field (refuge) changes from $\tilde{N}_F^{(t)}$ ($\tilde{N}_R^{(t)}$) at the beginning of generation t to $N_F^{(t)}$ ($N_R^{(t)}$) at the end, where $\mathrm{E}[\tilde{N}_F^{(t)}] = (1 - m_{FR})N_F^{(t-1)} + m_{RF}N_R^{(t-1)}$ and $\mathrm{E}[\tilde{N}_R^{(t)}] = m_{FR}N_F^{(t-1)} + (1 - m_{RF})N_R^{(t-1)}$.

Let $n_{1\mathrm{F}}$ and $n_{2\mathrm{F}}$ be the numbers of individuals in the field carrying A_1 and A_2 alleles at the beginning (before the reproduction step) of generation t (thus $n_{1\mathrm{F}} + n_{2\mathrm{F}} = \tilde{N}_F^{(t)}$). In the reproduction step, two different assumptions can be made regarding the effect of the mean fitness of individuals on the total population size. When the allele with higher or lower fitness increases in frequency, the total population size may not change due to an ecological constraint. In other words, the absolute fitness of the allele is frequency-dependent while its relative fitness remains constant. Conversely the mutant allele may increase or decrease the absolute reproductive output of the carriers regardless of its frequency such that the population size can increase over or decrease below the

carrying capacity, which is now interpreted as the expected population size assuming that all individuals carry the ancestral (A_1) allele. For convenience, these two modes of natural selection will be simply referred to as soft and hard selection, respectively, following the terminology in Wallace (1975). First, under soft selection,~~ very strong density regulation occurs so that~~ the final size of the field is constrained to $K_F^{(t)}$. The number of progeny born to each parent is given by $WK_F^{(t)}/\bar{N}_F^{(t)}$, where the fitness W is 1 for A_1 and $1+S_t$ for A_2, and $\bar{N}_F^{(t)} = n_{1\mathrm{F}} + (1+S_t)n_{2\mathrm{F}}$. Second, under hard selection, fitness-changing mutations lead to changes in not only allele frequencies but also in the population size. To this effect, the number of progeny per parent is now $WK_F^{(t)}/\tilde{N}_F^{(t)}$. Namely, when $q = n_{2\mathrm{F}}/(n_{1\mathrm{F}} + n_{2\mathrm{F}})$ is the relative frequency of A_2 at the beginning of a generation, the size of the field is expected to reach $(1+qS_t)K_F^{(t)}$, which becomes the effective carrying capacity of the field under hard selection. Similarly, reproduction in the refuge occurs either with soft or hard selection, where subscript F in the above expressions is replaced by R and W is 1 for A_1 and $1+S_t^*$ for A_2. The model so far described only the expected changes in the numbers of A_1- and A_2-carrying individuals~~ without genetic drift~~. However, as reproduction in a finite-sized population was implicitly assumed, how genetic drift occurs need to be specified for stochastic simulation. To set the variance of offspring number similar to that in the haploid version of Wright-Fisher model,~~When a finite population size is assumed, as in simulation below,~~ each parent in both subpopulations is modeled to produce a Poisson number of offspring with the mean specified above~~, which approximates genetic drift in the Wright-Fisher model~~.

Finally, how the environment fluctuates over time and how demographic and selective parameters — $K_F^{(t)}$, $K_R^{(t)}$, S_t, and S_t^* — respond need to be specified. The environmental condition is assumed to fluctuate randomly in each generation, therefore with no temporal autocorrelation. More specifically, fluctuations occur such that $K_F^{(t)} = K_{F0}\mathrm{e}^{U_t}$ and $K_R^{(t)} = K_{R0}\mathrm{e}^{V_t}$ where U_t and V_t~~—~~, together with S_t and S_t^*, are drawn each generation from a joint normal distribution satisfying $\mathrm{E}[U_t] = \mathrm{E}[V_t] = \mathrm{E}[S_t] = \mathrm{E}[S_t^*] = 0$. The oscillation of carrying capacities is therefore symmetrical in the log scale, which is generally predicted in demographic models and observed in nature (Tuljapurkar & Orzack 1980; Myers 1988). Various assumptions can be made regarding the variance-covariance relationship among~~It is assumed that~~ S_t, S_t^*, U_t, and V_t~~—, may be correlated to each other as they respond to the fluctuation of common environmental variables.~~ However, the correlation among them satisfies that $\mathrm{Cov}[S_t, U_t - V_t]$ is greater than $\mathrm{Cov}[S_t^*, U_t - V_t]$.~~,~~ which gives the mathematical definition of the field and refuge. The specific joint distribution

for these variables will be given below for simulation.

Analytic results: the fate of the A_2 allele

This section aims to predict the fate of the rare A_2 allele — whether it will be lost, fixed, or increased to intermediate frequencies and maintained polymorphic in the population — as a function of the demographic and fitness parameters in the TP model. For convenience in the derivation, only the expected changes in the numbers of A_1- and A_2-carrying individuals will be tracked while ignoring genetic drift. In addition, ~~is ignored and~~ a fixed scheme of migration, $m_{FR} = 1 - m_{RF} = r$, that sends a constant fraction r of the population to the refuge regardless of the current location is assumed. (Below the dynamics will be examined in simulation without this restriction in the migration rate.) First, soft selection, under which $N_F^{(t)} = K_F^{(t)}$ and $N_R^{(t)} = K_R^{(t)}$, is assumed. Let n_1 and n_2 be the total numbers of individuals (counted after reproduction/selection and before migration) carrying A_1 and A_2 in generation t – 1 and n'_1 and n'_2 be their expected numbers in generation t. Then, with $n_1 \gg n_2$,

$$\frac{n_2'}{n_2} \approx (1-r)\frac{K_F^{(t)}}{\tilde{N}_F^{(t)}}(1+S_t) + r\frac{K_R^{(t)}}{\tilde{N}_R^{(t)}}(1+S_t^*) = \frac{(1+S_t)K_F^{(t)} + (1+S_t^*)K_R^{(t)}}{K_F^{(t-1)} + K_R^{(t-1)}} \quad (1)$$

(Appendix A). This equation takes into account that the change in the absolute number of A_2 is determined first by demography, as the size of field changes from $\tilde{N}_F^{(t)}$ to $K_F^{(t)}$ and that of refuge from $\tilde{N}_R^{(t)}$ to $K_R^{(t)}$, and also by fitness $1 + S_t$ and $1 + S_t^*$. Whether the copy number of rare allele A_2 is expected to increase or decrease is determined by the expectation of $\log\left[\frac{n_2'}{n_2}\right]$ over demographic and fitness fluctuations. It is shown in Appendix A that the condition for the A_2 allele being positively selected (i.e. successfully invading the population initially fixed for A_1), $\mathrm{E}\left[\log\left[\frac{n_2'}{n_2}\right]\right] > 0$, is simplified to

$$\frac{\mathrm{Cov}[S-S^*, U-V]}{\mathrm{Var}[S] + \rho^2\mathrm{Var}[S^*] + 2\rho\mathrm{Cov}[S,S^*]} = \frac{\mathrm{Cov}[S-S^*, \log[K_F/K_R]]}{\mathrm{Var}[S] + \rho^2\mathrm{Var}[S^*] + 2\rho\mathrm{Cov}[S,S^*]} > \frac{1}{2\rho} \quad (2)$$

where $\rho = K_{R0}/K_{F0}$ (note that $K_F^{(t)} = K_{F0}\mathrm{e}^{U_t}$ and $K_R^{(t)} = K_{R0}\mathrm{e}^{V_t}$) represents the relative size of the refuge and super-/subscripts for time t in variables were omitted.

If it is assumed that selection on A_2 in the refuge is weaker than that in the field by a ratio δ, namely $S_t^* = \delta S_t$, the above inequality is simplified to

$$\Phi \equiv \frac{\mathrm{Cov}[S, \log[K_F/K_R]]}{\mathrm{Var}[S]} > \frac{(1+\delta\rho)^2}{2(1-\delta)\rho} \quad (3)$$

Therefore, the rare A_2 allele is positively selected if the covariance between its fitness and the log-ratio of the field to refuge size is large relative to the amplitude of the fitness fluctuation. This requires that the demographic fluctuation in the population be sufficiently large, as already shown in Kim (2023), and that, to allow the substantial fluctuation of $K_F^{(t)}/K_R^{(t)}$, the carrying capacity of the refuge should not covary strongly with that of the field. Since this requirement for the refuge is essential and distinguishes the current from the previous models of storage effect, the evolutionary force driving this positive selection may be called the noncovarying storage effect (NSE; see Discussion for examples of noncovarying and covarying storages). The conditions (2) and (3) are easier to meet as δ becomes small well below 1, namely when the fitness fluctuation of A_2 becomes either smaller in the refuge compared to the field or negatively correlated to S_t.

In a similar manner, the condition for the A_1 allele to invade a population initially fixed for A_2 under soft selection becomes

$$\frac{\mathrm{Cov}[S-S^*, U-V]}{(1+2\rho)\mathrm{Var}[S]+(2\rho+\rho^2)\mathrm{Var}[S^*]-2\rho\mathrm{Cov}[S,S^*]} < \frac{1}{2\rho} \quad (4)$$

(Appendix A) or

$$\Phi < \frac{1+\delta^2}{1-\delta} + \frac{(1+\delta\rho)^2}{2(1-\delta)\rho} \quad (5)$$

if $S_t^* = \delta S_t$.

In a simpler case with δ = 0, from inequalities (3) and (5), it is predicted that the A_1 and A_2 alleles coexist in the population if $1/(2\rho) < \Phi < 1 + 1/(2\rho)$ under soft selection. With $\Phi < 1/(2\rho)$, the A_2 allele is eliminated from the population, as the fitness fluctuation that reduces its geometric mean in the field is more important than population size fluctuation. With $\Phi > 1 + 1/(2\rho)$, the A_2 allele is predicted to reach fixation in the population.

Using the same approach, the fate of the A_2 allele under hard selection can be analyzed. Note that the carrying capacities of the field and refuge are modified to $(1 + qS_t)K_F^{(t)}$ and $(1 + qS_t^*)K_R^{(t)}$ with hard selection where q is the frequency of A_2. The frequency change of A_2 when rare is therefore effectively identical to that in soft selection. Therefore, the condition for A_2 invading the population fixed for A_1 is again given by inequalities (2) and (3). However, the condition for the fixation of A_2 is drastically different from that under soft selection. It is shown in Appendix A that

the condition for $\mathrm{E}\left[\log\left[\frac{n_1'}{n_1}\right]\right] > 0$ for rare A_1 is exactly the condition for $\mathrm{E}\left[\log\left[\frac{n_2'}{n_2}\right]\right] < 0$ for rare A_2. Therefore, with $S_t^* = \delta S_t$, the condition for the A_1 allele to invade the population fixed for A_2 is approximately $\Phi < (1+\delta\rho)^2/(2(1-\delta)\rho)$. This means that the fixation of A_2 is ensured if $\Phi > (1+\delta\rho)^2/(2(1-\delta)\rho)$; once positively selected after its appearance by mutation, the A_2 allele is expected to increase in frequency all the way to fixation. This leads to the amplification of fluctuation in the size of the field, from $K_F^{(t)}$ to $(1+S_t)K_F^{(t)}$. This result is consistent with the finding in Kim (2023) that, under weak density regulation of the population size, an allele with a wider oscillation in absolute fitness reaches fixation in the presence of refuge.

One-locus simulation

To validate the above analysis and explore further eco-evolutionary dynamics in the TP model, the one-locus stochastic simulation was performed. The demographic and fitness fluctuations were modeled by $U_t = a_U Z_1 + b_U Z_2$, $V_t = a_V Z_1 + b_V Z_3$, $S_t = a_S Z_1 + b_S Z_4$, and $S_t^* = a_{S^*} Z_1 + b_{S^*} Z_5$ where a_X (b_X) is a constant for determining the magnitude of change in variable X due to a common environmental factor (other factors) and the Z_is are standard normal variates that were drawn independently each generation. Therefore, Z_1 represents the state of the environment affecting both population sizes and fitness. To avoid negative fitness, the distributions of S_t and S_t^* were truncated at the lower bound -0.999.

Simulations were first performed for a scenario in which A_2 allele is completely protected from selection in the refuge ($S_t^* = 0$) and migration follows a simple scheme ($m_{FR} = m_{RF} = 0.5$). As suggested by the analysis above, the major parameter of eco-evolutionary dynamics in this case is $\Phi = \mathrm{Cov}[S, U-V]/\mathrm{Var}[S] = (a_S a_U - a_S a_V)/(a_S^2 + b_S^2)$. Simulation results were obtained for various values of Φ under soft (Fig. 2A, B) and hard (Fig. 2C, D) selection. Initially the numbers of A_1 and A_2 individuals changed deterministically once the demographic and fitness parameters (U, V, and S) for a given generation were sampled from their joint distribution, effectively assuming that the sizes of the field and refuge were finite and fluctuating but very large so that genetic drift can be ignored (Fig. 2A). Various values of a_S and b_S were chosen so that Φ ranged from 0.3 to 2, while ρ was set to 1. Starting from 0.5, the frequency of the A_2 allele at the 5,000th generation was recorded for 1000 replicates per parameter set. As predicted, with $\Phi \leq 0.5$, the frequency of A_2 approached 0, thus confirming negative selection, and with $\Phi \geq 1.5$ the frequency approached 1, thus confirming positive directional selection. The distribution of the allele frequency was

approximately uniform between 0 and 1 when Φ = 1. Next, genetic drift and bidirectional mutation between two alleles were added to the above simulations (Fig. 2B). This weakened the strengths of all modes of selection. With Φ = 1, more simulation runs ended with allele frequencies close to 0 and 1. However, the proportion of runs ending with intermediate allele frequencies was still larger than that observed when the alleles were set as neutral (dashed curve in Fig. 2B). Therefore, an evolutionary force to maintain polymorphism beyond the level under the neutrality, namely balancing selection, was confirmed.

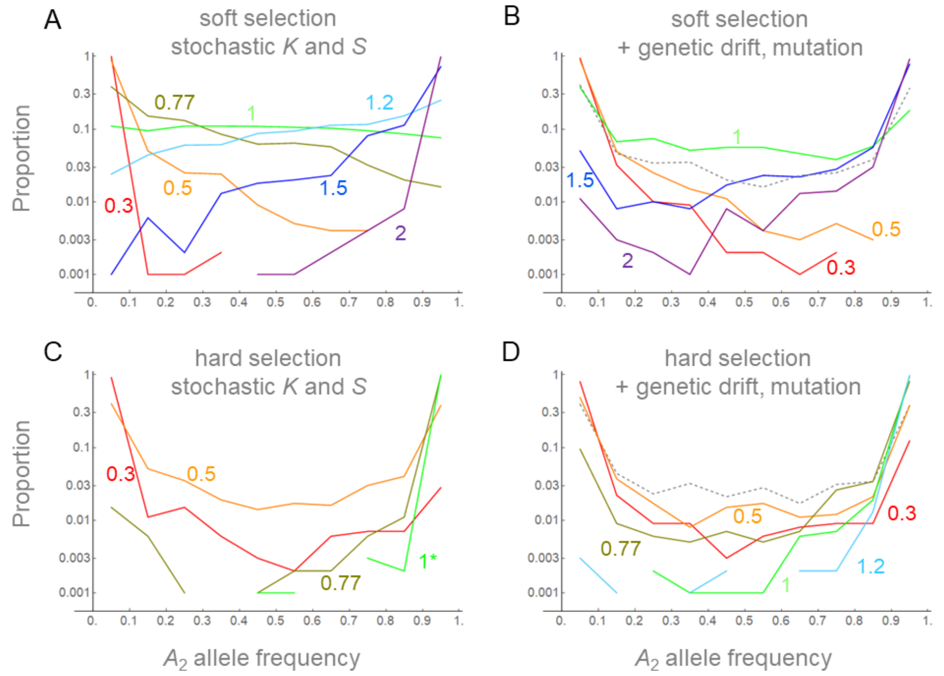

Figure 2. Distribution of A_2 allele frequencies (p) at the 5,000th generation in the stochastic simulations of soft (A, B) and hard (C, D) selection in the TP model. The initial frequency of A_2 is 0.5 in both field and refuge. Reproduction occurred while $K_F^{(t)}$, $K_F^{(t)}$ and S_t varied stochastically without (A, C) or with (B, D) genetic drift and bidirectional mutation ($\mu = 5\times10^{-5}$). Proportions of simulation replicates (out of 1000) with the frequencies in the interval $0 \le p < 0.1$, $0.1 \le p < 0.2$, ..., $0.8 \le p < 0.9$, and $0.9 \le p \le 1$ were plotted and connected by lines. Fluctuations of population sizes are given by $K_{R0} = K_{F0} = 1000$ ($\rho = 1$), $a_U = 0.3$, $b_U = 0.1$, $a_V = 0.05$, $b_V = 0.1$. Parameters of fluctuation were chosen to yield Φ = 0.3, 0.5, 0.77, 1, 1.2, 1.5, and 2: (a_S, b_S) = (0.07, 0.23), (0.1, 0.2), (0.14, 0.16), (0.2, 0.1), (0.2, 0.04), (0.15, 0.05), and (0.1, 0.05). In the case of hard selection with Φ = 1 and no genetic drift (panel C), simulation ran only up to 2,500 generations: longer simulations or larger Φ resulted in all replicates with final frequencies > 0.9. The results connected by dashed lines in B and D represent the cases of neutral evolution ($a_S = b_S = 0$).

Similarly, the fate of the A_2 allele under hard selection was examined. Stochastic simulation confirmed the approximation above predicting that A_2 is positively selected towards fixation if $\Phi > \frac{1}{2\rho} = 0.5$, although genetic drift and mutation weakened the trend (Fig 2C,D). Positive selection on both rare and common A_2 under the same condition means that this allele may not remain polymorphic for long in the population. Indeed, across all values of Φ there were fewer simulation runs ending with intermediate frequencies of A_2 compared to the simulation of neutral alleles subject to genetic drift and bidirectional mutations only (dashed curve in Fig. 2D). Therefore, balancing selection that was observed with soft selection did not arise with hard selection.

Next, the cases in which A_2 allele is not completely neutral in the refuge was examined. A simple model setting $S_t^* = \delta S_t$ was used. This time, the A_2 allele is initially absent in the population but introduced with recurrent mutation. For a given parameter set simulation was run over at least 10^5 generations and the mean $\overline{H}$ of expected heterozygosity ($2q(1-q)$, where q is the relative frequency of A_2) and the proportion of time when $0 \le q < 0.1$, P_{01}, and $0.9 < q \le 1$, P_{90}, during simulation were recorded. Simulation with $S_t = S_t^* = 0$ but under the same demographic structure yielded $\overline{H}$ = 0.063 (average over 6×10^5 generations). Then, as balancing selection is defined to be a force yielding genetic variation above the neutral level, a result was classified as balanced polymorphism ("balanced" in Fig. 3) if $\overline{H}$ > 0.075. If $\overline{H}$ < 0.05 and $P_{01} > P_{90}$, it suggests that negative selection against A_2 was the dominant evolutionary force. Such results were therefore classified as the loss of A_2 ("lost" in Fig. 3), since recurrent losses of A_2 should be the major events in the simulation (confirmed by visual inspection of allele frequency trajectories; not shown). Conversely, results yielding $\overline{H}$ < 0.05 and P_{90} > 0.7, which indicates that positive directional selection on A_2 was the dominant force, were classified as the fixation of A_2 ("fixed" in Fig. 3). All others were classified as not distinguishable from the neutral evolution. Simulations largely confirmed the analytic approximations for the conditions for the loss, fixation, or balanced polymorphism of A_2 in a population initially fixed for A_1 (e.g., $\frac{(1+\delta\rho)^2}{2(1-\delta)\rho} < \Phi < \frac{1+\delta^2}{1-\delta} + \frac{(1+\delta\rho)^2}{2(1-\delta)\rho}$ for the balanced polymorphism under soft selection and $\Phi > \frac{(1+\delta\rho)^2}{2(1-\delta)\rho}$ for the fixation under hard selection; Fig. 3). It is shown that increasing δ above 0 makes it harder for balancing and positive directional selection on A_2 to occur; for a large value of δ, the fluctuation (variance) of S_t needs to be much smaller than its covariance with the fluctuation of $\log[K_F^{(t)}/K_R^{(t)}]$ (eq. 3). Small Var[S_t]

then means that the relative strength of genetic drift affecting the evolutionary dynamics of A_2 is large, explaining the results not distinguishable from the neutrality with large δ and Φ in Figure 3. Other results not distinguishable from neutral evolution were obtained when the predicted effects are near the boundary between balancing and directional selection. Another important result is that negative δ, thus selection in the refuge being in the opposite direction, widens the parameter range for balancing selection.

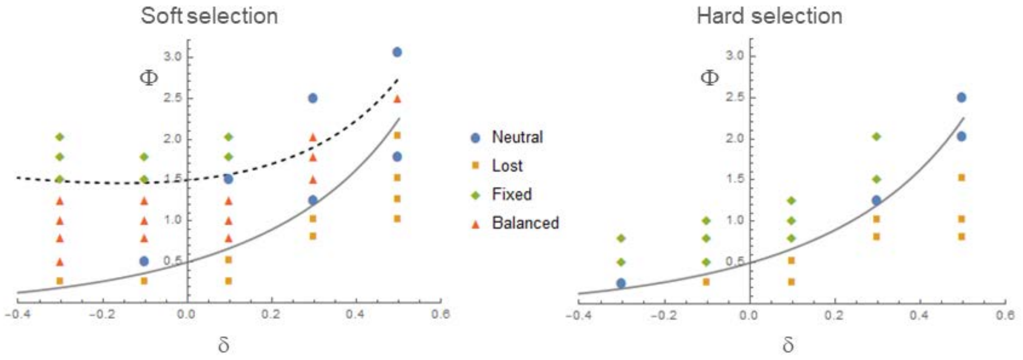

Figure 3. Simulation results for non-zero values of δ. For a given parameter set simulation started with the A_1 allele fixed in the population and ran for 10^5 generations with recurrent bidirectional mutations and soft (left) or hard (right) selection. Four different outcomes regarding the overall behavior of the A_2 allele (balanced, lost, fixed, neutral) were marked by different symbols and plotted as functions of δ and Φ. The demographic parameters are identical to those in Figure 1. Five different values of δ (-0.3, -0.1, 0.1, 0.3, and 0.3) and ten different values of Φ were chosen: 0.246, 0.5, 0.8, 1, 1.25, 1.5, 1.786, 2.027, 2.5 and 2.94: (a_S, b_S) = (0.05, 0.22), (0.1, 0.2), (0.2, 0.15), (0.2, 0.1), (0.2, 0), (0.15, 0.05), (0.14, 0), (0.12, 0.02), (0.1, 0) and (0.085, 0). Gray and dashed curves plot $\Phi = \frac{(1+\delta\rho)^2}{2(1-\delta)\rho}$ and $\Phi = \frac{1+\delta^2}{1-\delta} + \frac{(1+\delta\rho)^2}{2(1-\delta)\rho}$ with $\rho = 1$.

Finally, the robustness of balancing and positive directional selection above to deviations from the simple migration scheme, $m_{FR} = 1 - m_{RF} = r$, was examined. In actual populations, migration rates may vary over time and may not be symmetrical between the field and refuge, at least in a short time scale. New simulations therefore sampled migration rates m_{FR} and m_{RF} from uniform distributions in ranges $[m_1, m_2]$ and $[m_3, m_4]$, respectively, each generation (Table S1). With ρ = 1 (K_{F0} = K_{R0}), limiting both m_{FR} and m_{RF} close to 0.5 yielded the highest level of balanced polymorphism under soft selection. However, allowing random deviation from 0.5 (e.g. $[m_1, m_2]$ =

[m_3, m_4] = [0.3, 0.7]) only slightly lowered the level of polymorphism. Smaller migration rates generally disrupted balancing selection. Interestingly, the effect of m_{FR} and m_{RF} was not symmetric; balancing selection remained effective with m_{RF} close to 0.5 and m_{FR} less than 0.2, however not with the opposite ratio of rates (compare cases with [[m_1, m_2], [m_3, m_4]] = [[0.1, 0.2], [0.4, 0.5]] and [[0.4, 0.5], [0.1, 0.2]] in Table S1). Similar effects of m_{FR} and m_{RF} on the directional increase of A_2 allele frequency under hard selection were observed.

In summary, one-locus simulations for the TP model not only confirmed the analytic approximations for the conditions under which the ~~noncovarying storage effect~~NSE leads to either balanced polymorphism or the fixation of the fluctuation-amplifying allele but also showed that the effect is robust to perturbations in the model such as genetic drift and randomness/asymmetry in the migration rate.

Multi-locus simulation

To investigate how the eco-evolutionary dynamics generated by the ~~noncovarying storage effect~~NSE in the TP model play out when there are many such loci in the genome, new sets of simulations were performed in which a haploid individual is modeled to have L linked loci, each carrying either the A_1 or A_2 allele described above. To avoid exploring too many parameter dimensions while focusing on the effect of increasing the number of loci, $S_t^* = 0$ and a fixed migration scheme ($m_{FR} = m_{RF} = 0.5$) are assumed. The simulation tracks the numbers of individuals of different haplotypes (see Appendix B). Epistasis is not considered; the relative fitness of haplotype i is given by $W_i = \prod_{l=1}^{L}(1 + S_{t,l})$, where $S_{t,l}$ is 0 if the lth locus of the haplotype carries A_1 and $a_S Z_1 + b_S Z_4$ if A_2. Z_1 is drawn once for all loci while Z_4 is drawn separately for different loci. Individuals randomly pair and undergo recombination by crossing-over such that the probability of recombination between adjacent loci is c per generation. The simulation starts with a population fixed for A_1 at all loci. Then A_2 arises by mutations with probability μ/locus/generation.

Two major outcomes of the NSE~~noncovarying storage effect~~ – balancing selection and positive directional selection – were addressed. First, considering that (1) the negative frequency-dependent selection on the A_2 allele was caused primarily by its fitness correlated to the fluctuation in the carrying capacity (population size) of the field and that (2) this demographic fluctuation should be "felt" at all loci in the genome, one may expect that such balancing selection

can occur simultaneously at many loci harboring such variants. To verify if this is true, simulations were run under soft selection and demographic/selective parameters that promoted polymorphism in the single-locus model (Φ = 1 for ρ = 1) with L = 1, 4, or 10. Exemplary trajectories of allele frequencies are shown in Fig. S1. Each run lasted at least 10^5 generations over which $\overline{H}$, the expected heterozygosity ($2p(1-p)$) averaged over time and loci, and the proportion of time the A_2 alleles spent in frequency between 0.1 and 0.5, P_{15}, and between 0.5 and 0.9, P_{59}, were recorded (Table 1). In all cases with fluctuating

Table 1. The long-term level of polymorphism in the multi-locus simulation

demography	selection mode	L	c	$\overline{H}$	P_{15}	P_{59}
a_U = 0.2, a_V = b_U = b_V = 0.1	neutral (a_S = b_S = 0)	1	-	0.0639	0.069	0.074
	soft selection, Φ = 1 (a_S = 0.1, b_S = 0)	1	-	0.132	0.15	0.16
		4	10^{-4}	0.0960	0.099	0.12
		4	10^{-3}	0.103	0.12	0.12
		4	0.01	0.0977	0.11	0.12
		4	0.1	0.136	0.11	0.14
		10	10^{-4}	0.080	0.087	0.095
		10	10^{-3}	0.102	0.12	0.12
		10	0.01	0.109	0.15	0.10
		10	0.1	0.107	0.13	0.12
a_U = 0.3, a_V = 0.05, b_U = b_V = 0.1	neutral (a_S = b_S = 0)	1	-	0.0633	0.081	0.074
	soft selection, Φ = 1 (a_S = 0.2, b_S = 0.1)	1	-	0.177	0.24	0.18
		4	10^{-4}	0.162	0.19	0.19
		4	10^{-3}	0.130	0.21	0.11
		4	0.01	0.168	0.22	0.18
		4	0.1	0.183	0.25	0.19
		10	10^{-4}	0.120	0.14	0.14
		10	10^{-3}	0.141	0.17	0.17
		10	0.01	0.0934	0.076	0.14
		10	0.1	0.105	0.093	0.15
a_U = 0.05, a_V = b_U = b_V = 0	neutral (a_S = b_S = 0)	1	-	0.0660	0.085	0.063
	soft selection, Φ = 1 (a_S = 0.05, b_S = 0)	1	-	0.0816	0.11	0.081
		10	10^{-4}	0.0728	0.085	0.079
		10	10^{-3}	0.0982	0.12	0.11
		10	0.01	0.0953	0.12	0.096
		10	0.1	0.0952	0.13	0.092

- Simulations were run for 5×10^5, 2×10^5, and 10^5 generations with L = 1, 4, and 10, respectively.
- Other parameters: K_{R0} = K_{F0} = 1,000 (ρ=1), μ = 2×10^{-5}.

fitness ($a_S > 0$), $\overline{H}$ was above the level observed in equivalent simulations with neutral alleles ($S_t = 0$), thus confirming multi-locus balancing selection. For cases of stronger selection (larger Var[S_t]), the level of polymorphism per locus declined as L increased from 1 to 10. The combined effects of the number of loci and the rate of recombination were not easy to interpret as they depended on the parameters of selection (for sets of fitness/demographic parameters that yield the same Φ = 1, $\overline{H}$ responded differently with varying L and c). One possible explanation for generally lower variation with 10 loci instead of 1 or 4 is that the simultaneous segregation of alleles at many loci increased the variance of the offspring numbers, thus decreasing the effective population size and weakening the negative frequency-dependent selection that is needed for protected polymorphism. Further exploration of multi-locus polymorphism at a larger scale (in both number of loci and population size) may not be feasible with the current simulation approach based on haplotype frequencies.

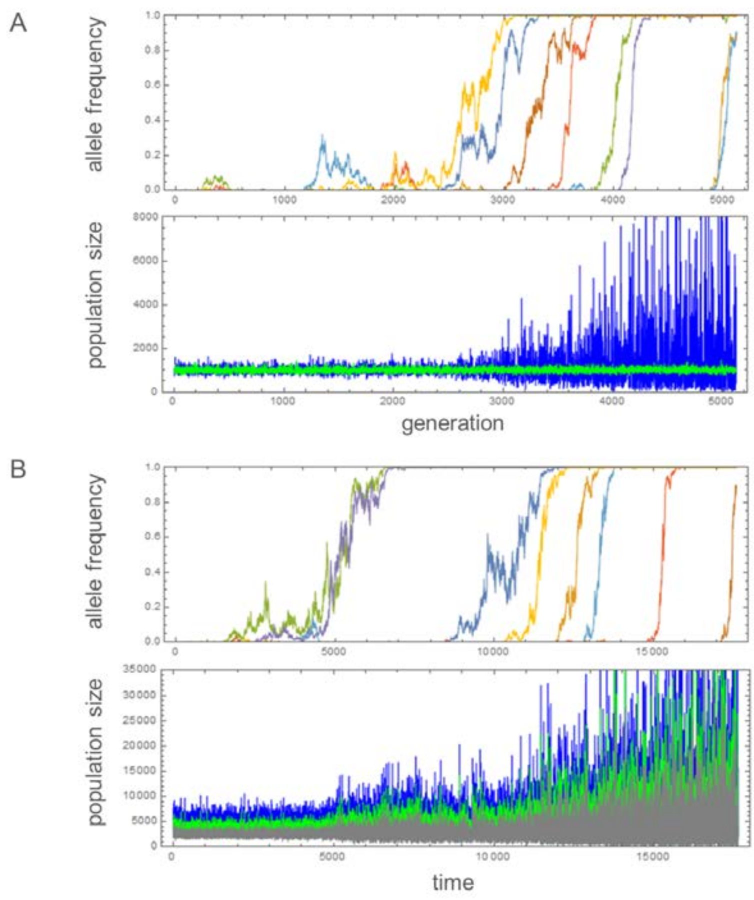

Figure 4. Changes of mutant (A_2) allele frequencies and subpopulation sizes (A. TP model, blue: $N_F^{(t)}$, green: $N_R^{(t)}$; B. LSA model, blue: larval, green: subadult, gray: adult population) in the multi-locus simulations of hard selection. Allele frequency trajectories of 8 loci were plotted in different colors. Parameters: A (TP model): L = 8, c = 0.02, K_{R0} = K_{F0} = 1000, μ = 2×10^{-5}, and Φ = 1 (a_S = 0.15, b_S = 0, a_U = 0.15, a_V = 0, b_U =0, b_V = 0.1). B (LSA model): L = 8, c = 0.02, K_{L0} = 5000, μ = 2×10^{-5}, and Φ' = 1 (a_S = 0.1, b_S = 0.1, a_L = 0.2, b_L= 0.1).

Second, simulation was performed with parameter values under which directional selection for the A_2 allele is expected in the single-locus model ($\Phi > 1 + 1/(2\rho)$ for soft selection and $\Phi > 1/(2\rho)$ for hard selection). As expected, substitutions occurred sequentially until A_2 reached fixation at all L loci (Fig. 4A and Fig. S2 for example). Times (in generations) when the frequency of A_2 first exceeded 0.9 at each locus were recorded and sorted into T_1 to T_L. There is a large difference in substitution dynamics between soft and hard selection. With soft selection, the mean waiting time, $\bar{T} = (T_1 + \cdots + T_L)/L$, increased moderately as L increased from 1 to 8 (Fig. 5A). This may be explained by a reduction in the efficacy of positive selection on A_2 at a given locus when selection at other loci results in genome-wide reduction in the effective population size.

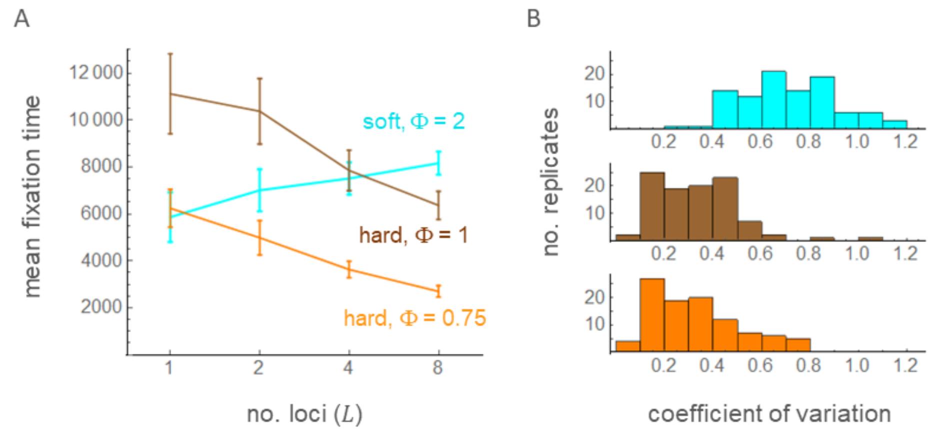

Figure 5. Mean fixation time ($\bar{T}$) with varying number of loci (A; L = 1, 2, 4, and 8) and the coefficient of variation in fixation times with L = 8 in the multi-locus simulation of the TP model. Error bars show two times the standard errors. A total of 100 replicates of simulations were run under soft selection with Φ = 2 (cyan; a_S = 0.1, b_S = 0, a_U = 0.25, b_U = a_V = b_V = 0.05), hard selection with Φ = 1 (brown; a_S = 0.1, b_S = 0, a_U = 0.1, a_V = 0, b_U = b_V = 0.1), or hard selection with Φ = 0.75 (orange; a_S = 0.2, b_S = 0, a_U = 0.25, a_V = 0.1, b_U = b_V = 0.05). Other parameters are identical to those in Figure 4A.

With hard selection, however, $\bar{T}$ decreased as L increased. Furthermore, the coefficient of variation, the standard deviation of T_i's divided by $\bar{T}$, was significantly smaller with hard selection than with soft selection (Fig. 5B). As expected for hard selection, the amplitude of the population size fluctuation progressively increased as the number of loci fixed for A_2 increased (Fig. 4A). These results clearly demonstrate that positive feedback between demographic and selective fluctuations under hard selection led to a great amplification of population size fluctuation; an initial fluctuation in $K_F^{(t)}$ positively selected the mutant allele A_2 at one locus whose fitness

fluctuates in positive correlation with $K_F^{(t)}$, leading to the fixation of A_2 and therefore a larger fluctuation of the field (given by $(1+S_t)K_F^{(t)}$), which makes it easier for the A_2 allele at another locus to be positively selected. This process could continue to increase the fluctuation of the field until its carrying capacity is given by $(1+S_t)^L K_F^{(t)}$, where L is the number of loci available in the genome for mutation into A_2. There was also a moderate effect of the recombination rate in accelerating the multi-locus fixation of A_2 with hard selection (Table S2). Fixation times generally increased with decreasing recombination rates for both soft and hard selection, possibly because tighter linkage increased the occurrence of clonal interference.

Larval-subadult-adult (LSA) model

Although the refuge in the TP model is expected to play the role of various demographic/genetic elements that were previously shown to produce the storage effect, such as seed banks, diapausing stages, or a particular life-stage in a population with overlapping generations, the noncovarying storage effect found in the TP model may not be replicated in other population structures as the nature of refuge (i.e. whether it undergoes its own reproduction and whether the lineage of an allele can persist in a refuge for many generations) and the pattern of 'migration' in and out of the refuge can be qualitatively different. I therefore investigate an explicit discrete-time model of an age-structured population with overlapping generations. Three stages – 'larval', 'subadult', and 'adult' – in the life cycle of a haploid species are assumed. Subadult means the early stage of reproducing adults. Time is counted in intervals of the length required for one life stage to advance to the next one. At the end of each interval, the final size of larval population is regulated to its carrying capacity ($K_L^{(t)}$ at time t). Probabilities for an individual to advance from larval to subadult and from subadult to adult stages are given by e_L and e_S, respectively. Therefore, the sizes of the subadult and adult populations are expected to be $e_L K_L^{(t-1)}$ and $e_L e_S K_L^{(t-2)}$ at time t. $K_L^{(t)}$ is assumed to vary randomly over time with no autocorrelation. Specifically it is modeled to be $K_L^{(t)} = K_{L0}\exp(a_L Z_1 + b_L Z_2)$, where Z_1 and Z_2 are standard normal variables drawn each time.

The population is made of haploids carrying either the A_1 or A_2 allele at each of L linked loci. The (marginal) fitness of A_2 relative to A_1 at locus j is given by $1+S_{j,t}$ (j = 1, ..., L), where $S_{j,t} = a_S Z_1 + b_S Z_{3,j}$. $Z_{3,j}$ is drawn independently for different loci at each interval t. Then, the fitness

of haplotype i, $W_i^{(t)}$, is given by the multiplication of allelic fitness across all loci. It is assumed that this haplotype fitness determine the viability in the larval stage only; the carriers of two alleles are not different either in the probability of advancing to the next stage or in fertility in the subadult and adult stages. If there are only larval and subadult stages, they constitute a population that reproduces in effectively discrete generations. The addition of the (old) adult stage provides a storage for alleles regardless of their reproductive success in the previous (subadult) stage. Therefore, the adult stage plays the role of the refuge and is an example of a noncovarying storage because its size is independent of the concurrent subadult stage.

Multi-locus simulations, allowing not only genetic drift in the reproduction step but also stochastic advances from one to the next life stage (see Appendix B for method), were performed with limited sets of parameters, each starting with a population fixed for haplotype 0, which carries A_1 at all loci. The evolutionary dynamics of the A_2 alleles in each simulation run over 50,000 time units was summarized by the mean heterozygosity and the proportion of time A_2 remained close to relative frequency 0 or 1 (Table S3). Major results produced in the TP model – multi-locus balancing selection under soft selection or positive directional selection on A_2 under hard selection – were readily obtained with this model. In agreement with Kim (2023) and the TP model, the relative strength of demographic vs. fitness fluctuation determined the outcome; when we define $\Phi' = \mathrm{Cov}\left[\log[K_L^{(t)}/K_{L0}], S_{j,t}\right]/\mathrm{Var}\left[S_{j,t}\right] = a_L a_S/(a_S^2 + b_S^2)$, which is analogous to Φ in the TP model, positive selection on rare A_2 occurred when Φ' was above 0.7. With soft selection and above this threshold of Φ', the frequency of A_2 underwent sustained oscillations that are indicative of balancing selection (Fig. S3). Then, as Φ' continued to increase, balancing selection transitioned into positive directional selection. With hard selection, positive directional selection occurred once Φ' increased above the threshold. Exemplary replicates show that, as the A_2 alleles reach fixation across loci, fluctuations in the sizes of all three life stages become amplified (Fig. 4B, Fig. S3). Negative (positive) correlation between mean fixation times and the number of loci under hard (soft) selection and the clustering of fixation times with hard selection were also observed in other sets of simulations (Fig. S4), which basically replicated the results from the TP model (Fig. 5). This confirmed that positive feedback between demographic and fitness fluctuations under hard selection occurs in both models. In conclusion, all major aspects of the ~~noncovarying storage effect~~NSE found in the TP model were shown to arise in the LSA model as well.

DISCUSSION

A key assumption in this study is that a tradeoff (antagonistic environmental pleiotropy; Zhang 2023) commonly occurs for the fitness effects of a mutant allele between different time points in the environmental fluctuation. Namely, a mutant phenotype that increases the reproductive output relative to others when the environment is generally favorable is likely to suffer a decline in reproductive output~~decrease it~~ relative to others during an unfavorable period. Such a mutant allele increases the variance of the offspring number over time while the mean may remain unchanged. For example, as postulated by Slatkin (1974), a mutation that makes a female bird lay 10 eggs in one batch instead of spreading them over time in a randomly and rapidly fluctuating environment will increase the variance of her fitness while the mean remains the same. Setting the fitness of a mutant to 1 + S_t with E[S_t] = 0, thus an AMF mutant, models such a tradeoff. Classical theory predicts that such a mutant~~ (termed an arithmetic-mean-preserving or AMF allele in Kim (2023))~~ is eliminated from a population as its geometric mean fitness is less than 1.

Kim (2023) and this study however found that an AMF mutant at a low frequency can be positively selected (with both soft and hard selection) if a correlation between demographic and fitness fluctuations exists in the presence of a refuge whose carrying capacity oscillates in a narrower range and/or out of phase relative to the field. A heuristic argument for this NSE on the fitness-amplifying mutant allele may be given. In the TP model, the mean number of offspring per parent, *i.e.* the absolute fitness, of the rare mutant in the field is given by $W_{2F} = \frac{K_F^{(t)}}{\tilde{N}_F^{(t)}}(1 + S_t)$ (eq. 1), where $K_F^{(t)}/\tilde{N}_F^{(t)}$ is the absolute fitness given under demographic fluctuation alone. With a positive correlation between $K_F^{(t)}$ and S_t, the fluctuation of W_{2F} becomes greater than that of $1 + S_t$ and, more importantly, its arithmetic mean can now exceed 1. Still, the geometric mean of W_{2F} is not greater than 1 due to its fluctuation over time. However, the presence of a refuge can reduce the variance of the mutant's fitness averaged across the whole population such that the geometric mean of this average fitness becomes greater than 1. Here, to generate this dampening effect, the refuge should not undergo the same fluctuation in size with the field; the absolute fitness in the refuge $W_{2R} = \frac{K_R^{(t)}}{\tilde{N}_R^{(t)}}(1 + S_t^*)$ in eq. (1) should fluctuate differently than W_{2F}. With parameter values used in of the TP model, $K_R^{(t)}$ was given to covary incompletely with $K_F^{(t)}$. In the LSA model, the fluctuation of the refuge (the adult stage) is uncorrelated to the fluctuation of the field (the subadult stage). In Kim (2023), the seed bank fluctuates in size similar to the subadult and adult stages in the LSA model. In summary, such refuges, or noncovarying storages,

turn the heightened arithmetic mean of the mutant allele raised by the demographic fluctuation of the field into an elevation of the geometric mean in the total population.

Not all refuges in the previously proposed mechanisms of the storage effect are noncovarying. For example, it was suggested that if a gene is expressed in one sex in a dioecious species the other sex could be a refuge (Reinhold 2000; Kim 2023). However, as equal numbers of male and female gametes are used in reproduction, the subpopulation sizes of reproductive males and females are effectively equal while the sex ratio of the adults may fluctuate. Therefore, as the size of the field relative to the refuge does not fluctuate at all, the ~~noncovarying storage effect~~NSE should not arise for the genes of the sex-limited expressions.

One may also assume GMF mutations; ~~model~~ the fluctuating fitness of a mutant can be given by $\exp[S_t]$, for example, making the mutant ~~(termed a geometric-mean-preserving or GMF allele)~~ quasi-neutral as its geometric mean fitness is identical to that of the wild-type allele even without demographic fluctuation. Analyzing the model of cyclic environmental oscillation, Kim (2023) showed that the parameter range of positive selection for a rare GMF allele is wider than that for an AMF allele. Similarly, GMF alleles should be subject to the ~~noncovarying storage effect~~NSE under broader conditions. However, a GMF allele may be considered simply a beneficial allele as the arithmetic mean of offspring number is greater than the wild-type allele. Such a mutation is probably rare and therefore was not considered in this study. One should however note that GMF alleles might be still important for genetic variation in nature since they can be maintained in balanced polymorphism in the conventional models of the storage effect, for example without demographic fluctuations and/or in the case of sex-limited gene expressions.

Analytic approximation for the one-locus TP model predicts that, under soft selection or strict density regulation that keeps the population size to the carrying capacity of the field at a given time regardless of its genetic composition, balancing selection arises if the demographic fluctuation (a major contributor to $\mathrm{Cov}[S, \log[K_F/K_R]]$) is not so weak or strong compared to fitness fluctuation ($\mathrm{Var}[S]$ in case of $S^* = 0$). ~~Here, balancing selection is defined as a force that generates polymorphism at a level greater than expected for neutral alleles.~~ Multi-locus simulation showed that balanced polymorphism can be simultaneously maintained at many loci, as expected since the demographic fluctuation (together with the presence of refuge) that turns the geometric mean fitness of an AMF allele from < 1 to > 1 should apply equally to all loci in the genome. Previously, Park and Kim (2019) showed that, in the presence of a refuge but without fluctuation in population size, polymorphism can arise at multiple loci that contribute to the

expression of a phenotype under fluctuating selection. However, the number of loci where balanced polymorphism emerged increased very slowly as the total number of loci in the model increased. In contrast, in the current simulation, balanced polymorphism was observed at all loci available for mutations to arise. However, the model containing only up to 10 loci was examined, due to the limitation of the simulation method used, and the 10-loci polymorphism at each locus was generally lower than the level observed in the one-locus simulation. This is probably because the segregation of alleles at multiple loci elevates variance in the offspring number well above that of the Poisson distribution, thus increasing genetic drift that weakens the positive selection on rare alleles. The possibility that this mechanism can maintain the genome-wide polymorphism over 1,000 loci, currently observed in several natural populations (Bergland, et al. 2014; Johnson, et al. 2023), should be addressed by a simulation on a large scale in terms of both the number of loci and the census population size.

If the mutant (A_2) alleles that were positively selected from low to high frequencies are not maintained as a stable, oscillatory polymorphism as described above, they are fixed in the population. Such events, the fixations of mutations that amplify fitness fluctuations are another ~~This study focused more on the other evolutionary~~ outcome of the ~~noncovarying storage effect~~NSE with eco-evolutionary significance~~, the fixation of mutations that amplify fitness fluctuations~~. In the TP model, the condition for the fixation is less restrictive compared to that for balanced polymorphism, because it requires relatively weaker selection (smaller variance in relative fitness) for a given demographic fluctuation to satisfy, assuming $S^* = 0$, $\Phi > 1 + 1/(2\rho)$ with soft selection and $\Phi > 1/(2\rho)$ with hard selection. Similar ranges of conditions (for Φ') seem to apply for the LSA model (Table S3). Because fixation rather than balanced polymorphism occurs in a wider parameter space, one may argue that the overall effect of NSE (i.e. the modification of the storage effect due to population size fluctuation) is to make balancing selection less likely to occur. However, given the possible patterns of mutant alleles' fitness fluctuations in nature beyond those considered so far, the NSE may be a force of increasing, rather than decreasing, the number of loci in which oscillatory polymorphism occurs. First, the NSE causes all GMF mutants to be either lost or fixed in the population, thus no balanced polymorphism (Kim 2023), but allows AMF mutants to be maintained as balanced polymorphism. As one may expect more AMF mutations than GMF mutations to occur in the genome, since more beneficial mutations (in net effect) are considered less likely in general, the number of loci under balancing selection may increase due to the NSE. This also suggests that for a given demographic fluctuation, mutations of certain fluctuation in fitness are "picked up" to be maintained as

polymorphism. In the heuristic argument above, the absolute fitness of the A_2 allele in the field is given by $W_{2F} = D_F w_2$, where D_F is its absolute fitness given by change in the population size of the field alone and w_2 is the relative fitness of A_2 in the field (e.g., in the TP model, $D_F = \frac{K_F^{(t)}}{\tilde{N}_F^{(t)}}$ and $w_2 = 1 + S_t$). It is a limited range of fluctuation in W_{2F} that produce polymorphism, as suggested in the above model with AMF mutations where a larger fluctuation in population size (thus in D_F) allows polymorphism only when the mutant alleles have a larger fluctuation in fitness relative to the wild-type (so that $\mathrm{Var}[S]$ matches $\mathrm{Cov}[S, \log[K_F/K_R]]$), which makes the arithmetic mean of W_{2F} decline. It might be possible that for a strong demographic fluctuation under which an AMF mutant is driven to fixation, a mutant with a new pattern of fitness fluctuation not considered in this study so far, possibly with even lower arithmetic mean than AMF mutants (thus < 1), might be picked up by the NSE into balanced polymorphism.

Demographic fluctuation that generates the ~~noncovarying storage effect~~NSE should apply equally to all loci in the genome. Therefore, fixations occur at all available loci on which ~~proper~~ fitness-amplifying mutations (*i.e.* the A_2 allele) arise, as observed in the multi-locus simulation. With soft selection, the pattern of demographic fluctuation that generates the ~~noncovarying storage effect~~NSE does not change over time regardless of any evolutionary change at any locus. However, with hard selection, fluctuation in the relative size of the field generating the ~~nocovarying storage effect~~NSE at a given locus intensifies as more fluctuation-amplifying mutations reach fixation at other loci. Since the carrying capacity of the field in the TP model is effectively $(1 + S_t)^l K_F^{(t)}$, where l is the number of loci where A_2 reached fixation, and wider fluctuation generates stronger positive selection on the rare A_2 (larger $\mathrm{E}[\Delta_2]$ in Appendix A), fixation at one locus facilitates the subsequent fixations at other loci. This positive feedback between demographic fluctuation and positive selection drastically increases the amplitude of fluctuation in the total population size (Fig. 4). How much the amplitude will increase should depend on the number of loci at which mutations subject to hard selection arise. There should be multiple independent ways in which changes in mutants' relative fitness can be translated into changes in the mean absolute fitness of the entire population. For various life history traits, one may imagine phenotypes (smaller eggs, smaller adult body, earlier onset of first reproduction, etc.) that increase the absolute number of surviving offspring during a favorable, resource-rich period but decrease the number in a bad period while not affecting the amount of resources that the carriers of the alternative, ancestral alleles acquire. Then, many loci may be available for hard selection, the combined effect of which may lead to a very large fluctuation in population size.

The size of a population and its fluctuation should be governed not only by extrinsic factors (the environmental variables such as temperature and parasite prevalence) but also by intrinsic factors (species-specific responses to those extrinsic factors). The intrinsic factors such as various life-history traits are the product of the species' evolutionary history. For example, phenotypic plasticity responding to environmental cues can reduce the amplitude of the population size fluctuation, thus acting as a mechanism of population regulation (Caswell 1983; Reed *et al.* 2010), which according to the classical theory is adaptive as it enhances long-term reproductive success by increasing the geometric mean fitness (Gillespie 1977; Tuljapurkar 1982; Pfister 1998). If life history traits evolved in the direction toward suppressing random or cyclic fluctuations in the population size (Pfister 1998), fluctuations observed in nature should be interpreted as the effect of variable environments that the evolved mechanisms of population regulation were unable to contain. However, this study suggests that evolution can proceed in the opposite direction. A large random fluctuation in population size can be the product of adaptive evolution driven by positive feedback between demographic and selective fluctuations. A recent study also suggested that a species may evolve to undergo a larger fluctuation in its population size. Liu *et al.* (2019) found that a reproductive strategy increasing the temporal variance of offspring number can be advantageous in populations reproducing in overlapping generations under various patterns of environmental fluctuations. When the degrees of population size fluctuation are different across species, the eco-evolutionary dynamics proposed in this study might be particularly important for those exhibiting large fluctuations, for example mast seeding or insect outbreaks (Dwyer *et al.* 2004; White 2008; Bogdziewicz *et al.* 2020). Interestingly, annual desert plants experiencing wider fitness fluctuations after germination have greater proportions of their population remaining in the seed bank (Gremer & Venable 2014). This observation is in agreement with the analytic results in this study because a larger seed bank corresponds to a larger value of ρ, which makes it easier for the fixation of the fluctuation-amplifying alleles (i.e., lowering the threshold of Φ for positive selection) by the NSE.

DATA AVAILABILITY

Mathematica notebook files for conducting all simulations reported are available from https://github.com/YuseobKimLab/RandomFluctuationEcoEvo

ACKNOWLEDGMENT

~~This study was supported by the National Research Foundation (NRF) grant 2020R1A2C1009261 funded by the Korean government.~~I thank Kristan Schneider, Claus Vogl, Jason Wolf, Sally Otto, and anonymous reviewers who read the previous and earlier versions of the manuscript and provided comments that greatly improved the scope and depth of this study.

CONFLICT OF INTEREST

The author declares no conflict of interest.

REFERENCES

Barabás G, D'Andrea R, Stump SM. (2018). Chesson's coexistence theory. *Ecological monographs* 88:277-303.

Bell, G. (2010). Fluctuating selection: the perpetual renewal of adaptation in variable environments. *Philos Trans R Soc Lond B Biol Sci*, 365, 87-97.

Bergland AO, Behrman EL, O'Brien KR, Schmidt PS, Petrov DA. (2014). Genomic evidence of rapid and stable adaptive oscillations over seasonal time scales in Drosophila. *PLoS Genet,* 10, e1004775.

Bertram, J. & Masel, J. (2019). Different mechanisms drive the maintenance of polymorphism at loci subject to strong versus weak fluctuating selection. *Evolution*, 73, 883-896.

Bogdziewicz, M., Ascoli, D., Hacket-Pain, A., Koenig, W.D., Pearse, I., Pesendorfer, M. *et al.* (2020). From theory to experiments for testing the proximate mechanisms of mast seeding: an agenda for an experimental ecology. *Ecology Letters*, 23, 210-220.

Caswell, H. (1983). Phenotypic plasticity in life-history traits: demographic effects and evolutionary consequences. *American Zoologist*, 23, 35-46.

Chesson, P.L. (1983). Coexistence of competitors in a stochastic environment: the storage effect. *Lecture Notes in Biomathematics* 54:188-198.

Chesson, P.L. (1994). Multispecies competition in variable environments. *Theoretical Population Biology*, 45, 227-276.

Chesson, P.L. & Warner, R.R. (1981). Environmental variability promotes coexistence in lottery

competitive systems. *The American Naturalist*, 117, 923-943.

Dwyer, G., Dushoff, J. & Yee, S.H. (2004). The combined effects of pathogens and predators on insect outbreaks. *Nature*, 430, 341-345.

Ellner, S. & Hairston, N.G. (1994). Role of overlapping generations in maintaining genetic variation in a fluctuating environment. *The American Naturalist*, 143, 403-417.

Gillespie, J.H. (1977). Natural selection for variances in offspring numbers: a new evolutionary principle. *The American Naturalist*, 111, 1010-1014.

Gremer, J.R. & Venable, D.L. (2014). Bet hedging in desert winter annual plants: optimal germination strategies in a variable environment. *Ecol Lett*, 17, 380-387.

Gulisija, D. & Kim, Y. (2015). Emergence of long-term balanced polymorphism under cyclic selection of spatially variable magnitude. *Evolution*, 69, 979-992.

Gulisija, D., Kim, Y. & Plotkin, J.B. (2016). Phenotypic plasticity promotes balanced polymorphism in periodic environments by a genomic storage effect. *Genetics*, 202, 1437-1448.

Hedrick, P.W. (1986). Genetic polymorphism in heterogeneous environments: a decade later. *Annual Review of Ecology and Systematics* 17:535-566.

Hedrick, P.W., Ginevan, M.E. & Ewing, E.P. (1976). Genetic polymorphism in heterogeneous environments. *Annual Review of Ecology and Systematics* 7:1-33.

Johnson O.L, Tobler R, Schmidt J.M., Huber C.D. (2023). Fluctuating selection and the determinants of genetic variation. *Trends in Genetics,* 39, 491-504

Kim, Y. (2023). Partial protection from fluctuating selection leads to evolution towards wider population size fluctuation and a novel mechanism of balancing selection. *Proceedings of the Royal Society B*, 290, 20230822.

Liu, M., Rubenstein, D.R., Liu, W.-C. & Shen, S.-F. (2019). A continuum of biological adaptations to environmental fluctuation. *Proceedings of the Royal Society B*, 286, 20191623.

Lynch, M., Wei, W., Ye, Z. & Pfrender, M. (2024). The genome-wide signature of short-term temporal selection. *Proceedings of the National Academy of Science USA*, 121:e2307107121.

Messer, P.W., Ellner, S.P. & Hairston, N.G. (2016). Can population genetics adapt to rapid evolution? *Trends in Genetics*, 32, 408-418.

Myers, J.H. (1988). Can a general hypothesis explain population cycles of forest Lepidoptera? In: *Advances in Ecological Research*. Elsevier, pp. 179-242.

Park Y, Kim Y. 2019. Partial protection from cyclical selection generates a high level of polymorphism at multiple non-neutral sites. *Evolution*, 73, 1564-1577.

Pfister, C.A. (1998). Patterns of variance in stage-structured populations: evolutionary predictions

and ecological implications. *Proceedings of the National Academy of Sciences*, 95, 213-218.
Reed, T.E., Waples, R.S., Schindler, D.E., Hard, J.J. & Kinnison, M.T. (2010). Phenotypic plasticity and population viability: the importance of environmental predictability. *Proceedings of the Royal Society B: Biological Sciences*, 277, 3391-3400.
Reinhold, K. (2000). Maintenance of a genetic polymorphism by fluctuating selection on sex-limited traits. *Journal of Evolutionary Biology*, 13, 1009-1014.
Slatkin, M. (1974). Hedging one's evolutionary bets. *Nature*, 250, 704-705.
Thompson, J.N. (2013). *Relentless evolution*. University of Chicago Press.
Tuljapurkar, S. & Orzack, S.H. (1980). Population dynamics in variable environments I. Long-run growth rates and extinction. *Theoretical Population Biology*, 18, 314-342.
Tuljapurkar, S.D. (1982). Population dynamics in variable environments. III. Evolutionary dynamics of r-selection. *Theoretical population biology*, 21, 141-165.
Turelli, M., Schemske, D.W. & Bierzychudek, P. (2001). Stable two-allele polymorphisms maintained by fluctuating fitnesses and seed banks: protecting the blues in Linanthus parryae. *Evolution*, 55, 1283-1298.
Wallace, B. (1975). Hard and soft selection revisited. *Evolution*, 465-473.
White, T. (2008). The role of food, weather and climate in limiting the abundance of animals. *Biological Reviews*, 83, 227-248.
Yamamichi, M., Letten, A.D. & Schreiber, S.J. (2023). Eco-evolutionary maintenance of diversity in fluctuating environments. *Ecology Letters*, 26, S152-S167.
Zhang, J. (2023). Patterns and evolutionary consequences of pleiotropy. *Ann Rev Ecol Evol Syst* 54:1-19.

Appendix A. Derivation of conditions for balancing and directional selection on A_2 in the TP model

Here only deterministic changes in allele frequencies are tracked as a very large population is assumed. At the end of reproduction but before migration into subpopulations at generation t - 1, there are n_1 and n_2 copies (individuals) of A_1 and A_2 in the entire population. After migration, the numbers of A_1 and A_2 copies are $n_{1F} = (1-r)n_1$ and $n_{2F} = (1-r)n_2$ in the field and $n_{1R} = rn_1$ and $n_{2R} = rn_2$ in the refuge. Therefore, the relative frequencies of alleles are identical between the field and refuge before reproduction at the beginning of generation t.

Soft selection is first considered. Then, each A_2 allele produces $(1+S_t)K_F^{(t)}/\bar{N}_F^{(t)}$ copies of descendants, where $\bar{N}_F^{(t)} = n_{1F} + (1+S_t)n_{2F} = (1-r)(n_1 + (1+S_t)n_2)$. Since $n_1 \gg n_2$ after A_2 appears by mutation, $n_1 + (1+S_t)n_2 \approx n_1$ and $\bar{N}_F^{(t)} \approx \tilde{N}_F^{(t)} = (1-r)(n_1 + n_2) = (1-r)\left(K_F^{(t-1)} + K_R^{(t-1)}\right)$. Similar approximations apply to the refuge. Let n'_2 be the expected number of A_2 in generation t. This leads to eq. (1). Namely,

$$\frac{n'_2}{n_2} = (1-r)\frac{(1+S_t)K_F^{(t)}}{\bar{N}_F^{(t)}} + r\frac{(1+S_t^*)K_R^{(t)}}{\bar{N}_R^{(t)}} \approx (1-r)\frac{(1+S_t)K_F^{(t)}}{\tilde{N}_F^{(t)}} + r\frac{(1+S_t^*)K_R^{(t)}}{\tilde{N}_R^{(t)}} = \frac{(1+S_t)K_F^{(t)} + (1+S_t^*)K_R^{(t)}}{K_F^{(t-1)} + K_R^{(t-1)}}$$

Then, with $K_F^{(t-1)} = K_{F0}\mathrm{e}^U$, $K_R^{(t-1)} = K_{R0}\mathrm{e}^V$, $K_F^{(t)} = K_{F0}\mathrm{e}^{U'}$, and $K_R^{(t)} = K_{R0}\mathrm{e}^{V'}$,

$$\Delta_2 \equiv \log\left[\frac{n'_2}{n_2}\right] = \log\left[\mathrm{e}^{U'}(1+S) + \rho\mathrm{e}^{V'}(1+S^*)\right] - \log\left[\mathrm{e}^U + \rho\mathrm{e}^V\right],$$

where $S = S_t$, $S^* = S_t^*$, and $\rho = K_{R0}/K_{F0}$. If $\mathrm{E}[\Delta_2] > 0$, where the expectation is over demographic and fitness fluctuations, the rare allele A_2 is expected to increase its copy number over time. As the expectations of U, V, U', V', S, and S^* are all zero and non-zero correlations exist only among U', V', S, and S^* and between U and V, from the Taylor expansion of Δ_2 up to the second order, it can be shown that

$$\begin{aligned}\mathrm{E}[\Delta_2] \approx {} & \frac{1}{2}\alpha_{U'U'}\mathrm{Var}[U'] + \frac{1}{2}\alpha_{V'V'}\mathrm{Var}[V'] + \frac{1}{2}\alpha_{UU}\mathrm{Var}[U] + \frac{1}{2}\alpha_{VV}\mathrm{Var}[V] + \frac{1}{2}\alpha_{SS}\mathrm{Var}[S] \\ & + \frac{1}{2}\alpha_{S^*S^*}\mathrm{Var}[S^*] + \alpha_{U'V'}\mathrm{Cov}[U',V'] + \alpha_{UV}\mathrm{Cov}[U,V] + \alpha_{SS^*}\mathrm{Cov}[S,S^*] \\ & + \alpha_{SU'}\mathrm{Cov}[S,U'] + \alpha_{SV'}\mathrm{Cov}[S,V'] + \alpha_{S^*U'}\mathrm{Cov}[S^*,U'] + \alpha_{S^*V'}\mathrm{Cov}[S^*,V']\end{aligned}$$

where $\alpha_{XY} = \frac{\partial^2\Delta_2}{\partial X \partial Y}$ is the Taylor coefficient for variables X and Y evaluated at $(U,V,U',V',S,S^*) = (0,0,0,0,0,0)$. Since $\alpha_{U'U'} = \alpha_{V'V'} = \alpha_{UV} = \alpha_{SU'} = \alpha_{S^*V'} = \frac{\rho}{(1+\rho)^2}$, $\alpha_{UU} = \alpha_{VV} = \alpha_{U'V'} = \alpha_{SV'} = \alpha_{S^*U'} = \alpha_{SS^*} = -\frac{\rho}{(1+\rho)^2}$, $\alpha_{SS} = -\frac{1}{(1+\rho)^2}$, $\alpha_{S^*S^*} = -\frac{\rho^2}{(1+\rho)^2}$, $\mathrm{Var}[U] = \mathrm{Var}[U']$, $\mathrm{Var}[V] = \mathrm{Var}[V']$, and $\mathrm{Cov}[U,V] = \mathrm{Cov}[U',V']$, the above equation is simplified to

$$\mathrm{E}[\Delta_2] = \frac{2\rho\mathrm{Cov}[S-S^*, U'-V'] - \mathrm{Var}[S] - \rho^2\mathrm{Var}[S^*] - 2\rho\mathrm{Cov}[S,S^*]}{2(1+\rho)^2}.$$

Therefore, the condition for the A_2 allele being positively selected (i.e. invading the population initially fixed for A_1), is $\mathrm{E}[\Delta_2] > 0$ or

$$\frac{\mathrm{Cov}[S-S^*, U'-V']}{\mathrm{Var}[S] + \rho^2\mathrm{Var}[S^*] + 2\rho\mathrm{Cov}[S,S^*]} > \frac{1}{2\rho}$$

assuming $(\mathrm{Var}[S]+\rho^2\mathrm{Var}[S^*])/\rho > -2\mathrm{Cov}[S,S^*]$. If $S_t^* = \delta S_t$, the above condition becomes

$$\Phi = \frac{\mathrm{Cov}[S, U'-V']}{\mathrm{Var}[S]} = \frac{\mathrm{Cov}[S, \log[K_F/K_R]]}{\mathrm{Var}[S]} > \frac{(1+\delta\rho)^2}{2(1-\delta)\rho}.$$

A similar result can be obtained for the condition for the A_1 allele to invade a population initially fixed for A_2. The expected change in the number of A_1, n'_1/n_1, is obtained with a procedure identical to that leading to eq. (1) except that the relative fitness is flipped. Therefore

$$\frac{n_1'}{n_1} \approx \frac{K_F^{(t)}}{K_F^{(t-1)}+K_R^{(t-1)}}\frac{1}{1+S_t} + \frac{K_R^{(t)}}{K_F^{(t-1)}+K_R^{(t-1)}}\frac{1}{1+S_t^*} = \frac{(1+S_t^*)\mathrm{e}^{U'}+(1+S_t)\rho\mathrm{e}^{V'}}{(1+S_t)(1+S_t^*)(\mathrm{e}^{U}+\rho\mathrm{e}^{V})}$$

for $n_1 \ll n_2$, the dynamics of the rare A_1 allele depends on the expectation of

$$\Delta_{1,\mathrm{soft}} \equiv \log\left[\frac{n_1'}{n_1}\right] = \log\left[(1+S_t^*)\mathrm{e}^{U'} + (1+S)\rho\mathrm{e}^{V'}\right] - \log[(1+S)(1+S_t^*)(\mathrm{e}^{U}+\rho\mathrm{e}^{V})].$$

Applying the quadratic approximation used for obtain $\mathrm{E}[\Delta_2]$ above, one obtains

$$\mathrm{E}\left[\Delta_{1,\mathrm{soft}}\right] = \frac{(1+2\rho)\mathrm{Var}[S]+(2\rho+\rho^2)\mathrm{Var}[S^*]-2\rho\mathrm{Cov}[S,S^*]-2\rho\mathrm{Cov}[S-S^*, U'-V']}{2(1+\rho)^2}$$

The condition for the rare A_1 allele to increase in frequency is $\mathrm{E}\left[\Delta_{1,\mathrm{soft}}\right] > 0$ or

$$\frac{\mathrm{Cov}[S-S^*, U'-V']}{(1+2\rho)\mathrm{Var}[S]+(2\rho+\rho^2)\mathrm{Var}[S^*]-2\rho\mathrm{Cov}[S,S^*]} < \frac{1}{2\rho}.$$

If $S_t^* = \delta S_t$, the above condition becomes

$$\Phi < 1-\delta+\frac{(1+\delta\rho)^2}{2(1-\delta)\rho}.$$

With hard selection, the frequency change of A_2 when rare is again given by eq. (1). Therefore, the condition for A_2 invading the population fixed for A_1 ($\mathrm{E}[\Delta_2] > 0$ above) is no different from that for soft selection. However, because the carrying capacities of the field and refuge fixed for A_2 at generation t is given by $(1+S_t)K_F^{(t)}$ and $(1+S_t^*)K_R^{(t)}$ and $\tilde{N}_F^{(t)} = (1-r)((1+S_{t-1})K_F^{(t-1)} + (1+S_{t-1}^*)K_R^{(t-1)})$ and $\tilde{N}_R^{(t)} = r((1+S_{t-1})K_F^{(t-1)} + (1+S_{t-1}^*)K_R^{(t-1)})$ under hard selection, the opposite condition for A_1 invading A_2 is given by

$$\frac{n_1'}{n_1} = (1-r)\frac{(1+S_t)K_F^{(t)}}{\tilde{N}_F^{(t)}}\frac{1}{1+S_t} + r\frac{(1+S_t^*)K_R^{(t)}}{\tilde{N}_R^{(t)}}\frac{1}{1+S_t^*} = \frac{K_F^{(t)}+K_R^{(t)}}{(1+S_{t-1})K_F^{(t-1)}+(1+S_{t-1}^*)K_R^{(t-1)}} = \frac{e^{U'}+\rho\mathrm{e}^{V'}}{(1+S_{t-1})e^{U}+(1+S_{t-1}^*)\rho e^{V}}$$

Then,

$$\Delta_{1,\mathrm{hard}} \equiv \log\left[\frac{n_1'}{n_1}\right] = \log\left[\mathrm{e}^{U'} + \rho\mathrm{e}^{V'}\right] - \log\left[(1+S_{t-1})\mathrm{e}^{U} + (1+S_{t-1}^{*})\rho\mathrm{e}^{V}\right].$$

Now, given that (U', V', S_t, S_t^*) and $(U, V, S_{t-1}, S_{t-1}^*)$ are independent samples of a joint distribution, the expectation of $-\Delta_{1,\mathrm{hard}}$ is exactly that of Δ_2 above. Therefore, the condition for the fixation of A_2 ($\mathrm{E}[\Delta_{1,\mathrm{hard}}] < 0$) is exactly the condition for positive selection on a rare A_2 ($\mathrm{E}[\Delta_2] > 0$).

Appendix B. Methods of stochastic simulation

Stochastic simulations of the single- and multi-locus eco-evolutionary models were implemented in Mathematica ver. 11.3. In both TP and LSA models, an individual has L loci that are arranged linearly with probability c for recombination between adjacent loci per generation. The simulation keeps track of two sets of lists: one containing the sets of haplotypes carried by non-zero individuals in the field and in the refuge ($\boldsymbol{H}_{\mathrm{F}}$ and $\boldsymbol{H}_{\mathrm{R}}$) and the other containing the corresponding numbers of individuals ($\boldsymbol{N}_{\mathrm{F}}$ and $\boldsymbol{N}_{\mathrm{R}}$).

In the TP model, generation t begins after the migration step (re-distribution of individuals produced in the previous generation into the field and refuge) is completed. Haplotype numbers are first updated by mutation and recombination. A Poisson variate with mean $NL\mu$, where N is the current size of a subpopulation at hand, is drawn as the total number of mutation events and each event is assigned to haplotypes and loci proportional to their current frequencies. Similarly, for $L > 1$, a Poisson variate with mean $N(L\text{-}1)c$ is drawn as the total number of recombination events in the subpopulation. For each recombination event, two haplotypes are chosen proportional to their frequencies and one of the L-1 intervals is chosen to be the position of crossing-over between the two haplotypes. Newly created or lost haplotypes during mutation and recombination steps are tracked by updating ($\boldsymbol{H}_{\mathrm{F}}$, $\boldsymbol{H}_{\mathrm{R}}$) and ($\boldsymbol{N}_{\mathrm{F}}$, $\boldsymbol{N}_{\mathrm{R}}$). Then, the updated lists are further updated in the step of reproduction according to their fitness. Under soft selection, the number of individuals with haplotype i born in the next generation is Poisson distributed with mean $W_i \tilde{N}_{F,i}^{(t)} K_F^{(t)} / \sum_j W_j \tilde{N}_{F,j}^{(t)}$ in the field and $\tilde{N}_{R,i}^{(t)} K_R^{(t)} / \sum_j \tilde{N}_{R,j}^{(t)}$ in the refuge, where W_i is the relative fitness of haplotype i and $\tilde{N}_{F,i}^{(t)}$ ($\tilde{N}_{R,i}^{(t)}$) is the number of haplotype i individuals in the field (refuge) after the haplotypes are updated by mutation and recombination. Under hard selection, the corresponding mean number in the field is $W_i \tilde{N}_{F,i}^{(t)} K_F^{(t)} / \sum_j \tilde{N}_{F,j}^{(t)}$. Finally, when there are N_{F} and N_{R} individuals in the field and refuge, Poisson variates with the mean $N_{\mathrm{F}} m_{\mathrm{FR}}$ ($N_{\mathrm{R}} m_{\mathrm{RF}}$) truncated

at the upper bound N_F (N_R) is drawn to determine the numbers of migrants from field to refuge (from refuge to field). Migrants are chosen proportional to the haplotype frequencies and then merged with the residents of the destination. This completes a single-generation iteration in updating haplotype lists.

Simulation for the LSA model is similarly performed by tracking the haplotypes. Let l_i, m_i, and n_i be the number of individuals with haplotype i (i = 1, ..., 2^L) at the larval, subadult, and adult stages at time t. The following steps are taken to produce the corresponding numbers, l_i', m_i', and n_i', at time t+1. As in the TP model, bidirectional mutations with probability μ per locus and recombination with probability c for adjacent loci occur but in subadult and adult stages only. Recombination must occur among subadults and among adults only. The updated numbers of individuals by haplotypes in subadults and adults after mutations and recombination are m_i^*, and n_i^*. Next, the numbers of individuals by haplotypes in the next generation are obtained. l_i' is given by a Poisson number with mean $W_i^{(t)}(m_i^* + n_i^*)K_L^{(t)}/\sum_i(W_i^{(t)}(m_i^* + n_i^*))$ under soft selection and $W_i^{(t)}(m_i^* + n_i^*)K_L^{(t)}/\sum_i(m_i^* + n_i^*)$ under hard selection. m_i' and n_i' are given by Poisson numbers with mean $e_L l_i$ and $e_L e_S m_i^*$, respectively.

Table S1. One-locus simulation with variable migration rates in the TP model

mode	m_{FR}	m_{RF}	$\overline{H}$	P_{01}	P_{90}
neutral ($a_S = b_S = 0$, $a_U =$ 0.3, $a_V = 0.05$, $b_U = b_V = 0.1$)	[0.495, 0.505]	[0.495, 0.505]	0.0633	0.60	0.24
	[0.1, 0.2]	[0.1, 0.2]	0.0657	0.61	0.24
soft selection, Φ = 1 ($a_S = 0.2$, $b_S = 0.1$, a_U =0.3, $a_V = 0.05$, $b_U = b_V = 0.1$)	[0.495, 0.505]	[0.495, 0.505]	0.196	0.28	0.25
	[0.3, 0.7]	[0.3, 0.7]	0.177	0.45	0.14
	[0.3, 0.5]	[0.5, 0.7]	0.135	0.42	0.26
	[0.5, 0.7]	[0.3, 0.5]	0.147	0.42	0.25
	[0.1, 0.5]	[0.1, 0.5]	0.142	0.59	0.08
	[0.2, 0.4]	[0.2, 0.4]	0.118	0.70	0.03
	[0.1, 0.2]	[0.1, 0.2]	0.056	0.88	0.00
	[0.1. 0.2]	[0.4, 0.5]	0.186	0.34	0.22
	[0.4, 0.5]	[0.1, 0.2]	0.052	0.88	0.01
Hard selection, Φ = 0.8 ($a_S = 0.2$, $b_S = 0.15$, a_U =0.3, $a_V = 0.05$, $b_U = b_V = 0.1$)	[0.495, 0.505]	[0.495, 0.505]	0.030	0.05	0.89
	[0.3, 0.7]	[0.3, 0.7]	0.020	0.01	0.95
	[0.2, 0.4]	[0.2, 0.4]	0.074	0.45	0.39
	[0.1, 0.2]	[0.1, 0.2]	0.034	0.93	0.00
	[0.1. 0.2]	[0.4, 0.5]	0.038	0.02	0.90
	[0.4, 0.5]	[0.1, 0.2]	0.036	0.91	0.01

Table S2. Mean time to the fixation (q > 0.9) of A_2 in the multi-locus simulation of the TP model

mode	L	c	$\bar{T}$	c. v.*
soft selection, $\Phi = 2$ ($a_S = 0.1$, $b_S = 0$, $a_U = 0.25$, $a_V = 0.05$, $b_U = b_V = 0.05$)	1	-	5863	-
	5	10^{-4}	9287	0.66 ± 0.21
	5	10^{-3}	8212	0.67 ± 0.20
	5	0.01	8621	0.70 ± 0.26
	5	0.1	8162	0.65 ± 0.26
hard selection, $\Phi = 0.75$ ($a_S = 0.2$, $b_S = 0$, $a_U = 0.25$, $a_V = 0.1$, $b_U = b_V = 0.05$)	1	-	6242	-
	5	10^{-4}	3776	0.34 ± 0.17
	5	10^{-3}	3228	0.32 ± 0.16
	5	0.01	3030	0.31 ± 0.20
	5	0.1	3459	0.28 ± 0.19
hard selection, $\Phi = 1$ ($a_S = 0.15$, $b_S = 0$, $a_U = 0.15$, $b_U = 0.05$, $a_V = 0$, $b_V = 0.1$)	1	-	5177	-
	5	10^{-4}	4648	0.35 ± 0.15
	5	10^{-3}	4018	0.35 ± 0.17
	5	0.01	3969	0.32 ± 0.20
	5	0.1	3467	0.37 ± 0.21

*Coefficient of variation for 5 consecutive times (T_1, ..., T_5) observed in a single simulation run, averaged over replicates (± standard deviation).

All results are based on 100 replicates for each parameter set

Table S3. Long-term polymorphism in multi-locus simulations of the LSA model

mode	a_S	b_S	Φ'	$\overline{H}$	P_{01}	P_{90}
neutral	0	0	-	0.0757	0.42	0.40
soft selection	0.05	0.2	0.294	0.013	1	0
	0.1	0.2	0.5	0.017	0.96	0
	0.1	0.15	0.769	0.040	0.91	0.00
	0.2	0.1	1	0.152	0.63	0.08
	0.2	0	1.25	0.116	0.31	0.41
	0.15	0.05	1.5	0.054	0.17	0.71
	0.1	0.05	2	0.023	0.22	0.73
hard selection	0.05	0.2	0.294	0.006	0.99	0
	0.1	0.2	0.5	0.013	0.97	0
	0.1	0.15	0.769	0.009	0.22	0.76
	0.2	0.1	1	0.003	0.07	0.93
	0.15	0.087	1.25	0.003	0.24	0.76
	0.15	0.05	1.5	0.005	0.06	0.92

- Each simulation ran for 50,000 time units, except for the neutral simulation that ran 500,000 time units.

- Other parameters: $\mu = 2\times10^{-5}$, $K_{L0} = 5000$, $a_L = 0.25$, $b_L = 0.1$, $e_L = 0.7$, $e_S = 0.7$, $L = 4$, $c = 0.03$.

Figure S1

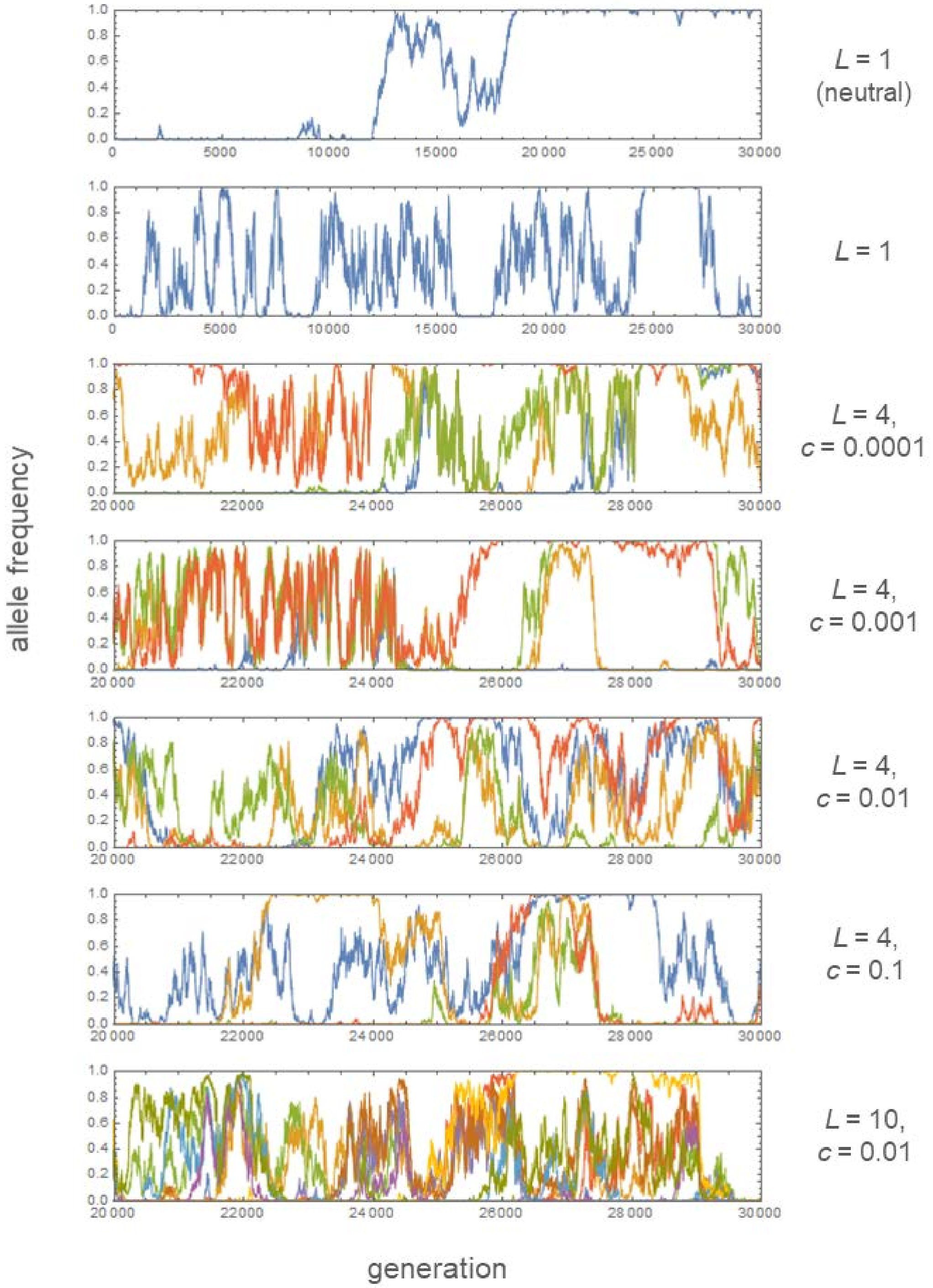

Figure S1 legend: Exemplary trajectories of allele (A_2) frequencies in simulations. Allele frequencies are plotted in different colors for different loci. The number of loci (L) and recombination rate (c) are shown on the right side of graphs. Other parameters: $K_{R0} = K_{F0} = 1000$, $\mu = 2\times10^{-5}$, $\Phi = 1$ ($a_S = 0.2$ [0 for neutral], $b_S = 0.1$, $a_U = 0.3$, $b_U = 0.1$, $a_V = 0.05$, $b_V = 0.1$).

Figure S2

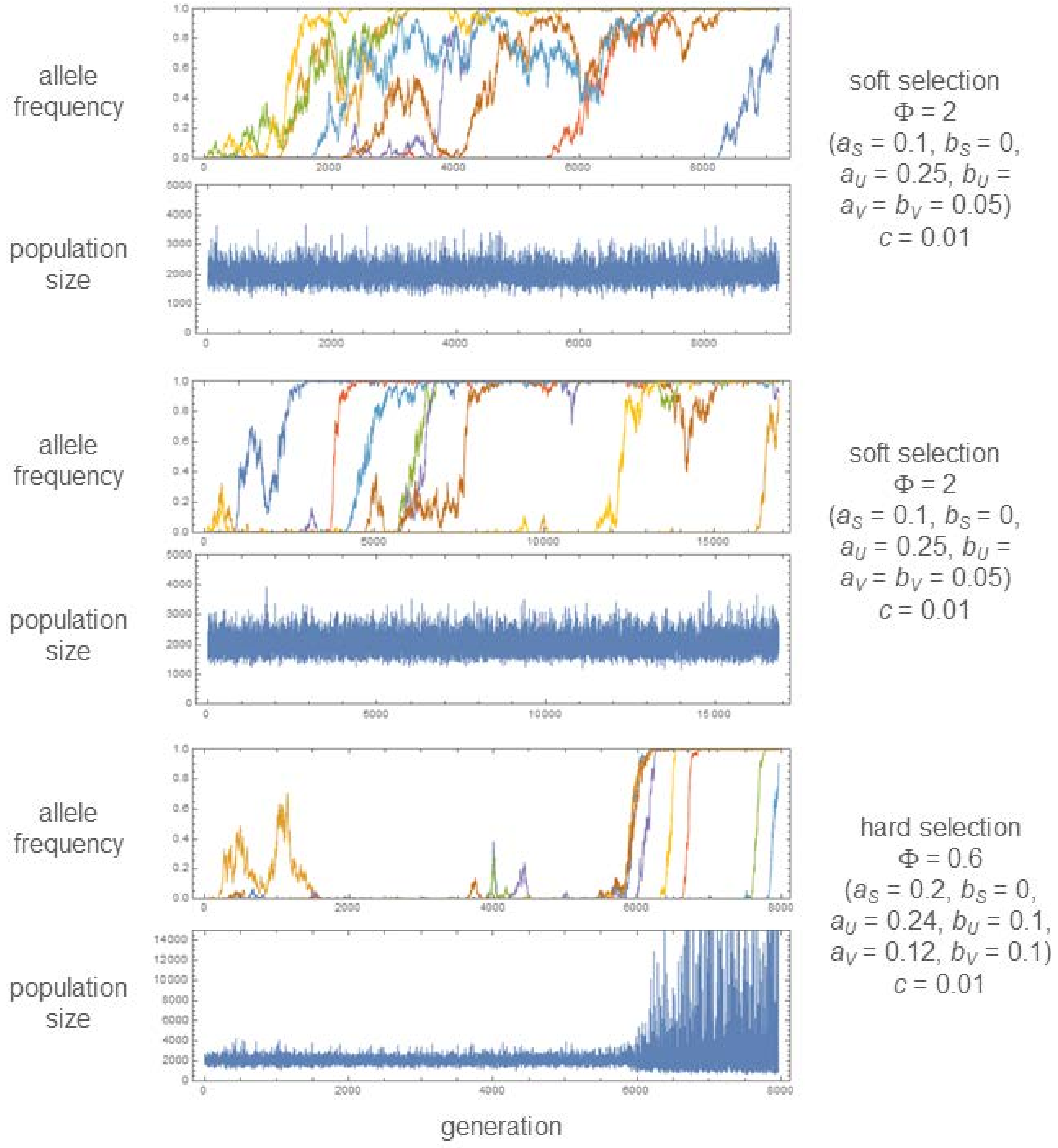

allele frequency
population size
soft selection
Φ = 2
(a_S = 0.1, b_S = 0,
a_U = 0.25, b_U =
a_V = b_V = 0.05)
c = 0.01
allele frequency
population size
soft selection
Φ = 2
(a_S = 0.1, b_S = 0,
a_U = 0.25, b_U =
a_V = b_V = 0.05)
c = 0.01
allele frequency
population size
hard selection
Φ = 0.6
(a_S = 0.2, b_S = 0,
a_U = 0.24, b_U = 0.1,
a_V = 0.12, b_V = 0.1)
c = 0.01
generation

Figure S2 (continued)

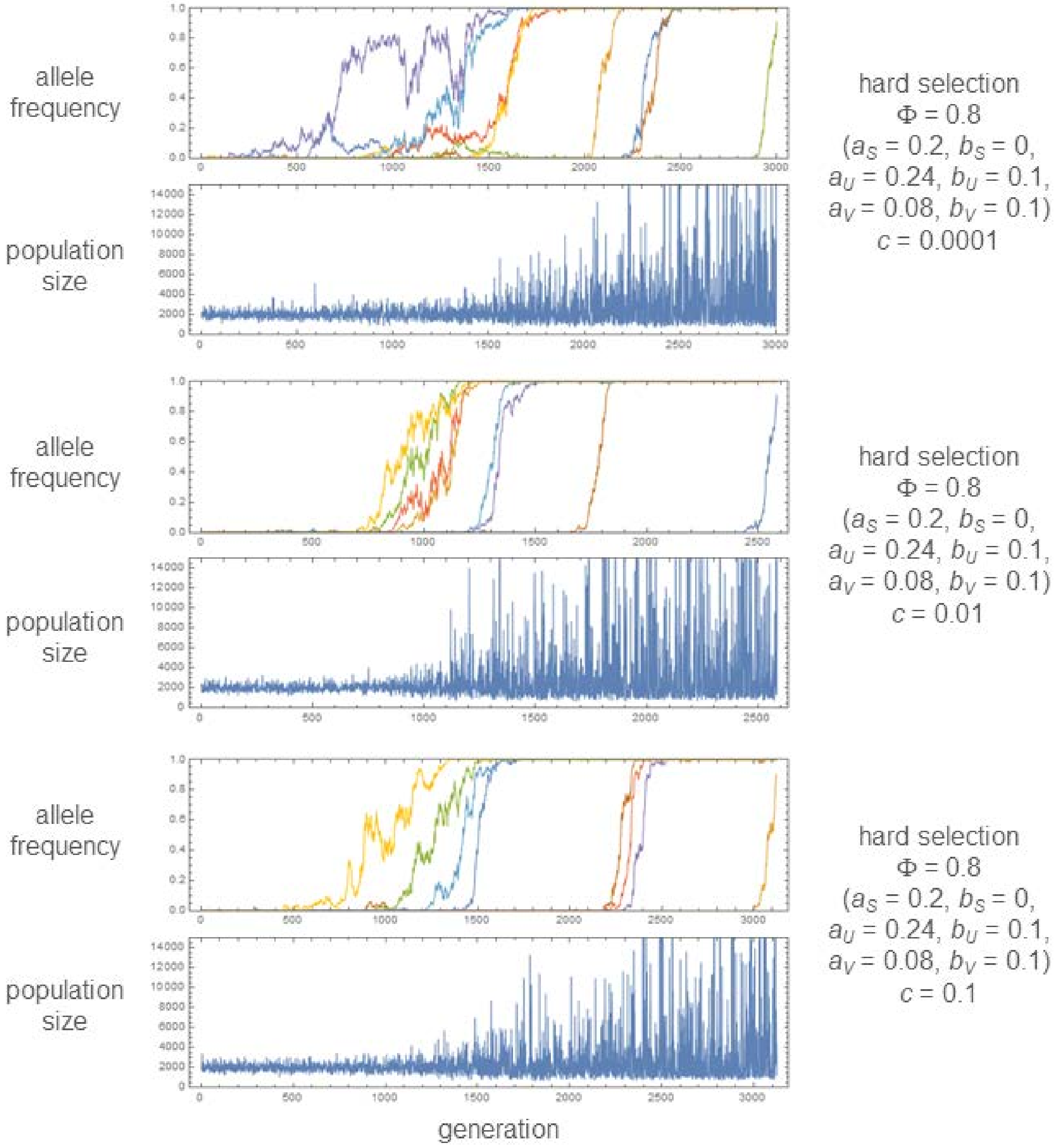

Figure S2 legend: Examples of simulation runs in which the mutant allele (A_2) reaches fixation sequentially at all 8 loci used in the TP model. The change of population size is shown below that of allele frequencies, plotted in different colors for different loci. Parameter values are shown on the right side of graphs. Other parameters: K_{R0} = K_{F0} = 1000, μ = 2×10^{-5}.

Figure S3

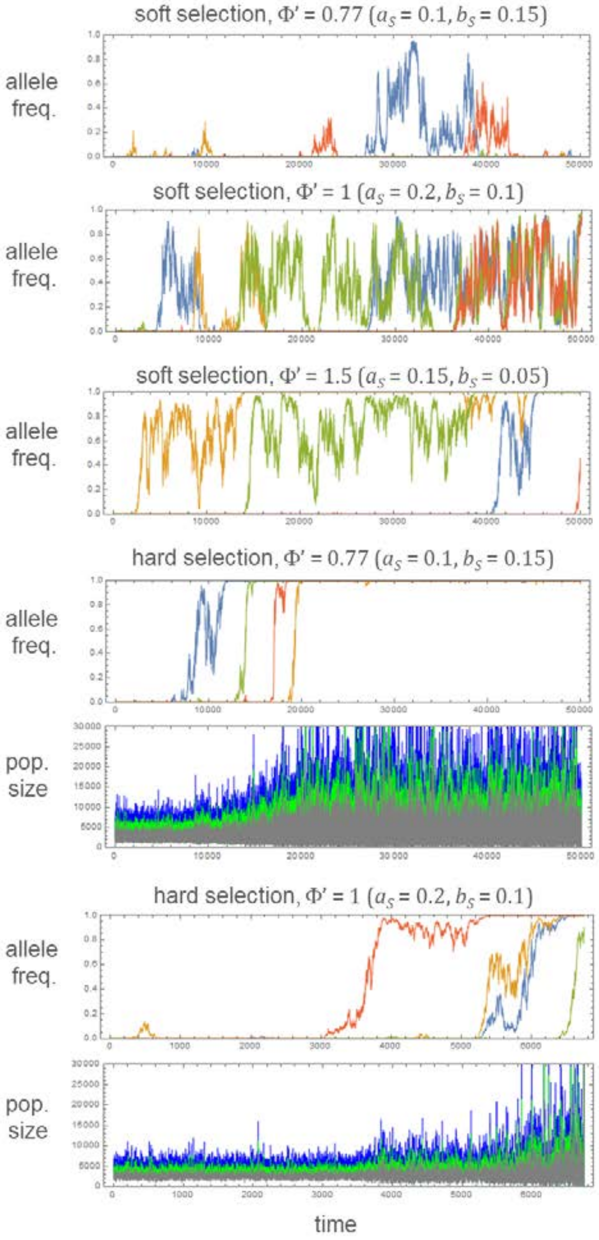

Figure S3 Legend: Exemplary allele frequency changes in the simulation of the LSA model. Each simulation started with a population fixed for the A_1 allele and ran for 50,000 time units, except for the case of hard selection with Φ' = 1. The A_2 allele appears by recurrent mutations at each of L = 4 loci. Allele frequencies are plotted in different colors for different loci. For hard selection, the change of population size (blue: larval, green: subadult, gray: adult) is shown below the allele frequency trajectories. Other parameters: K_{L0} = 5000, a_L = 0.25, b_L = 0.1, e_L = e_S = 0.7, μ = 2×10^{-5}, c = 0.03.

Figure S4

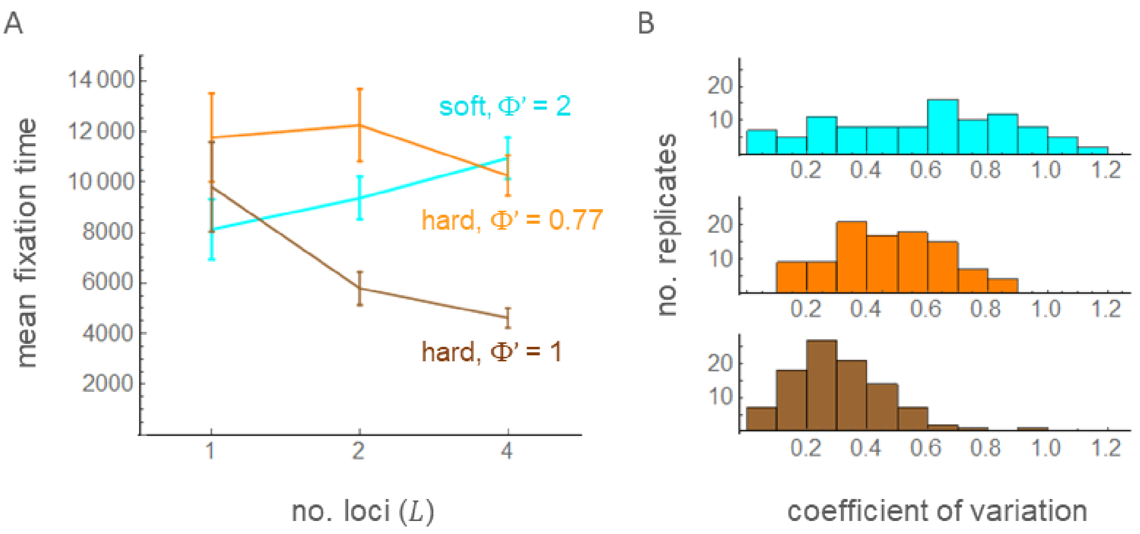

Figure S4 legend: Mean fixation time ($\bar{T}$) with varying number of loci (A; L = 1, 2, and 4) and the coefficient of variation in fixation times with L = 4 in the multi-locus simulation of the LSA model. Error bars show two times the standard errors. A total of 100 replicates of simulations were run under soft selection with Φ’ = 2 (cyan; a_S = 0.1, b_S = 0.05, a_L = 0.25, b_L = 0.1, μ = 3×10^{-5}), hard selection with Φ’ = 1 (brown; a_S = 0.2, b_S = 0.1, a_L = 0.25, b_L = 0.1, μ = 2×10^{-5}), or hard selection with Φ’ = 0.77 (orange; a_S = 0.1, b_S = 0.15, a_L = 0.25, b_L = 0.1, μ = 3×10^{-5}). Other parameters are identical to those in Figure S3.

Response to reviewer comments (PONE-D-24-54939)

I revised the manuscript following the comments provided by two reviewers. Below each review comment (in italic) is followed by my response. Line numbers are those of revised manuscript.

Yuseob Kim
Ewha Womans University

Editor’s comment:

While both reviewers agree that there is some merit in the article reviewer#1 suggests a change on the pitch of the article, while reviewer #2 founds the current version unsuitable for publication. As I found the reviewers comments constructive, I would like to ask you to revise your manuscript. However, this will be a major revision, with substantial changes required. Particularly the language needs to be adjusted for better readability and the mathematics must be sound. As reviewer #2 suggests rejection, the manuscript must be a substantially improvement, with a clear presentation and rigorous mathematics. Reviewer #2 mentions that some frustration is accompanying the manuscript. However, I believe that both reviewers make constructive comments that will guide a substantial revision, which should be regarded an opportunity rather than a cause for more frustration.

>> I greatly appreciate your decision to allow me to revise and resubmit the manuscript. I revised manuscripts to address all comments by both reviewers. I tried to revise texts in Abstract, Introduction, and Discussion to improve readability and remove confusions.

Reviewers' comments:

Reviewer #1: The author studies the maintenance of variation under fluctuating selection. Using two different population models, two-patch model and larval-subadult-adult model with hard and soft selection. The work shown in this manuscript is a generalization of previous results of the author. While the analyses are complete and convincing, the reviewer is concerned about the pitch of the manuscript.My understanding is that variation is maintained only under a set of very restricted conditions, hence the focus should be on the difficulty to maintain variation under fluctuating selection, rather than portraying it as a realistic scenario. As written, the reviewer feels that the study may be cited for evidence that variation is maintained under fluctuating selection, although most results show the opposite. It may also help to make more effort to discuss how biologically reasonable the permissive parameter space is. In other words, how likely is it that these conditions are met in natural populations?

>> This aspect of the result – in larger parameter space the mutant alleles under fitness fluctuations are positively selected to fixation rather than maintained in polymorphism – was indeed not discussed in depth. I agree that it appears to suggest that the new version of storage effect affected by demographic fluctuations, the noncovarying storage effect (NSE), fails to generate balancing selection. Now I state "One may therefore regard the population size oscillation as a factor that destroys the potential of storage effect to generate balanced polymorphism." (l. 111)

However, whether there will be more or less balanced polymorphism in nature due to the NSE depends on what kinds of mutants with their own patterns of fluctuating fitness will be available for the NSE to act on. As pointed out in Kim (2023) and this study, the previous models of the storage effect (without population size fluctuation) can maintain polymorphism only when mutants have identical geometric mean fitness to the wild-type allele (thus GMF mutants), which is possible only if its arithmetic mean fitness is greater than the wild-type. NSE leads to either loss or fixation of GMF mutants. However, the NSE produces balanced polymorphism due to AMF mutations that confer the same arithmetic mean fitness as the wild-type. Therefore, given the assumption that AMF mutations are more likely to occur than GMF mutations, which I believe is probably true, population size fluctuation, thus NSE, makes it more plausible to have balanced polymorphism in the genome than the previous theories of the storage effect predicted. I therefore argue in the Introduction that "However, this population size oscillation led to balancing selection on a different type of mutants — alleles conferring smaller geometric mean but identical arithmetic mean fitness compared to the wild-type. Since such AMF (arithmetic-mean-preserving fitness fluctuation) mutations, which are deleterious in a simple population (without a refuge), are probably more likely to occur in the genome than the GMF mutations, this version of storage effect modified by demographic fluctuation might be equally important or even more important in generating balanced polymorphism in nature." (l.113-119).

Furthermore, even in the parameter space in which AMF mutants are not maintained as polymorphism but reach fixation, namely the cases of stronger demographic fluctuation relative to fitness fluctuation, there can be mutants whose fluctuation in fitness is further different from the AMF that is positively selected only when they are in low frequency so that they are maintained as polymorphism. Those mutations are likely to have net arithmetic mean fitness LOWER than that of an AMF allele, which even more plausible to occur in the genome. For this reason, we may not say that balanced polymorphism is less likely under fluctuating selection due to the NSE. This argument, using the heuristic explanation for the NSE, is given in the Discussion: "Because fixation rather than balanced polymorphism occurs in a wider parameter space, one may argue that the overall effect of NSE (i.e. the modification of the storage effect due to population size fluctuation) is to make balancing selection less likely to occur. However, given the possible patterns of mutant alleles' fitness fluctuations in nature beyond those considered so far, the NSE may be a force of increasing, rather than decreasing, the number of loci in which oscillatory polymorphism occurs. First, the NSE causes all GMF mutants to be either lost or fixed in the population, thus no balanced polymorphism (Kim 2023), but allows AMF mutants to be maintained as balanced polymorphism. As one may expect more AMF mutations than GMF mutations to occur in the genome, since more beneficial mutations (in net effect) are considered less likely in general, the number of loci under balancing selection may increase due to the NSE. This also suggests that for a given demographic fluctuation, mutations of certain fluctuation in

fitness are "picked up" to be maintained as polymorphism. In the heuristic argument above, the absolute fitness of the A_2 allele in the field is given by $W_{2F} = D_F w_2$, where D_F is its absolute fitness given by change in the population size of the field alone and w_2 is the relative fitness of A_2 in the field (e.g., in the TP model, $D_F = \frac{K_F^{(t)}}{\tilde{N}_F^{(t)}}$ and $w_2 = 1 + S_t$). It is a limited range of fluctuation in W_{2F} that produce polymorphism, as suggested in the above model with AMF mutations where a larger fluctuation in population size (thus in D_F) allows polymorphism only when the mutant alleles have a larger fluctuation in fitness relative to the wild-type (so that $\mathrm{Var}[S]$ matches $\mathrm{Cov}[S, \log[K_F/K_R]]$), which makes the arithmetic mean of W_{2F} decline. It might be possible that for a strong demographic fluctuation under which an AMF mutant is driven to fixation, a mutant with a new pattern of fitness fluctuation not considered in this study so far, possibly with even lower arithmetic mean than AMF mutants (thus < 1), might be picked up by the NSE into balanced polymorphism." (l. 574-596).

Reviewer #2:
The present article spans the fields of population genetics and ecology and, thus, should be considered interdisciplinary. The file includes comments of previous reviewers and the reply of the author. It seems that the reviewers as well as the author were quite frustrated. I do share the sentiments of the author: Like the author, I disagree "with the assessment ... that the current manuscript is not a sufficient advancement over Kim (2023)", for the reasons given by the author. I also do not think the article is too mathematical or technical and should rather be submitted to eg the journal "Theoretical Population Biology". However, I think that the present version is far from publishable. In this respect, I agree with some of the reviewers and share their frustration.

>> Thanks to your critical assessment, I could find problems in texts that were clarified and improved in presentation, as described below.

As this is an interdisciplinary study, I should maybe specify my background: I have published in population genetics, both articles that were data-heavy as well as theory-heavy. I should have no problems following the math in the present article. I have worked and published in various fields as a statistical consultant and co-written articles in those fields. I am therefore very aware of the difficulties working in interdisciplinary contexts and, especially, how much care and thinking must be invested in appropriately identifying the relevant readers, in choosing the right language, and in finding the correct terms and definitions that are acceptable in all relevant fields. It is also important to be very clear and precise, because otherwise readers of one or the other field may be lost completely. An example for an article that achieves this feat admirably is that of Bruce Wallace (1975), cited in the present article.

Unfortunately, the present article is very confusing. Like one of the earlier reviewers, I found the abstract already problematic; eg: "Recent study found that, under pre-existing oscillation in population size, the mutant is positively selected to fixation if its fitness change correlates with the rate of population growth, which further amplifies population size oscillation." This sentence should likely be split into three or more sentences. For a population geneticist the

fitness of a mutant compared to the wild-type (allele, since haploid individuals are assumed throughout) would make sense. What then does the "fitness change" of a mutant allele mean? And why should that correlate with the population size? An oscillating population size should not grow (on average); so why "the rate of population growth"? Hence, already the abstract confused me.

>> I extensively rewrote the Abstract, removing or breaking down the sentences you pointed out. Please find the track-change version of new manuscript.

The introduction did not help either, so I hoped for some clarity when the mathematical models were described. But here I was also left confused:
Fig 1. should provide an illustration for the model. I found the graphs reasonable, but the legend problematic, eg: "Reproduction, followed immediately by mutation and reproduction,..." Here the second "reproduction" makes no sense to me.

>> It was a mistake: reproduction (x) → recombination (o)

Before the passage starting at l202, populations sizes in the two patches, field F and refuge R, were described and denoted with symbols $N_F^{(t)}$ and $N_R^{(t)}$. Obviously, these are both finite. But consider: "The model so far described changes in the number of individuals without genetic drift. When a finite population size is assumed, as in simulation below, each parent in both subpopulations is modeled to produce a Poisson number of offspring with the mean specified above, which approximates genetic drift in the Wright-Fisher model." As genetic drift is zero only if the (effective) population size is infinite, the first sentence makes no sense, given that just before the finite population sizes $N_F^{(t)}$ and $N_R^{(t)}$ were described and effective population sizes are usually much smaller than census population sizes. Furthermore, the Wright-Fisher (WF) model usually considers diploid individuals; without selection a binomial distribution with mean offspring number of 2 per individual is assumed implicitly. If a haploid version of the Wright-Fisher model is assumed (as would be appropriate for the assumptions of the author), the offspring distribution per individual is again binomial with mean one. For large population sizes, the binomial distribution could be approximated by a Poisson distribution. In both cases, the strength of drift is inversely proportional the (effective) population size. In any case, the statement that "a Poisson number of offspring" approximates "genetic drift in the Wright-Fisher model" is at least confusing, if not simply wrong.

>> Now I made it clear that the model assumes finite population sizes but in the derivation of analytic approximations the changes due to genetic drift is ignored. In addition, I state that reproducing a Poisson number of offspring is not to approximate the Wright Fisher model but to set the variance of offspring number similar to that in the haploid version of the W-F model: "The model so far described only the expected changes in the numbers of A_1- and A_2-carrying individuals. However, as reproduction in a finite-sized population was implicitly assumed, how genetic drift occurs need to be specified for stochastic simulation. To set the

variance of offspring number similar to that in the haploid version of Wright-Fisher model, each parent in both subpopulations is modeled to produce a Poisson number of offspring with the mean specified above." (l. 214-220). "For convenience in the derivation, only the expected changes in the numbers of A_1- and A_2-carrying individuals will be tracked while ignoring genetic drift." (l. 237-239)
"

Close to the "heart" of the model is the stochastic variation of the key parameters S_t, S_t^*, U_t and V_t. We are informed that the expectations of all these variables are 0, and that the variances of S_t and S_t^* are positive (in the paragraph starting at l158). But we are not informed about the distribution of these two variables. On the other hand, we are informed that U_t and V_t are drawn from a joint normal distribution (paragraph starting with l207) without information on their variance-covariance matrix. Immediately, below an inequality relation of the covariances is given: $\Cov(S_t,U_t-V_t) > \Cov(S_t^*,U_t-V_t) $. Should we deduce from this that all four variables are jointly normally distributed with zero means and some arbitrary Var-Cov matrix, as long as the inequality is fulfilled? Actually in formula 2, $\Cov(S,S*)$ appears as a term. So: maybe yes?!?

>> Now, readers are informed that the specific forms of distribution is needed for simulation but not for deriving analytic solutions: "The fitness of an individual carrying A_2 relative to A_1 is given by $1 + S_t$ in the field and $1 + S_t^*$ in the refuge, where S_t and S_t^* are drawn each generation from a joint distribution with $\mathrm{E}[S_t] = \mathrm{E}[S_t^*] = 0$. While the specific form of the distribution is needed for simulation (below), for deriving analytic solution in the next section only the mean, variance, and covariance of the distribution will be needed. " (l. 174-178). Then, it is made clearer that four variables U_t, V_t, S_t and S_t^*, are all sampled together from a joint probability distribution. "More specifically, fluctuations occur such that $K_F^{(t)} = K_{F0}\mathrm{e}^{U_t}$ and $K_R^{(t)} = K_{R0}\mathrm{e}^{V_t}$ where U_t and V_t, together with S_t and S_t^*, are drawn each generation from a joint normal distribution satisfying $\mathrm{E}[U_t] = \mathrm{E}[V_t] = \mathrm{E}[S_t] = \mathrm{E}[S_t^*] = 0$. … Various assumptions can be made regarding the variance-covariance relationship among S_t, S_t^*, U_t, and V_t." (l. 224-229)

There is a further problem with S_t and S_t^*: Should S_t be less than 1, $1- S_t$ is negative and therefore the carrying capacity would also become negative (see the formulas in Appendix A), which is non-sensical, but if the two parameters are taken from a normal distribution this would be possible as the support of the normal distribution is from minus to plus infinity. The solutions would be the same as for the variables U_t and V_t: assume a log-normal distribution for S_t and S_t^* and then to use e^{S_t} instead of $1-S_t$ (the former approximates the latter anyway as long as $S-t$ is small). As the problem of negative carrying capacities may have messed up the simulations the author might have assumed that anyway. If the mathematics would have been described with the necessary rigor, including the description of the support of the random variables, this would have become obvious right away.

>> This problem was indeed recognized in early trials of simulations. The distribution of S_t or S_t^* is truncated at the lower bound -0.999 so that the fitness cannot go negative. This was mistakenly not stated in the previous version but now stated in the revised manuscript. (l. 303-304.)

The analytical results presented in Appendix A are deterministic in the allele frequencies. This fact should be pointed out in the main text. Note that this assumption leads to the paradoxical situation that a fluctuating population size is assumed to be infinitely large (such that the drift can be assumed to be zero). This problem (that I already stumbled on above) should be discussed.---In l333ff, the author actually shows that the simulations can reproduce the key predictions of the deterministic analysis. Given the assumptions, this should break down if the population sizes are very small and drift correspondingly large. Indeed, this seems to be confirmed, as the results for small population sizes are indistinguishable from neutrality. Hence, the analytical and simulation results seem to agree to the expected degree, which is reassuring.

>> Again, I made it clearer that a finite population is assumed (l. 216) and that "only the expected changes in the numbers of A_1- and A_2-carrying individuals will be tracked while ignoring genetic drift." (l. 237-239). In simulations, I made it clear that genetic drift is added to the previous step of simulation in which only deterministic allele frequency changes are tracked (l. 318). Then I mention that the results are consistent with genetic drift weakening the effect of selection (l. 327).

Generally, this impression of reassuring solidity hidden beneath a sloppy verbal description held for much of the results section; the discussion also seems generally OK. In summary, the article is far from readable. At least this reader with a popgen, math, and interdisciplinary background could not follow key parts in the abstract, the introduction and the model sections. Nevertheless, the analytic and simulation results are interesting, agree sufficiently to appear solid and seem interesting enough for eventual publication. The discussion also contained some useful further information.

>> I hope the above and other changes made in revision have improved the readability of the manuscript.

l193: "First, under soft selection, very strong density regulation occurs so that the final size of the field is constrained to $ _ $." The strength of density regulation under soft selection depends on the absolute number of individuals before density regulation. If the latter number is below the maximal capacity, no individuals would need to die or be excluded from reproduction. As a surplus of offspring is a prerequisite for evolution, this case may be considered degenerate, but at least possible.

>> Now I removed “very strong density regulation” that causes confusion. I think the earlier explanation made for soft selection gave a sufficient information: “First, under soft selection, the final size of the field is constrained to $K_F^{(t)}$.” (l. 206)

In the paragraph starting at l207: "The oscillation of carrying capacities is therefore symmetrical in the log scale, which is generally predicted in demographic models and observed in nature..." I guess that the symmetry is assumed (not predicted) in theoretical demographic models. And I consider it impossible to "observe" this in nature, although (most) data may be "consistent with" the assumption.

>> It indeed appears that the estimated population density, as described in Myers (1988), is compatible with symmetry in the log scale.

Why is it assumed that subadults are reproducing? I would define adults as individuals that have reached reproductive age, while subadults are below that. Maybe "young adults" and "old adults" would make more sense here. This model seems similar to that presented by Cenik and Wakeley (2010) PLoS One 5(9):e13019. (But those authors only consider the popgen part, not the ecological part or the feedback between the two as the present author. This shows again that the basic idea of the author is indeed interesting and worthwhile to follow up.)

>> Terms subadult and adult indeed mean young and old among reproducing individuals. Now it is made clearer (l. 152-153, 450-451). Have two age classes among reproducing adults is essential for the storage effect, where the old adults act as a storage of mutants not immediately removed under an unfavorable environment in the immediately preceding round of selection (which affect the subadult stage).
