# Supplementary material for: Noncovarying storage effect: Balancing and positive directional selection on mutant alleles that amplify random fitness and demographic fluctuations
Source: PLoS One. 2025 Jul 22;20(7):e0328130. doi: 10.1371/journal.pone.0328130 (PMC12282919; doi:10.1371/journal.pone.0328130)
Supplement: Attachment — Submitted filename: POneResponsToReviewR1.pdf [file pone.0328130.s008.pdf]

Response to reviewer comments (PONE-D-24-54939R1)

I revised the manuscript following the comments provided by three reviewers. Below each review comment (in italic) is followed by my response.

Yuseob Kim

Ewha Womans University

Editor's comment:

One of the original reviewers is satisfied with the revision whereas the other (reviewer#1) raised concerns that his original comments were not adequately addressed ("My understanding is that variation is maintained only under a set of very restricted conditions, hence the focus should be on the difficulty to maintain variation under fluctuating selection, rather than portraying it as a realistic scenario."). The additional reviewer was very positive about the manuscript. I also went over it carefully myself. I want to point out at this point that PLOS ONE's policy is to publish research based on scientific rigor, rather than impact, novelty, or relevance. Having said that, I see the point of reviewer #1. My own impression is that the NSE is motivated by ecology, but the model analyzed is haploid. Hence my impression was that there is a bit of a stretch in promoting NSE as a common and relevant mechanism. Hence, I would like to invite you to submit a revised version of the manuscript, in which you focus more on the mechanism itself and intuitive explanations of why NSE serves as a mechanism of balancing selection rather than promoting its relevance in ecology. It would be good if you could give some specific examples on when NSE can arise for haploid organisms. I also think you could add some intuitive explanations of how the underlying mechanism works in some places. Since two reviewers were satisfied, please keep in mind to keep the revision "minimally invasive"

>> First, I can understand the concern by reviewer 1 but I was hoping for a new comment indicating whether he/she agrees, or not, with my argument and revisions in response to the original comment. Furthermore, since I responded to the original comment while submitting the revised version, I obtained further results supporting my argument that NSE is not a process making balancing selection more restrictive in nature but, on the contrary, a novel force that leads to polymorphism of previously ignored, but abundantly available (i.e., net deleterious) variants. This new findings were written in a manuscript "Maintenance of net deleterious alleles in polymorphism under the joint fluctuation of demography and selection in age-structured populations" and was just submitted to Transactions R Soc B. I uploaded the submitted manuscript as a supplemental file as a reference. Below I explain this point to reviewer 1 in detail but did not make a large change in the manuscript because I cannot report the result in two papers simultaneously.

Regarding your concern about the applicability of my haploid models to diploid species, I added the following paragraph at the end of Introduction: "Although the majority of species to which the ecological scenarios of the TP and LSA models apply are diploids, haploid models will be constructed and analyzed for mathematical convenience. However, because the final outcomes

of the eco-evolutionary dynamics are predicted by changes in allele frequency shortly after a mutant allele arises and when its frequency increased near fixation (i.e., whether a rare allele can invade the population), the results of this study using the haploid models should be relevant to diploid species as well. In a diploid species, homozygotes for the rare allele can be ignored. Consequently, the relative fitness of the rare allele in the haploid model corresponds to the fitness of heterozygotes relative to common homozygotes in the diploid population."

Reviewers' comments:

Reviewer #1: I was not convinced that the minor changes to the text reflect my different perception of the message. Hence, I think that the results as presented portray the wrong impression and the ms may be cited for the misleading message.

>> The previous review comment was "*My understanding is that variation is maintained only under a set of very restricted conditions, hence the focus should be on the difficulty to maintain variation under fluctuating selection, rather than portraying it as a realistic scenario. As written, the reviewer feels that the study may be cited for evidence that variation is maintained under fluctuating selection, although most results show the opposite.*" I can accept this conclusion only if a new model in this study incorporating demographic fluctuation, the noncovarying storage effect, clearly predicts that balanced polymorphism is less likely in natural populations than was predicted by the previous models of the storage effect. I made it clear in Discussion that the apparent reduction in the parameter range for balanced polymorphism, for example the failure of NSE to maintain GMF mutants in polymorphism, is that the model considers only special patterns of mutants' fitness fluctuation, that is, the case of mutants' geometric or arithmetic mean fitness being equal to that of the wild-type allele. Based on the heuristic argument for the mechanism of balancing selection in the proposed model, I suggested that mutations with even smaller arithmetic mean fitness, thus net deleterious mutations, will probably be positively selected at low frequency and then maintained in polymorphism with heavy fluctuation in the carrying capacity of the field, which however restricts the condition for polymorphism with AMF mutations. If true, more genetic variation is predicted to be maintained by the NSE than by the conventional model of the storage effect (without the demographic fluctuation), because mutations of net deleterious effects are more likely to arise than GMF or AMF mutations (see below).

After I wrote the rebuttal and submitted the revised manuscript in February, I further elaborated the argument above and confirmed the maintenance of net deleterious alleles in polymorphism due to NSE using new age-structure models. This work was written in a new manuscript titled "Maintenance of net deleterious alleles in polymorphism under the joint fluctuation of demography and selection in age-structured populations" and was submitted to *the Philosophical Transactions of the Royal Society B*. To prevent overlap between publications, these new analysis and results were not included in the current manuscript. Below I briefly present the new analysis. I also uploaded the submitted manuscript file to *Transactions B* for your reference.

Consider the "two-season model" in Kim (2023) in which there are two alternating seasons, good and bad, with the carrying capacity of the field alternating between K_G and K_B. The fitness of the mutant allele, A_2, relative to the wild-type is w_G in a good season and w_B in a bad season.

The probability that an allele is located in the refuge is given by r. Then, the condition for polymorphism is given by

$$\left(\frac{K_G}{N_B}w_G + r\right)\left(\frac{K_B}{N_G}w_B + r\right) > 1 \text{ and } \left(\frac{K_G}{N_B}\frac{1}{w_G} + r\right)\left(\frac{K_B}{N_G}\frac{1}{w_B} + r\right) > 1$$

where $N_G = (K_G + rK_B)/(1 - r^2)$ and $N_B = (K_B + rK_G)/(1 - r^2)$ (see eqs. (1.6) and (1.7) and Appendix D of Kim (2023)). Below the values of (w_B, w_G) that satisfy both inequalities above, thus allowing balanced polymorphism, are plotted with varying demographic fluctuations given by $k = K_G/K_B$ ($r = 0.5$).

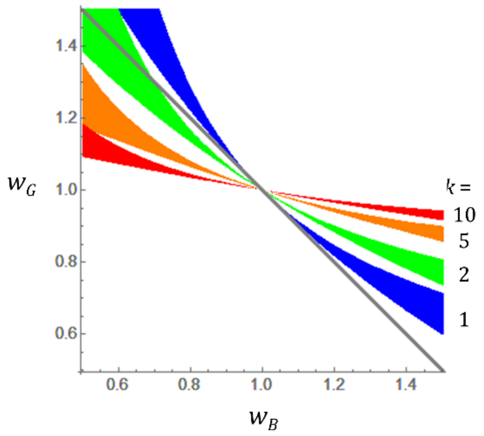

It is shown that, for $k = 1$ (thus conventional storage effect without fluctuation in the size of the field), only GMF mutants with $(w_B + w_G)/2 > 1$ can be maintained in polymorphism. AMF mutations ($(w_B + w_G)/2 = 1$, indicated by the gray line) are excluded. Then, with $k = 2$, polymorphism occurs with AMF mutations (if w_G is larger than ~1.25) but not with GMF mutants that were polymorphic with $k = 1$. This downward shift in $(w_B + w_G)/2$ as a requirement for polymorphism continues as k further increases. With $k = 5$ or 10, alleles maintained in polymorphism are no longer AMF mutants but net deleterious mutants ($(w_B + w_G)/2 < 1$).

Such net deleterious mutations are far more plausible than AMF or GMF mutation. Martin and Lenormand (2015, Evolution 69:1433), using the standard Fisher's geometric model of fitness in which two different phenotypic optima alternates in oscillating environment, showed that a mutation causing a random phenotypic change has two fitness effects (S; the selection coefficient) plotted below when the wild-type has a phenotype in between the optima (in Figure 2 of Martin and Lenormand).

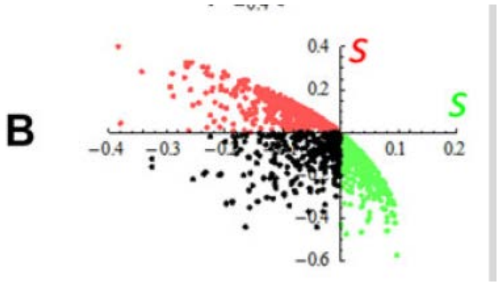

As you can see, the mean of S over the two environments is less than 0, thus the arithmetic mean of fitness (1+S) being less than 1.

Therefore, while the conventional model of the storage effect requires GMF mutations (the occurrence of which lacks empirical and theoretical support), the NSE can generate balanced polymorphism from mutations with lower arithmetic mean fitness, which are theoretically well-supported to arise. Portraying the results of this study as demonstrating the difficulty of maintaining genetic variation in fluctuating selection would thus send a misleading message to readers. Instead, the NSE should be highlighted as a novel mechanism of balancing selection capable of acting on a large number of loci.

Reviewer #2:

The writing has been improved and I could generally understand the article. The improved clarity has uncovered only minor problems with the underlying science. The manuscript still needs editing to improve language and readability and, in places, mathematical rigor (non-exhaustive comments mainly in the attached text).

>> I again made grammatical corrections and tried to improve the flow of text. Other points were addressed below

I have one general point to make: It seems to me that one side is ignored, which should be treated for the sake of clarity and completeness of the argument. I will start out with a term that is unclear: "incomplete correlation". A correlation is only rarely exactly plus or minus one (if this is meant with "complete"); is an "intermediate correlation" meant, maybe between plus or minus 0.2-0.7. While "complete correlation" may make sense (a correlation of exactly plus or minus one) and an "intermediate correlation" may be between plus or minus 0.2-0.7, "covary incompletely", i.e, an "incomplete covariance" makes less sense, as far as I can see.
*This goes beyond picking words: As far as I understand the correlation (in population size between the field and refuge) must be *positive* and intermediate; a *negative* correlation would not allow for the major point of the article. I guess that instead of balancing selection (and thus balanced polymorphism), disruptive selection should result from a negative correlation. If this is true, this possibility needs to be mentioned and the parameter ranges, where it can occur, need to be explored. I guess that this case is of little biological relevance but, at least to me, would be required to make the argument complete.*

>> I agree with your concern on this problem. Now throughout the manuscript, it was made clear that correlation means positive correlation. For example, in the Abstract, “if its fitness correlates with oscillation in the size of the field” was changed to “if its fitness and the size of the field change in the same directions”(l.23), and, “whether balanced polymorphism or fixation occurs depends … incomplete correlation between the size between the field and refuge” was changed to “… the discordant oscillation in the population sizes of the field and refuge” (l. 32). On line 272, I added “Note that, since ρ > 0, only positive covariance between S – S^* and U – V leads to positive selection on A_2.” to emphasize that only positive correlation between S and K_F/K_R leads to the negative-frequency dependent selection. Regarding the potential effect of both positive and negative correlations between K_F and K_R, I stated that “Note that this term “noncovarying” refers to “not positively correlated”, thus including the cases of negative covariance between $K_F^{(t)}$ and $K_R^{(t)}$, which lead to wide fluctuations in $K_F^{(t)}/K_R^{(t)}$ and thus large

(positive) values of Φ."(line 284-286).

I am unclear in places, eg, l207, whether an absolute frequency (a count) or a proportion is meant.

>> On l.207, it means absolute frequency, not proportion. Therefore, the number of progeny summed over all parents becomes the population size. I think in other places the context provides which is meant.

l 174 I would say that selection does not require genetic variation, but evolution (the response to selection) does.
>> To eliminate the confusion, the sentence was changed to "a new mutant allele A_2 arises."

l204 I think there is a logical error here..

>> Now it is changed to "… such that the population size can increase over or decrease below the carrying capacity of a population in which all individuals carry the ancestral (A_1) allele."

Other comments made in pop-up windows on the manuscript file were addressed as the following:

l.70, "However, recent studies suggested that temporal fluctuating selection can turn into balancing selection" >> "However, recent studies have suggested that temporally fluctuating selection can also act as balancing selection"

l.119, the present tense was used to describe the result of the past study that should be invariant over time.

l.143, 'plants that exhibit mast seeding' may not be the best example but it was cited in Kim (2023) which however considered only cyclically changing environments. So it was important to mention it as a system to which the model of randomly fluctuating environment is more relevant.

l.158. Corrected.

l.216. "However, as reproduction in a finite-sized population was implicitly assumed, how genetic drift occurs need to be specified for stochastic simulation." >> "However, as reproduction in a finite-sized population was implicitly assumed, the role of genetic drift needs to be investigated. Stochastic simulation will be used for this investigation. In simulation, to set the variance of …"

l.227. "generally predicted in demographic models and observed in nature" >> "generally predicted in demographic models and is also consistent with empirical observation"

l.312. Changed as suggested.

l.333. Since the A2 allele increases at all frequencies, it is not disruptive selection but directional selection, which was well described in the above. The fact that it does not approach the outcome of neutrality was also clearly mentioned.

l.336. Corrected as suggested.

l. 416. Corrected.

l.503. What AMF stands for was explained in Introduction.

l.547, l.560 Corrected.

Reviewer 3

I read through the manuscript and the response letter. I like the revised manuscript very much. I personally think that the author has addressed all concerns that were raised by the two reviewers. After the author made the revisions, the manuscript is much clearer than the first version.
In the revised manuscript, the author studied a very important question about how natural selection occurs in natural populations. Noncovarying storage effect is straightforward to describe the effect. I also like the theoretical analysis, one-locus simulation and multi-locus simulation. Therefore, I would suggest the editor to accept the manuscript in the current form.

>> I greatly appreciate this positive evaluation.
